# Proteinase 3 depletion attenuates leukemia by promoting myeloid differentiation

**DOI:** 10.1038/s41418-024-01288-4

**Published:** 2024-04-08

**Authors:** Huan Liu, Lu Sun, Hongfei Zhao, Zihan Zhao, Shiyue Zhang, Shan Jiang, Tianran Cheng, Xiaohan Wang, Tong Wang, Ya Shao, Haiyan Zhu, Huijuan Han, Yigeng Cao, Erlie Jiang, Yihai Cao, Yuanfu Xu

**Affiliations:** 1 State Key Laboratory of Experimental Hematology, National Clinical Research Center for Blood Diseases, Haihe Laboratory of Cell Ecosystem, Institute of Hematology & Blood Diseases Hospital, Chinese Academy of Medical Sciences & Peking Union Medical College, Tianjin, 300020 China; 2 Tianjin Institutes of Health Science, Tianjin, 301600 China; 3 Clinical Laboratory, The First Affiliated Hospital of Xi’an Jiaotong University, Xi’an, 710061 China; 4 The Second School of Clinical Medicine, Guangdong Medical University, Dongguan, 523808 China; 5 Department of Clinical Lab, Weihai Municipal Hospital, Weihai, 264200 China; 6 Department of Medical Laboratory, School of Clinical Medicine, Ningxia Medical University; Ningxia Key Laboratory of Clinical and Pathogenic Microbiology, General Hospital of Ningxia Medical University, Yinchuan, 750001 China; 7 Hematopoietic Stem Cell Transplantation Center, Institute of Hematology and Blood Diseases Hospital, Chinese Academy of Medical Sciences and Peking Union Medical College, Tianjin, 300020 China; 8 Department of Microbiology, Tumor and Cell Biology, Karolinska Institute, Solna, 17165 Sweden

**Keywords:** Signal transduction, Haematological diseases

## Abstract

Hematopoietic stem and progenitor cells (HSPCs) that have impaired differentiation can transform into leukemic blasts. However, the mechanism that controls differentiation remains elusive. Here, we show that the genetic elimination of Proteinase 3 (PRTN3) in mice led to spontaneous myeloid differentiation. Mechanistically, our findings indicate that PRTN3 interacts with the N-terminal of STAT3, serving as a negative regulator of STAT3-dependent myeloid differentiation. Specifically, PRTN3 promotes STAT3 ubiquitination and degradation, while simultaneously reducing STAT3 phosphorylation and nuclear translocation during G-CSF-stimulated myeloid differentiation. Strikingly, pharmacological inhibition of STAT3 (Stattic) partially counteracted the effects of PRTN3 deficiency on myeloid differentiation. Moreover, the deficiency of PRTN3 in primary AML blasts promotes the differentiation of those cells into functional neutrophils capable of chemotaxis and phagocytosis, ultimately resulting in improved overall survival rates for recipients. These findings indicate PRTN3 exerts an inhibitory effect on STAT3-dependent myeloid differentiation and could be a promising therapeutic target for the treatment of acute myeloid leukemia.

## Introduction

The differentiation of myeloid cells from progenitors is a vital process facilitated by hematopoietic cytokines, resulting in the development of granulocytes and monocytes [1, 2]. These cells are crucial for the immune system’s defense against invading microorganisms, and their deficiencies can lead to severe pathological conditions [3]. Arrest of myeloid differentiation can cause the accumulation of proliferative blasts, leading to the production of immature cells, as observed in myeloid leukemias [4]. However, differentiation therapy has shown success in treating acute myeloid leukemia (AML), particularly acute promyelocytic leukemia. This therapy promotes the maturation of AML cells and facilitates the clearance of normal mature myeloid cells [5–7]. Therefore, identifying useful genetic targets that can induce hematopoietic maturation and differentiation has the potential to be therapeutically significant across different AML subtypes.

Proteinase 3 (PRTN3) is a neutral serine protease mainly found in neutrophils and monocytes, playing a significant role in both the non-oxidative pathway of intracellular and extracellular pathogen destruction, as well as being a major component of neutrophil azurophilic granules [8, 9]. In addition to its role in controlling apoptosis [9], PRTN3 has a proteolytic-independent microbicidal activity [10] and an elastase-like enzymatic activity [3]. Moreover, it promotes the degradation of extracellular matrix and basement membrane proteins [11]. Although PRTN3 has been extensively studied in relation to its function in the maturation of myeloid cells, it is also crucial in many other cellular functions, especially those involving hematopoietic stem and progenitor cells [9, 12–14]. Nevertheless, our understanding of the mechanism and the biological significance of PRTN3 expressed in hematopoietic stem and progenitor cells to regulate myeloid differentiation is very limited.

In this study, we aimed to investigate the effect of PRTN3 deficiency on spontaneous myeloid differentiation in mice and explore the potential of STAT3 inhibitors to counteract this effect. We explored the molecular mechanisms that regulate PRTN3 in relation to STAT3-dependent myeloid differentiation both in vitro and in vivo. Furthermore, we demonstrated that PRTN3 deficiency protects against acute myeloid leukemia by promoting the resumption of myeloid differentiation in mice and humans. Our findings reveal a novel pathway by which PRTN3 regulates myeloid differentiation by downregulating STAT3 expression, indicating a promising new therapeutic target for the treatment of acute myeloid leukemia.

## Results

### Depletion of PRTN3 induces myeloid differentiation in mice

**Fig. 1:**
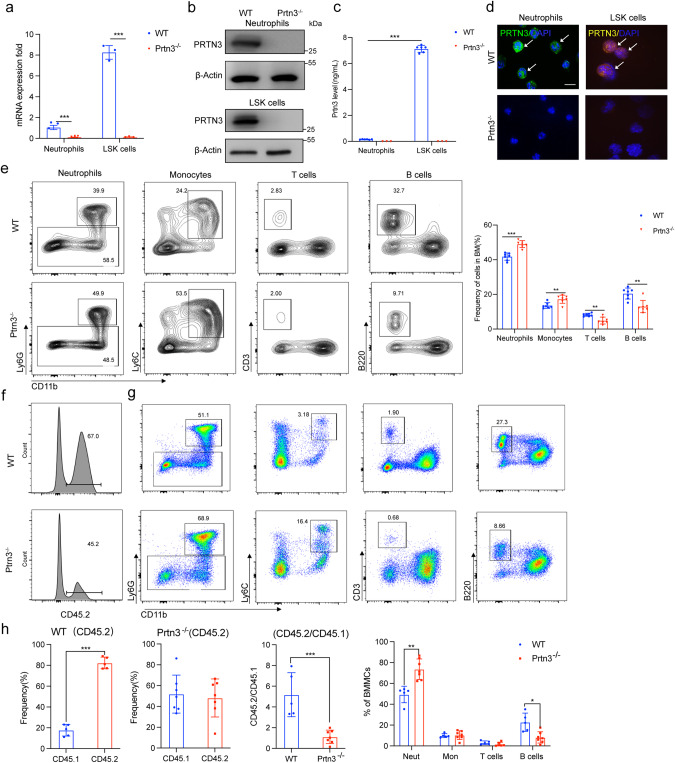
PRTN3-deficiency induces myeloid differentiation in mice. **a**–**d** messenger RNA (mRNA), Protein level detected by WB and ELISA, and immunofluorescence staining of *Prtn3* were determined in neutrophils and LSK cells from *Prtn3*^*−/−*^ and WT mice (*n* = 3); **e** Representative flow cytometry plots for neutrophils, monocytes, T cells and B cells in bone marrow from WT and *Prtn3*^*−/−*^ mice. Numbers denote the frequency of each population among live singlets(*n* = 7). **f** Representative flow cytometry plots for neutrophils, monocytes, T cells, and B cells in bone marrow from the CD45.2 cells in CD45.1 recipient mice (*n* = 7). Numbers denote the frequency of each population among live singlets. **g**, **h** Quantification of the frequency of CD45.1, CD45.2, the ratio of CD45.2/CD45.1, neutrophils, monocytes, T cells, and B cells in the CD45.2 recipient mice (*n* = 7). scale bars, 50 μm; *p < 0.05, **p < 0.01, ***p < 0.001. Data are the mean ± s.d.; *n*: biologically independent experiments. Statistical analysis was performed using an unpaired two-tailed Student’s *t* test.

To determine PRTN3’s expression in hematopoietic cells, we conducted a data mining analysis using the Haemopedia RNA-seq data sets (https://www.haemosphere.org) to determine PRTN3’s expression in hematopoietic cells. The findings revealed that PRTN3 is expressed throughout hematopoiesis, with the highest levels detected in multi-potential progenitor cells, restricted potential progenitor cells, and the myeloid lineage (Fig. S1). To investigate whether PRTN3 deficiency affects myeloid differentiation, we generated global *Prtn3* knockout mice (*Prtn3*^*−/−*^) and confirmed *Prtn3* deletion in these mice using various methods, including RT-qPCR, Western blot, ELISA assay, and immunofluorescence staining (Fig. 1a-d). Harvesting bone marrow and flow cytometry to identify different cell populations, we observed an increase in myeloid populations, neutrophil and monocyte, and a decrease in T and B cell populations in *Prtn3*^*−/−*^ mice compared to their WT littermates (Fig. 1e). Additionally, through routine analysis and flow cytometry, we found that *Prtn3*^*−/−*^ mice exhibited a gradual exacerbation of spontaneous increases in myeloid cell populations, specifically neutrophils and monocytes, and decreases populations of T cell, in bone marrow, spleen, and peripheral blood (Fig. S2a-d). We conducted a series of assays to assess T cell functions, including the CFSE T cell proliferation assay, T cell activation bioassay, and T cell killing assay. Interestingly, our findings revealed that the proliferation, activation, and anti-AML immunity of T cells were not affected in *Prtn3*^−/−^ mice, compared to their WT littermates. (Fig. S3a-c). Next, we examined the number of LK and LSK cells and found a significant increase in the percentage of these cells in *Prtn3*^*−/−*^ mice, compared to WT mice from 24 weeks to 72 weeks (Fig. S4a, b). We further examined the percentage of GMP, CMP, MEP, MPP, ST-HSC, and LT-HSC in both WT and *Prtn3*^*−/−*^ mice throughout the aging process using flow cytometry. Our analysis revealed a significant increase in *Prtn3*^*−/−*^ mice from 24 weeks to 72 weeks, compared to WT mice, except for MEP (Fig. S4c, d). We also cultured LSK cells from mice of varying ages on metho-cult GM 3434 medium and found no change in colony-forming ability for all colony types (G, M, GM, and GEMM) at stages 4, 8, 24, 48, and 72 weeks of isolated-primary cells for both WT and *Prtn3*^*−/−*^ mice (Fig. S5). To confirm that PRTN3 affects myeloid differentiation in LSK cells rather than downstream cells, we generated *Prtn3* conditional knockout (*Prtn3*^*LyzKO*^) mice by crossing *Prtn3*^*flox/flox*^ mice with LysM^Cre^ mice. The percentage of myeloid cells was analyzed through flow cytometry, and the results showed no difference between WT and *Prtn3*^*LyzKO*^ mice in the percentage of neutrophils, monocytes, T cells, and B cells in the bone marrow, spleen, and peripheral blood (Fig. S6). Thus, we conclude that *Prtn3* deficiency in mice induces advanced spontaneous myeloid differentiation, and PRTN3 is crucial for myeloid differentiation in LSK cells.

To investigate whether the lack of *Prtn3* promotes the differentiation of LSK cells into myeloid cells in mice receiving transplants, we sorted 1 × 10^3^ LSK cells from both WT and *Prtn3*^*−/−*^ mice [C57BL6-Ly5.2/CD45.2(C57)] at 8, 48, and 72 weeks old, and mixed these cells with 5 × 10^5^ bone marrow cells from C57BL6-Ly5.2/CD45.1(B6) mice, which we then injected into C57BL6-Ly5.2/CD45.1(B6) mice that had undergone 9.5 Gy radiation. After 16 weeks post-transplant, we analyzed chimerism in the bone marrow and spleen (Fig. S7a). Routine analysis of blood showed a higher percentage of neutrophils in the peripheral blood of *Prtn3*^*−/−*^ mice than in WT mice at different ages (Fig. S7b, Fig. S8a, and Fig. S9a). Flow cytometry analysis revealed that the ratio of CD45.2/CD45.1 was decreased, while the myeloid population was elevated in bone marrow (Fig. 1f, g, h, Fig. S8b, Fig. S9b), spleen (Fig. S7c, Fig. S8c, Fig. S9c), and peripheral blood (Fig. S7d, Fig. S8d, Fig. S9d) of *Prtn3*^*−/−*^ mice compared to WT mice at various ages. Furthermore, we observed an increase in the percentage of various cell types, including LK cells, LSK cells, GMP, CMP, MEP, MPP, ST-HSC, and LT-HSC, in *Prtn3*^*−/−*^ mice compared to WT mice, and this increase was age-dependent (Fig. S7e, Fig. S8e, Fig. S9e). In addition, the immunofluorescence assay showed lower chimerism with donor-derived cells from *Prtn3*^*−/−*^ mice in the recipient mice (Fig. S7f, Fig. S8f, Fig. S9f). However, we did not observe a significant change in the number of colonies formed, including G, M, GM, and GEMM, when we cultured LSK cells from WT and *Prtn3*^*−/−*^ mice of different ages (Fig. S7g, h, Fig. S8g, Fig. S9g). Our results suggest that PRTN3 plays a crucial role in the myeloid differentiation of LSK cells.

### PRTN3 directly interacts with STAT3

**Fig. 2:**
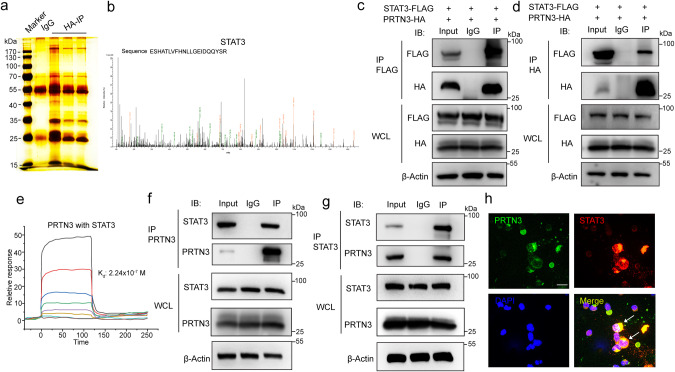
PRTN3 directly interacts with STAT3. **a** The Co-immunoprecipitation (Co-IP) complex was subjected to silver-staining (*n* = 3); **b** the peptide sequences of the STAT3 protein as detected in the Co-IP complex by mass spectrometry; **c** WB analysis of Co-IP complex confirmed that STAT3-Flag interacted with PRTN3-HA in HEK 293 T cells (*n* = 3); **d** WB analysis of Co-IP complex confirmed that PRTN3-HA interacted with STAT3-Flag in HEK 293 T cells (*n* = 3); **e** measurements of the binding affinity between STAT3 and PRTN3 by SPR method. **f** WB analysis of Co-IP complex confirmed that STAT3 interacted with PRTN3 in CD34+ cells (*n* = 3); **g** WB analysis of Co-IP complex confirmed that PRTN3 interacted with STAT3 in CD34+ cells (*n* = 3); **h** the immunofluorescence showed that STAT3 colocalized with PRTN3 and were expressed in both the cytoplasm of CD34+ cells (*n* = 3). Data are the mean ± s.d.; *n*: biologically independent experiments. Statistical analysis was performed using an unpaired two-tailed Student’s *t* test.

To explore the molecular mechanism underlying myeloid differentiation and the potential factors that interact with PRTN3, we conducted immunoprecipitation (IP) assays and utilized high-sensitivity mass spectrometry screening. Specifically, we investigated the binding of PRTN3 to potential proteins in HEK293T cells that overexpressed PRTN3 by transfecting an HA-tagged PRTN3 plasmid and subsequently pulldown with the HA antibody. After screening over 5000 peptides, we identified one STAT family protein, STAT3, that may be linked to PRTN3 and the regulation of myeloid differentiation (Fig. 2a, b). We then co-transfected Flag-tagged STAT3 and HA-tagged PRTN3 into HEK293T cells, immunoprecipitated the cell lysates with anti-Flag or anti-HA antibodies and immunoblotted the Co-IP complex. Our results indicated that STAT3 co-immunoprecipitated with PRTN3 but not with IgG in HEK293T cells (Fig. 2c, d). Furthermore, we investigated whether PRTN3 could bind to other STAT family proteins by conducting experiments in which we overexpressed PRTN3 in HEK293T cells and tagged it with HA. We then immunoprecipitated the cell lysates using anti-STAT1, anti-STAT2, and anti-STAT5 antibodies. Interestingly, we observed that PRTN3 was unable to bind to STAT1, STAT2, and STAT5 (Fig. s10a). We also investigated whether neutrophil elastase (NE) and cathepsin G (CatG), which are active forms of neutrophil serine proteinases like PRNT3, could interact with STAT3. Specifically, we added STAT3 antibody into HL60 cell lysates for immunoprecipitation with anti-NE and anti-CatG antibodies. The results indicated that there was no binding between STAT3 and NE or CatG (Fig. s10b). Moreover, the results of the surface plasmon resonance (SPR) measurement showed that PRTN3 and STAT3 had a direct binding interaction in vitro, with a KD value of 2.24 × 10^−7 ^M (Fig. 2e). We then confirmed these findings in primary cells by conducting Co-immunoprecipitation (Co-IP) experiments on human primary CD34+ cells, which were isolated from cord blood using CD34-labeled beads. Our results demonstrated that endogenous PRTN3 also directly interacted with STAT3 in primary cells (Fig. 2f, g). Additionally, confocal immunofluorescence analysis revealed that PRTN3 and STAT3 were co-localized in the cytoplasm of human CD34+ cells (Fig. 2h). These findings strongly suggest that PRTN3 interacts with STAT3 and could potentially play a role in regulating STAT3’s fate.

### PRTN3 binds to the N-terminal domain of STAT3 to identify the cleavage site

**Fig. 3:**
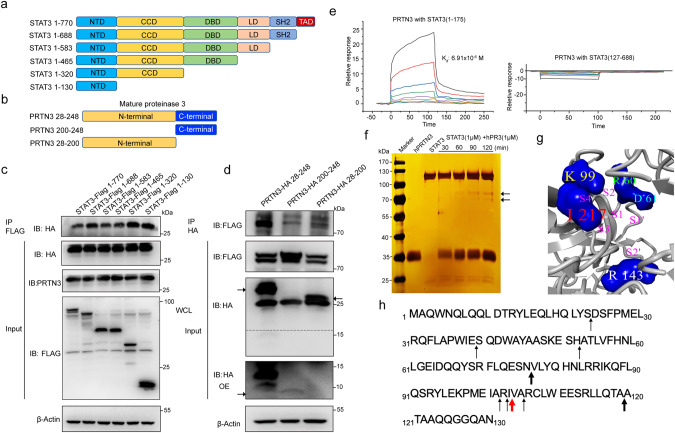
PRTN3 binds STAT3’s N-terminal domain to identify cleavage sites. **a** The schematic diagram of human STAT3 and its truncated mutants. **b** The schematic diagram of human PRTN3 and its truncated mutants. **c** Flag-tagged STAT3 or its mutants and HA-tagged PRTN3 were co-transfected into HEK293T cells. Cell lysates were immunoprecipitated with an anti-Flag antibody and then immunoblotted with the indicated antibodies (*n* = 3). **d** HA-tagged PRTN3 or its mutants and Flag-tagged STAT3 were co-transfected into HEK293T cells. Cell lysates were immunoprecipitated with an anti-HA antibody and then immunoblotted with the indicated antibodies (*n* = 3). **e** measurements of the binding affinity between the different domains of STAT3 and PRTN3 by SPR method. **f** SDS/PAGE analysis of hPR3-generated fragments of STAT3. At different times, the incubation mixtures were separated on a 10% SDS/PAGE gel denatured/reduced conditions and visualized by silver nitrate staining (*n* = 3). **g** The solvent-accessible surface of the hPR3 active site was generated with Yasara (http://www.yasara.org). **h** Identification of the cleavage sites within the whole STAT3 after incubation with PRTN3 at pH 7.4 and 37 °C. Cleavage products were monitored by MALDI-TOF MS. Arrows indicate the cleavage sites of STAT3 that were identified. The width of the arrows indicates the intensity of the cleavage. Data are the mean ± s.d.; *n*: biologically independent experiments. Statistical analysis was performed using an unpaired two-tailed Student’s *t* test.

Based on the structural features that have been previously reported, STAT3 is composed of several domains, including an N-terminal domain, linker domain, coiled-coil domain, DNA-binding domain, Src Homology 2 domain (SH2), C-terminal domain and transactivation domain [15, 16]. To investigate the domains of STAT3 responsible for interacting with PRTN3, we generated Flag-tagged STAT3 mutants and transfected them into HEK293T cells (Fig. 3a). Subsequently, we performed IP with an anti-Flag antibody and immunoblotting with an anti-HA antibody. Our analysis showed that the N-terminal domain of STAT3 played a significant role in its interaction with PRTN3 (Fig. 3c). Next, we generated an HA-tagged PRTN3 plasmid that included the full-length, N-terminal, and C-terminal domains (Fig. 3b), then performed a Co-IP experiment with Flag-tagged STAT3, which validated our findings (Fig. 3d). In addition, we conducted an analysis to confirm the KD value of STAT3 with PRTN3 using recombinant human STAT3 with and without the N-terminal domain. The results from surface plasmon resonance (SPR) indicated that the N-terminal domain of STAT3 had a direct binding affinity to PRTN3 (KD = 6.91 × 10^−6 ^M), in contrast to other domains of STAT3 (Fig. 3e).

Next, we used the pure recombinant human STAT3 and PRNT3 protein for co-incubation to test whether PRTN3 could degrade STAT3 in vitro. After incubating STAT3 (1 μM) with hPRTN3 (1 μM), we observed the processing of the ~120 kDa monomer into two major fragments: ~90 kDa, as revealed by SDS-PAGE and silver staining (Fig. 3f). We predicted the presence of PRTN3 cleavage sites in the N-terminal region segment of STAT3 and identified at least twenty-seven potential cleavage sites, denoted by an asterisk. Among these sites, we found that Val (total 3) and Ala (total 13) were the preferred P1 residues for PRTN3 (Fig. 3g, Fig. s11a, b). The cleavage products were identified through mass spectrometry. The results revealed variations in the cleavage site, and a hypersensitive site (Ala102 ↓-Arg103 ↓-Ile104 ↓-Val105 -Ala106 ↓-Arg107) was found in the N-terminal domain of STAT3 (Fig. 3f). Therefore, these results indicate that PRTN3 binds directly to the N-terminal domain of STAT3 and cleaves the hypersensitive region.

### PRTN3 promotes the degradation of STAT3-dependent of ubiquitination

**Fig. 4:**
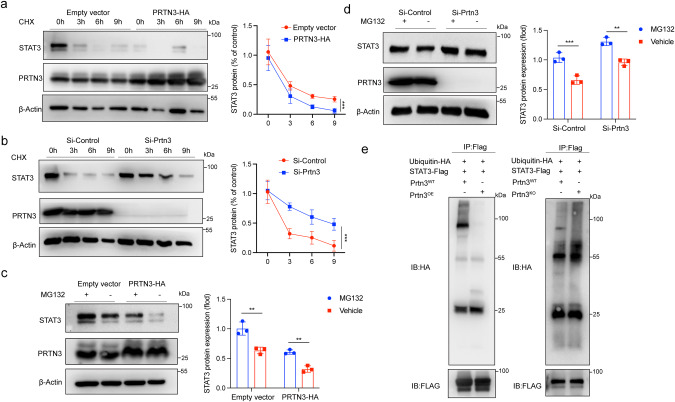
PRTN3 reduced the stability of STAT3 dependent on ubiquitination. **a** HEK293T transfected with empty vector or PRTN3-HA were treated with cycloheximide (100 ng/mL) for the indicated periods of time (*n* = 3); **b** human CD34+ cells transfected with Si-Control or Si-*Prtn3* were treated with cycloheximide (100 ng/mL) for the indicated periods of time (*n* = 3); **c** HEK293T transfected with Empty vector or PRTN3-HA were treated with or without MG132 (50 μM) for 6 h (*n* = 3). **d** human CD34+ cells transfected with Si-Control or Si-*Prtn3* were treated with or without MG132 (50 μM) for 6 h (*n* = 3). **e** In vitro ubiquitination assay of Empty vector, PRTN3-HA transfected to HEK293T, Si-Control or Si-*Prtn3* transfected to human CD34+ cells. Cell lysates were immunoprecipitated with anti-FLAG antibody followed by immunoblotting analysis with anti-HA or anti-FLAG antibody (*n* = 3). *p < 0.05, **p < 0.01, ***p < 0.001. Data are the mean ± s.d.; *n*: biologically independent experiments. Statistical analysis was performed using an unpaired two-tailed Student’s *t* test.

We examined the levels of *Stat3* mRNA in cells with overexpression or lack of PRTN3 and did not find any significant differences in *Stat3* mRNA levels between the two groups (Fig. S12). Thus, we hypothesized that PRTN3 might regulate the protein stability of STAT3 and conducted HEK293T cells were transfected with either PRTN3-HA tag or an empty vector for 24 h. Subsequently, the cells were treated with the protein synthesis inhibitor cycloheximide (CHX) for a time-dependent period. The results of CHX chase assay showed that endogenous STAT3 in cells overexpressing PRTN3 had a significantly shorter half-life (Fig. 4a). We also observed a significant increase in the half-life of endogenous STAT3 in down-regulated PRTN3 of human CD34+ cells following siRNA treatment (Fig. 4b), indicating that PRTN3 plays a crucial role in decreasing STAT3 stability in vitro.

To determine whether PRTN3 plays a role in regulating STAT3 stability through inhibition of proteasome degradation, we found that overexpression of PRTN3 through PRTN3-HA tag transfection in HEK293T cells resulted in a reduction in the protein level of STAT3. This reduction was subsequently recovered by treatment with proteasome inhibitor MG-132 (Fig. 4c). Conversely, depletion of PRTN3 in human CD34+ cells resulted in upregulation of STAT3 protein, which was rescued by the addition of MG132 (Fig. 4d). These results suggest that PRTN3 plays a role in regulating STAT3 stability through the inhibition of proteasome degradation. We then investigated whether PRTN3 affected the stability of STAT3 through ubiquitination and observed a significant decrease in the ubiquitin-mediated degradation of STAT3 in HEK293T cells that overexpressed the PRTN3-HA tag (Fig. 4e). Conversely, downregulation of PRTN3 in human CD34+ cells resulted in an Increase in the levels of STAT3 protein ubiquitination (Fig. 4e). Collectively, these results strongly suggest that PRTN3 plays a critical role in stabilizing STAT3.

### PRTN3 negatively regulates STAT3-dependent myeloid differentiation

**Fig. 5:**
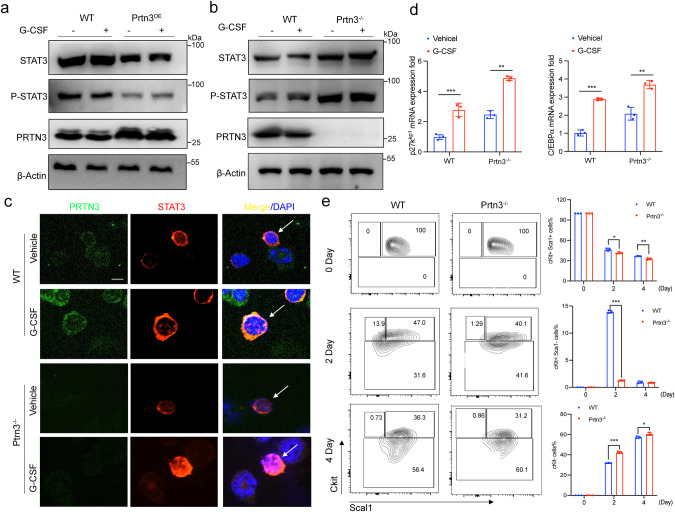
Deficiency of PRTN3 promotes STAT3-dependent myeloid differentiation. **a** protein expression of STAT3, P-STAT3, PRTN3, and β-Actin were analyzed in HEK293T cells with empty vector or PRTN3-HA transfection to overexpress PRTN3 and then treated with 10nM G-CSF for 24 h (*n* = 3); (b)protein expression of STAT3, P-STAT3, PRTN3, and β-actin were analyzed in *Prtn3*^−/−^ primary Ckit+ cells with G-CSF treatment for 24 h, compared to WT cells (*n* = 3); **c** immunofluorescence staining of STAT3 in *Prtn3*^−/−^ primary Ckit+ cells with G-CSF treatment for 2 h (*n* = 3); **d** messenger RNA (mRNA) of *P27*^*kpi1*^ and *C/Ebpα* were determined in *Prtn3*^−/−^ primary Ckit+ cells with G-CSF treatment for 24 hours, compared to WT cells (*n* = 3); **e** Lineage- Sca-1+c-Kit+ (LSK) cells from WT and *Prtn3*^−/−^ mice were used for ex vivo myeloid culture (*n* = 3). scale bars, 50 μm; *p < 0.05, **p < 0.01, ***p < 0.001. Data are the mean ± s.d.; *n*: biologically independent experiments. Statistical analysis was performed using an unpaired two-tailed Student’s *t* test.

Previous studies have demonstrated that G-CSF induces the activation of STAT3, which promotes myeloid differentiation both in vitro and in vivo [1, 17]. To investigate the physiological role of PRTN3 in STAT3-dependent process. We treated HEK293T cells that overexpressed either wild type or PRTN3 with 10nM G-CSF for 24 h. Immunoblotting assay indicated that PRTN3 upregulation led to a significant decrease in both total STAT3 and phosphorylated STAT3 expression in HEK293T cells treated with G-CSF, compared to WT cells (Fig. 5a). However, to further explore the involvement of PRTN3 in G-CSF induced STAT3-regulated myeloid differentiation, we utilized primary c-Kit+ cells isolated from both *Prtn3*^*−/−*^ and WT mice, following a 24-hour treatment with G-CSF. We observed an increase in both STAT3 and P-STAT3 expression in *Prtn3*^*−/−*^ primary c-Kit+ cells, compared to their WT counterparts (Fig. 5b). We then conducted immunofluorescence staining on primary c-Kit+ cells treated with G-CSF for 2 h from both *Prtn3*^*−/−*^ and WT mice to examine the nuclear translocation of STAT3, the results revealed a significant increase in the nuclear translocation of STAT3 in the primary *Prtn3*^*−/−*^ c-Kit+ cells compared to the WT cells (Fig. 5c). Next, we stimulated isolated primary c-Kit+ cells from both *Prtn3*^*−/−*^ and WT mice with G-CSF for 24 h to analyze downstream gene expression related to STAT3-dependent myeloid differentiation, specifically examining *P27*^*kpi1*^ and *C/Ebpα*. The results showed that *Prtn3*^*−/−*^ c-Kit+ cells treated with G-CSF exhibited higher levels of expression of *P27*^*kpi1*^ and *C/Ebpα* compared to WT c-Kit+ cells (Fig. 5d).

To investigate the role of PRTN3 in the G-CSF/STAT3-dependent mechanism during granulopoiesis and monopoiesis, we differentiated progenitor LSK cells (Lin-, Sca-1+, cKit+ cells) into myeloid cells using SCF and G-CSF. Within 3–4 days of cytokine stimulation, both granulocytic (CD11b + Ly6G+) and monocytic (CD11b + Ly6C+) cell populations developed successfully in this specific in this vitro cell culture system. (Fig. S13a, b). The sorted stem/progenitor LSK cells from both *Prtn3*^*−/−*^ and WT mice were cultured in an ex vivo myeloid culture system with the addition of SCF and G-CSF at different time points. The results demonstrated a significant increase in the proportion of granulocytic as well as monocytic cell types when comparing Prtn3 knockout samples to those derived from WT LSK counterparts within this particular myeloid culture setup. (Fig. 5e, Fig. S13c). These findings suggest that the absence of PRTN3 enhances myeloid differentiation by activating the STAT3-dependent signaling pathway.

### STAT3 inhibitor rescues PRTN3-mediated myeloid differentiation

**Fig. 6:**
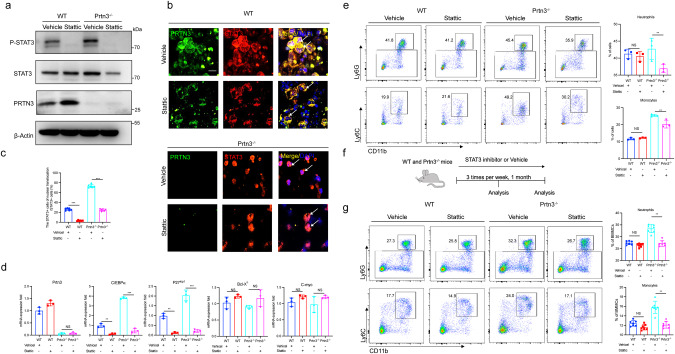
PTRN3-mediated myeloid differentiation was rescued by being treated with a STAT3 inhibitor. **a** Protein expression of STAT3, P-STAT3, PRTN3, and β-Actin were analyzed in LSK cells, which were isolated from *Prtn3*^*−/−*^ and WT mice, and treated with 10 nM Stattic for 24 h (*n* = 3); **b** immunofluorescence staining of STAT3 and PRTN3 were analyzed in LSK cells, which were isolated from *Prtn3*^*−/−*^ and WT mice, and treated with 10 nM Stattic for 24 h (*n* = 3); **c** Quantification of the number of cells with nuclear translocation from (**b**) (*n* = 7). **d** messenger RNA (mRNA) of *Prtn3*, *C/EBPɑ*, *p27*^*kip1*^, *Bcl-X*_*L*,_ and *C-myc* were determined in LSK cells, which were isolated from *Prtn3*^*−/−*^ and WT mice, and treated with 10 nM Stattic for 24 h (*n* = 3); **e** Representative flow cytometry plots for neutrophils and monocytes in LSK cells from WT and *Prtn3*^*−/−*^ mice. Numbers denote the frequency of each population among live singlets (*n* = 3). **f** The schematic diagram of WT and *Prtn3*^*−/−*^ mice with fed STAT3 inhibitor. **g** Representative flow cytometry plots for neutrophils and monocytes in LSK cells from WT and *Prtn3*^*−/−*^ mice. Numbers denote the frequency of each population among live singlets (*n* = 7). scale bars, 50 μm; *p < 0.05, **p < 0.01, ***p < 0.001. Data are the mean ± s.d.; *n*: biologically independent experiments. Statistical analysis was performed using an unpaired two-tailed Student’s *t* test.

To verify the involvement of the STAT3 pathway in the *Prtn3*-deficiency-induced myeloid differentiation, we performed a pharmacological study using Stattic, a potent inhibitor of STAT3, both in vitro and in vivo. Specifically, we treated *Prtn3*-deficient LSK cells with the small-molecule STAT3 inhibitor, which resulted in a significant reduction in STAT3 phosphorylation in both WT and *Prtn3*-deficient cells (Fig. 6a). The immunofluorescence staining of STAT3 and PRTN3 indicated that the upregulation of STAT3 nuclear translocation was reversed in the PRTN3-deficient LSK cells in comparison to the WT LSK cells (Fig. 6b, c). This observation was further confirmed by analyzing the downstream gene expression of STAT3 in the LSK cells through qPCR, which included *C/EBPα*, *p27*^*kpi1*^, *Bcl-X*_*L*_, and *C-myc* (Fig. 6d). To further verify the role of STAT3 in PRTN3-mediated myeloid differentiation, we used flow cytometry to analyze the proportion of neutrophils and monocytes, which revealed a significant increase in both neutrophils and monocytes in *Prtn3*^*−/−*^ LSK cells compared to WT LSK cells. Furthermore, we found that Stattic effectively reversed the myeloid differentiation induced by *Prtn3* depletion in LSK cells (Fig. 6e). We then administered Stattic (2 mg/kg) or a vehicle control orally via gavage three times per week for four weeks and analyzed the percentage of myeloid cells every two weeks to assess the potential rescue effects of Stattic on myeloid differentiation in mice with Prtn3 deficiency (Fig. 6f). Our results showed that administering Stattic every two weeks significantly ameliorated the elevated levels of myeloid cells in the peripheral blood of *Prtn3*^*−/−*^ mice (Fig. S14). The analysis of myeloid cells in bone marrow using flow cytometry indicated that the accumulation of myeloid cells induced by *Prtn3* deficiency was significantly reduced by Stattic treatment (Fig. 6g). In conclusion, these findings provide compelling evidence that Stattic effectively mitigates myeloid differentiation resulting from *Prtn3* deficiency.

### PRTN3 deficiency restarts the maturation and differentiation of leukemic blast

**Fig. 7:**
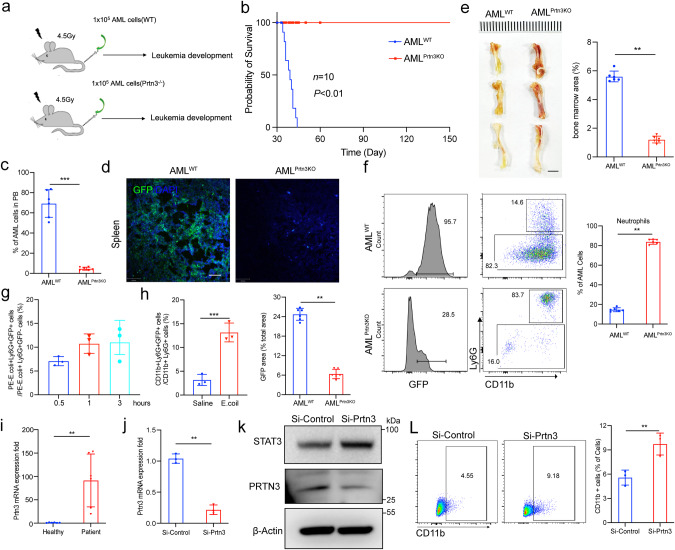
PTRN3 deficiency restarts the maturation and differentiation of leukemic blast. **a** The experimental design. **b** Survival curves of AML recipients with WT or *Prtn3*^*−/−*^ AML cells (*n* = 10 per group). **c** histogram analysis of the percentage of GFP+ leukemia cells in the peripheral blood (PB) of recipients 30 days after transplantation (*n* = 6). **d** immunofluorescence staining assay shows GFP+ leukemia cells in the spleen of recipients 30 days after transplantation (*n* = 6). **e** The bone marrow color (left) and histogram (right) of recipient on 30 days after transplantation (*n* = 6). **f** Flow cytometry (left) and histogram (right) analysis show the percentages of neutrophils from AML cells (GFP+) in the BM of recipients (*n* = 6). **g** Histogram analysis shows the percentages of GFP+ neutrophils with PE-E.coli uptake from BM of AML^Prtn3KO^ recipients at different time points (*n* = 3). **h** Histogram analysis shows the percentages of GFP+ neutrophils capable of chemotaxis migration in the abdominal cavity of AML^Prtn3KO^ recipients with E. coli injection for 6 h (*n* = 3). **i** messenger RNA of *Prtn3* expression in healthy peoples and patients (*n* = 6). **j**, **k** messenger RNA and protein expression of PRTN3 and β-Actin were analyzed in HL60 cells with Si-control or Si-*Prtn3* treatment for 24 h (*n* = 3). **l** Flow cytometry (left) and histogram (right) analysis of the percentage of CD11b+ leukemia cells in the HL60 cells with Si-control or Si-*Prtn3* treatment for 7 days (*n* = 3). *p < 0.05, **p < 0.01, ***p < 0.001. Data are the mean ± s.d.; *n*: biologically independent experiments. Statistical analysis was performed using an unpaired two-tailed Student’s *t* test.

To translate our findings into a clinically relevant setting, we evaluated the potential therapeutic benefits of reducing PRTN3 levels in acute myeloid leukemia. We extracted LSK cells from both WT and *Prtn3*^*−/−*^ mice and induced acute myeloid leukemia using MLL-AF9, then injected 1 × 10^5^ AML cells into lethally irradiated hosts and monitored their survival rates to determine the roles of *Prtn3* deficiency in AML progression (Fig. 7a). The administration of *Prtn3*-knockout AML significantly extended survival up to five months in mice (Fig. 7b). To further investigate the efficacy of this treatment, we examined leukemia cells in the peripheral blood of recipient mice four weeks after injection, showing a significant reduction in the percentage of green fluorescent protein-positive (GFP+) AML cells in *Prtn3*-knockout AML mice (Fig. 7c, Fig. S15a). The spleens and livers of *Prtn*3-knockout AML mice were notably smaller than those of control recipients (Fig. S15b, c). This observation was further supported by immunofluorescent staining (Fig. 7d, Fig. S15d), which indicated reduced infiltration in these organs. The bone marrow of recipients with *Prtn3*-knockout AML was visibly redder than that of control mice (Fig. 7e). The recipients with *Prtn3*-knockout AML exhibited an increase in the overall count of neutrophils and a decrease in the total number of monocytes, compared to the control recipients (Fig. S15e). Notably, the number of neutrophils with GFP+ was significantly higher in the recipients with *Prtn3*-knockout AML than those with WT AML (Fig. 7f, Fig. S15f). To investigate whether the *Prtn3*-knockout induces the differentiation of neutrophils with biology function, we evaluated the ability of phagocytosis and chemotaxis for neutrophil-like cells. The results showed that GFP+ neutrophils are capable of eliminating pathogens and can migrate to the abdominal cavity via chemotaxis (Fig. 7g, h, Fig. S15g, h), indicating that AML cells with *Prtn3* deficiency may differentiate into mature functional neutrophils.

Next, we examined the mRNA expression of *Prtn3* in the AML cells of patients and found a significantly increased mRNA level of *Prtn3* in the AML cells of patients compared with healthy controls (Fig. 7i). We conducted further evaluations to determine whether depletion of *Prtn3* enhances myeloid differentiation in human AML cell lines. We reduced the level of PRTN3 in HL60 cells using siRNA, which was confirmed by immunoblotting and qPCR (Fig. 7j, k). Our findings indicate that the STAT3 protein increased in the cells treated with Si-*Prtn3*, and there was an increase in the nuclear translocation of STAT3 in *Prtn3*-deficient cells (Fig. S16a). Moreover, the mRNA expression of downstream genes of STAT3, such as *C/Ebpɑ* and *p27*^*kip1*^, was elevated in *Prtn3*-deficient cells compared to control cells (Fig. S16b). We analyzed differentiated myeloid cells using flow cytometry and found a significant increase in CD11b+ cells in *Prtn3*-deficient cells compared to control cells, while CD14+ and CD15+ cells remained relatively unchanged (Fig. 7l, Fig. S16c). Additionally, the loss of *Prtn3* cells showed an increase in granulocyte-like cells, as observed through Wright-Giemsa staining (Fig. S16d). In NB4 cells, treatment with *Prtn3*-siRNA led to a decrease in PRTN3 protein levels, resulting in higher nuclear translocation and upregulation of downstream genes, as confirmed by immunoblotting, immunofluorescence staining, and qPCR (Fig. S17 a–c). However, we observed that the loss of *Prtn3* in NB4 cells did not result in the presence of CD11b+, CD14+, CD15+ cells, or granulocyte-like cells compared to control NB4 cells (Fig. S17 d, e), as detected by flow cytometry and Wright-Giemsa staining. Our findings indicate that inducing myeloid differentiation by decreasing PRTN3 is critical for AML-M2 rather than AML-M3. Furthermore, data mining on the publicly available website Bloodspot (https://servers.binf.ku.dk/bloodspot) revealed that *Prtn3* expression was dramatically elevated in most types of AML cells compared to healthy bone marrow (Fig. S17 f). We analyzed the correlation between *Prtn3* expression and overall survival in AML patients, as well as in different AML subtypes, utilizing the TCGA dataset (https://portal.gdc.cancer.gov/projects) and the Kaplan-Meier Plotter (https://kmplot.com/analysis/index.php?p=service). The findings indicate a significant difference in *Prtn3* expression among AML patients based on FAB classification (*P* < 0.05). Notably, AML patients with low *Prtn3* expression and an intermediate karyotype demonstrated a higher survival probability across various subtypes, including M1, M2, M3, and M5 (*P* < 0.05) (Fig. S18). The results of our in vivo and in vitro studies, conducted on both mice and humans, suggest that targeting PRTN3 could be a promising therapeutic approach for treating AML in a clinical setting.

## Discussion

**Fig. 8:**
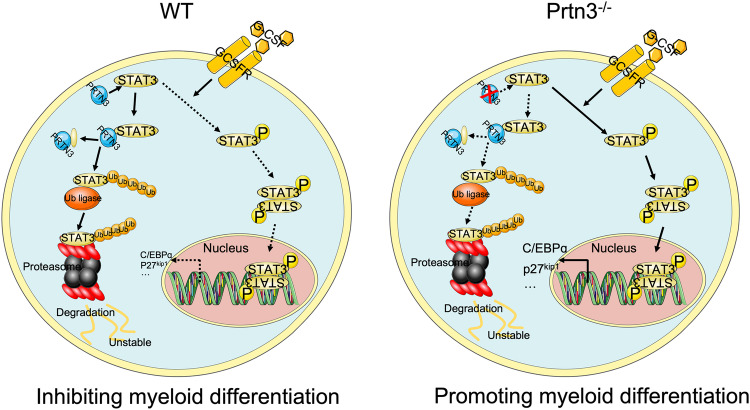
Schematic overview of events that lead to myeloid differentiation attributed to *Prtn3* deficiency. In WT mice, the level of G-CSF-induced STAT3 in LSK cells is counterbalanced by the PRTN3-dependent ubiquitin-mediated degradation of STAT3. In *prtn3* knockout (KO) mice, G-CSF-induced a sustained STAT3 phosphorylation and nuclear translocation that promotes robust expression of differentiation genes, including *C/Ebpα* and *P27*^*kpi1*^, that stimulates myeloid differentiation to promote an increased.

In this study, we have demonstrated the role of PRTN3 in the regulation of myeloid differentiation. Mechanistically, PRTN3 directly interacts with the N-terminal domain of STAT3, identifying cleavage sites, and thereby regulating STAT3 degradation through ubiquitination. Consequently, it inhibits the expression of STAT3-dependent genes (Fig. 8). Altogether, this study reveals a novel role for PRTN3 in regulating myeloid differentiation and suggests its potential as a target for treating acute myeloid leukemia.

Numerous regulators of STAT3 have been well-established, including cytokine receptors [18], receptor tyrosine kinases [19], G-protein-coupled receptors [20, 21], toll-like receptors [21–23], tyrosine phosphatases [24, 25], and the SOCS protein family [26, 27]. Our findings indicate that alterations in PRTN3 expression do not affect *Stat3* mRNA levels. Instead, PRTN3 exerts post-transcriptional control by regulating STAT3 degradation, suggesting its role as a novel regulator of STAT3 at the post-transcriptional level. Furthermore, the previous association between the PRTN3 protein and STAT3 is substantiated by our data [28], confirming the direct interaction between PRTN3 and STAT3. Mass spectrum and CO-IP analyses demonstrate the specificity of this interaction with STAT3, distinguishing it from STAT1, STAT2, and STAT5,indicating that STAT3 is a specific target of PRTN3 in signal transducer and activator of the transcription family.

The STAT3 signal plays a central role in inducing myeloid differentiation in response to growth factors [29–32]. Our findings provide substantiation for the role of PRTN3 in identifying cleavage sites on STAT3, resulting in ubiquitin-mediated degradation and subsequent reduction in STAT3-dependent gene expression. This connection between PRTN3 and myeloid differentiation is particularly evident in G-CSF-stimulated LSK cells. Our data suggest that PRTN3 is a viable candidate for targeted regulation of myeloid differentiation, potentially avoiding side effects associated with direct manipulation of STAT3.

Neutrophil serine proteases, such as neutrophil elastase (NE), cathepsin G (CG), and PRTN3, have been implicated in the regulation of inflammatory conditions [33]. Our investigation reveals that PRTN3 displays specificity for STAT3 compared to NE and CG, suggesting the need for further studies regarding the functions of NE and CG within the signal transducer and activator of the transcription family. Furthermore, PRTN3 is expressed in hematopoietic processes, encompassing hematopoietic stem and progenitor cells [9] as well as mature myeloid cells [8, 10, 34, 35]. Previous reports have indicated that PRTN3 regulates neutrophil apoptosis during inflammatory responses through the targeting of pro-caspase-3 [8] and modulates the HSPC population through apoptosis [9]. Our findings suggested that further studies are warranted to investigate the role of PRTN3 in diverse hematopoietic cell types, which will enhance our understanding of protease functions.

It has been reported that the downregulation of PRTN3 expression in response to treatment with All-trans retinoic acid (ATRA) plus arsenic trioxide (ATO), leads to the differentiation of leukemia cells capable of ROS production and phagocytosis [36, 37]. While previous studies did not investigate the underlying mechanism [36], our findings suggest that PRTN3 regulates STAT3-dependent myeloid differentiation in the context of acute myeloid leukemia (AML). STAT3 serves as a pivotal molecule in anti-AML immunity, particularly through T cells. Our findings unequivocally show that the depletion of PRTN3 has no impact on T cell functions, encompassing proliferation, activation, and anti-AML immunity, strongly suggesting that PRTN3 controls AML differentiation rather than influences T cell immunity in AML. The extent of differentiation varies across AML subtypes, with AML M2 displaying the most notable response to PRTN3 deregulation in cell lines. Intriguingly, AML patients with low *Prtn3* expression exhibit a higher probability of survival across various subtypes, including M1, M2, M3, and M5, indicating that inhibiting PRTN3 could potentially lead to a substantial improvement in the clinical survival of AML patients. Significantly, our data also reveal that targeting PRTN3 inhibition promotes the differentiation of AML cells into neutrophils capable of phagocytosis and chemotaxis migration, hinders malignant proliferation, induces cellular maturation, significantly enhances survival rates, and reduces AML cell counts in mice with MLL-AF9-induced AML, underscoring PRTN3 as a promising therapeutic target for AML. However, although targeting PRTN3 holds promise as a novel therapeutic approach for AML, direct targeting with small molecules faces challenges. Thus, further investigations, encompassing both fundamental and clinical studies, are warranted to evaluate the therapeutic potential of inhibiting PRTN3 for the treatment of AML.

## Materials and methods

### Mice

*Prtn3*^*−/−*^ mice were generated by GemPharmatech Inc (Jiangsu, China) using CRISPR/Cas9 technology, as described previously [38]. Tails were got from offspring, and the primers used for *Prtn3* genotyping were forward: 5′- CCCTGATCCACCCGAGATTC-3′ and reverse 5′- GGTTCTCCTCGGGGTTGTAA -3′. *Prtn3*^*flo/flox*^ mice (Strain #:030761) were obtained from Jackson Laboratories. LysMcre mice (Strain #:004781) were purchased from Jackson Laboratories. The myeloid lineage conditional *Prtn3* knockout mice (*Prtn3*^*LyzKO*^) were crossed by LysMcre mice and *Prtn3*^*flo/flox*^ mice. The primers used for *Prtn3*^*flox/+*^ genotyping were forward: 5′- GGT CTG AAC TGA CAG CAA AGC-3′ and reverse 5′- CCC TAA CCA CTC CCC TAT CC-3′. the primers used for LysMcre genotyping were common: 5′- AAG GAG GGA CTT GGA GGA TG -3′, wild type reverse 5′- GTC ACT CAC TGC TCC CCT GT -3′, Mutant Reverse 5′- ACC GGT AAT GCA GGC AAA T -3′. Six-to-eight-week-old C57BL/6 CD45.2 mice and CD45.1 mice were purchased from Beijing Vital River Laboratories. In all experiments with knockout mice, corresponding littermates as WT controls were used. All animals were maintained at the Animal Core Facility of the State Key Laboratory of Experimental Hematology.

### The mouse model of hematopoietic stem cell transplantation

Mice carrying CD45.1 (Recipient) were irradiated with single doses of 9.5 Gy. For hematopoietic stem cell total transplantation, recipients were injected with 1 × 10^3^ sorted LSK cells from the donor (CD45.2) and 5 × 10^5^ BM cells from the donor (CD45.1). Transplanted into recipient mice after myelopoiesis by tail vein injection. Mice are given oral enrofloxacin for 4 weeks after transplantation. Mice are Sacrificed 4 months after transplantation, the femur, tibia, ilium, and spleen were harvested, and bone marrow cells are flushed out with PBE by 1 mL syringe, filtered, and resuspended. Lysis of red blood cells of the bone marrow with erythrocyte lysate, the chimerism was analyzed at the end of each experiment. Flow cytometry antibody [CD45.1-PE (Biolegend, 110708), CD45.2-APC (Biolegend, 109814)] labeling is performed and finally analyzed by flow cytometry. At the same time, bone marrow and liver frozen sections with a thickness of 7 μm were prepared, corresponding immunofluorescence staining was performed, and the ratio of CD45.1 to CD45.2 was observed by confocal microscope.

### Generated AML mouse model

For transplantation of *Prtn3*^*−/−*^ leukemia cells, 2 × 10^5^ MLL-AF9 transduced lineage cells from WT or *Prtn3*^*−/−*^ mice were injected intravenously into half-lethally irradiated (4.5 Gy) C57BL/6 recipient mice. Then, WT or *Prtn3*^*−/−*^ AML cells were further sorted from primary recipient mice, and then, the cells were transplanted with 2 × 10^5^ BM into half-lethally irradiated (4.5 Gy) C57BL/6 recipient mice.

### STAT3 inhibitor mouse model

Mice aged at 8 weeks were randomized to receive either Stattic (2 mg/kg, p.o. #T6308, TargetMol, USA) or vehicle alone (5%DMSO, 95% peanut oil) for 4 weeks. 6 mice in each group were sufficient for our experiments.

### Mouse model of *E. coli*-induced peritonitis for testing chemotaxis migration

E. coli cultures were grown in Luria-Bertani (LB) liquid media overnight and reached the exponential growth phase (0.5 OD600) [39]. Then, washed three times with sterile normal saline. Then AML^Prtn3KO^ mice were injected intraperitoneally (i.p.) with 1 × 10^6^ *E. coli* in a total volume of 500 μl saline. Six hours after bacterial infection, mice were anesthetized. Peritoneal lavage was performed using 10 ml of normal PBS that contained 0.1% bovine serum albumin. The cells were incubated with antibodies and detected by flow cytometry.

### Cells

HEK293T was cultured in DMEM (ThermoFisher) supplemented with 10% (v/v) FBS (Hyclone, UK) at 37 °C. HEK293T cell lines were purchased from ATCC (Manassas, USA). HL-60 cells were cultured in IMDM medium (Gibco) supplemented with 2% (v/v) FBS (Hyclone, UK) at 37 °C. NB4 cells were cultured in 1640 medium (ThermoFisher) supplemented with 10% FBS (Hyclone, UK) at 37 °C. HL-60 cell lines and NB4 cell lines were gifted from Dr. Guoguang Zheng (Tianjin, China). All cells were grown at 37 °C in a 5% CO_2_ incubator (Thermo Fisher, USA). Cells were transfected with Lipofectamine RNAiMAX (Invitrogen, USA) was used for the transfection of plasmids or siRNAs into cells.

### Plasmid examination, cell culture, virus production, and transduction

An MSCV-MLL-AF9-IRES-GFP plasmid was gifted from Dr. Tao Cheng and Dr. Weiping Yuan at the State Key Laboratory of Experimental Hematology, National Clinical Research Center for Blood Diseases [40]. We re-clone the MLL fragment by qPCR (Fig S 17a). The mRNA was isolated from AML^Prnt3KO^ mice to determine two key AF9 genes, *Hoxa9* and *Meis1*, to confirm the plasmid by qPCR (Fig S 17b). The primer as follows: ALL-AF9 F: AAC CAC CTC CGG TCA ATA AGC; R: TTC ACG ATC TGC TGC AGA ATG; Hoxa9 F: GGA ATA GGA GGA AAA AAC AGA AGA GG; R: TGT ATG AAC CGC TCT GGT ATC CTT; Meis1: F: TCA CCA CGT TGA CAA CCT CG; R: GCT TTC TGC CAC TCC AGC TG.

An MSCV-MLL-AF9-IRES-GFP plasmid together with pKat and pVSVG packaging plasmids was transfected into 293T cells using Lipofectamine 3000 (ThermoFisher) to transform normal hematopoietic stem and progenitor cells into AML cells [41]. After 72 h of culture, the supernatant containing retroviruses was collected and concentrated BY an Amicon filter (Millipore). normal hematopoietic stem and progenitor cells from WT and *Prtn3*^*−/−*^ mice were enriched by lineage cell depletion beads (Miltenyi), and then transduced with MLL-AF9 retroviruses in the presence of 4 μg/ml polybrene (Sigma). The cells were incubated in IMDM (Gibco) with 15% FBS, 50 ng/ml mouse SCF (Peprotech), 10 ng/ml mouse IL-3 (Peprotech), and mouse IL-6 (Peprotech) for 2 days.

Prepare whole bone marrow single-cell suspension of experimental mice (donor, CD45.2), perform positive enrichment of c-kit magnetic beads, and sort hematopoietic stem cell LSK (Lin-c-Kit + Scal1+) cells. Sacrificed CD45.1 mouse and prepare a whole bone marrow single-cell suspension.

### Isolated primary human CD34+ HPCs

Primary CD34+ hematopoietic progenitor cells (HPCs) were isolated from the blood of the human umbilical cord obtained from Blood Diseases Hospital. In brief, using magnetic bead separation and viably frozen as previously described [42], red blood cells first were removed from total blood, and then hematopoietic cells were purified by Ficoll gradient centrifugation. CD34+ HPCs were purified using a Dynabeads™ CD34 progenitor cell selection system (Invitrogen™, 11301D), according to the manufacturer’s instructions. Frozen cells were thawed and recovered overnight in stem cell media or prepared for protein extraction.

### Isolated primary human AML cells

The human AML cell samples were obtained from Blood Diseases Hospital. In brief, using magnetic bead separation and viably frozen as previously described [42], red blood cells were removed from total blood, and then hematopoietic cells were purified by Ficoll gradient centrifugation.

### Bone marrow culture

Bone marrow cells were harvested from the tibia, femur, and pelvic bones by crushing the bones with a mortar and pestle in PBE [43]. Cells gathered were filtered through a 70 μm filter to make a single cell suspension and were enriched for progenitor cells using mouse hematopoietic progenitor cell isolation kit (BioLegend, San Diego, CA, USA) and cultured in DMEM media with 10% FBS. Cell culture media were supplemented with stem cell factor (SCF: 50 ng/ml); FLT3L (10 ng/ml); and thrombopoietin (TPO: 10 ng/ml) (BioLegend, San Diego, CA, USA).

### LSK expansion culture

Sorted LSK cells were cultured at a concentration of 10^4^ cells per 1 mL media supplemented with SCF (50 ng/ml) and TPO (50 ng/ml) in a single well of a 12-well tissue culture plate. The choice and concentration of cytokines were based on the stem cell expansion protocol as previously reported [43]. LSK expansion was a culture in Serum-free StemSpan SFEMII medium (STEMCELL Technologies, Vancouver, Canada), with flow analysis on the second, third, or fourth day post-LSK culture.

### Myeloid culture

Sorted LSK cells were cultured in DMEM media supplemented with SCF (50 ng/ml) and G-CSF (10 ng/ml) in a 48-well tissue culture plate. The choice and concentration of cytokines were based on the myeloid differentiation protocol as described [44]. DMEM media supplemented with 10% FBS was used for cell culture media. Cells were analyzed after the culture of LSK cells by flow cytometry.

### CFSE T cell proliferation assay

For the T cell proliferation assay, anti-CD3-preactivated T cells were first labeled with CFSE (Invitrogen; Cat No. C34554) in a dilution of 1:1000. After 3 days of co-culture, T cells were harvested and CFSE density was measured by flow cytometry. Each experiment was performed in triplicate.

### T cell activation bioassay

For T cell activation, the T cells from the spleen were labeled by CD62I (Biolegend, 104405), CD69 (Biolegend, 164203), IFN-γ (Biolegend, 505807), and TNF-α (Biolegend, 506305) for 30 min on ice, then measured by flow cytometry.

### T-cell killing assay

Mice spleen T cells were isolated and cultured in RPMI-1640 medium and activated with Dynabeads™ Mice T-Activator CD3/CD28 (Gibco, CA, USA) and 10 ng/ml IL-2 for 3 days according to the manufacturer’s instructions. AF9-AML cells were seeded into 12-well plates at a cell-dependent concentration. After 24 h, activated T cells were cocultured with AF9-AML cells for 24 h at a ratio of 10:1. Cell debris was removed, and cells were harvested and labeled with annexin V and PI for fluorescence-activated cell sorting (FACS) analysis.

### siRNA-mediated gene knockdown

human primary CD34+ cells, HL-60 cell line, and NB4 cells line were transfected with non-targeting control siRNAs (sc-44230, Santa Cruz), or *Prtn3* siRNA (sc-42968, Santa Cruz). 6 μL LipofectamineTM RNAiMAX (13778075, Thermo FisherScientific) was used as the transfection reagent based on the manufacturer’s protocols. Cells were harvested 48 h after transfection. The efficiency of *Prtn3* knockdown was analyzed by Western Blot.

### Complete blood count

The peripheral blood (50 μL) was collected by heparinized capillary tubes (Fisher Scientific) and transferred into K2-EDTA-coated tubes (Becton Dickinson). Blood parameters were analyzed by using Hemavet 950FS (Drew Scientific).

### Phagocytosis assay

Fluorescein conjugate E. coli (K-12 strain) BioParticles (Molecular Probes, E2861, PE-labeled heat-killed E. coli) were diluted in serum media and incubated for 30 min, followed by two washes in PBS. Next, PE-labeled, heat-killed E. coli was added to opti-MEM medium with 10% FCS and incubated with GFP+ cells from AML^Prtn3KO^ mice at different time points, 0, 0.5, 1, 3, 5 h in a humidified atmosphere of 5% CO_2_ at 37 °C, and GFP+ cells were washed twice with PBS, and then staining with Ly6G-APC antibody, followed by flow cytometric analysis [45].

### Plasmids

Human *Stat3* cDNA (RDC1454) was kindly obtained from Biotechne and cloned into the pcDNA3.1 vectors, respectively. Flag-tagged Stat3 (1-770), Flag-tagged Stat3 (1-688), Flag-tagged Stat3 (1-583), Flag-tagged Stat3 (1-465), Flag-tagged Stat3 (1-320), Flag-tagged Stat3 (1-130) were subcloned from pcDNA3.1-STAT3-Flag as described previously [46]. N-terminal domain or C-terminal domain *Prtn3* were amplified from the HA-tagged full-length *Prtn3*, which were then subcloned into the pcDNA3.1 vectors. All constructs were confirmed by DNA sequencing. HA-Ubiquitin plasmid (Plasmid #18712) was purchased from the addgene.

### Flow cytometry and cell sorting

BM cells from femurs and tibias were flushed into PBE supplemented with 2% FBS (Atlanta Biologicals, S11150H). Red blood cells were lysed by eBioscience™ 1X RBC Lysis Buffer (Invitrogen™, 00-4333-57). Ten million BM cells were incubated with an antibody mixture including antibodies for lineage markers: Ly-6C-FTIC (Biolegend, 128005), Ly-6G-PE-Cy7 (Biolegend, 127617), CD3-PE (Biolegend, 980008), CD11b-APC (Biolegend, 101212), CD45R/B220- PE-Cy5 (Biolegend, 103209), as well as Sca1-PE/Cy7 (Biolegend, 108114) and c-Kit-APC/Cy7 (Biolegend, 105826) in DMEM (Life Technologies, 31053-028) supplemented with 2% FBS. For LSK subset analysis, Lin-APC (Biolegend, 348703), Sca1-PE/Cy7 (Biolegend, 108114) and c-Kit-APC/Cy7 (Biolegend, 105826). CD34-FITC (eBioscience, 11-0341-85) and CD135-PE (Biolegend, 135306) were used. For LK subsets, CD34-FITC and CD16/32-PE (Biolegend, 101308) were used. Samples were incubated on ice for 30 min, then washed and filtered before analysis. Samples were stained for one hour on ice for CD34 staining. Data were collected on FACSCanto II or LSR II flow cytometers (Becton Dickinson) and analyzed by FlowJo software (Tree Star).

For sorting highly purified cells, an immunomagnetic negative selection kit was used to enrich HSPCs (Stem Cell Technologies, 19756). Enriched cells were stained with Lin-APC (Biolegend, 348703), c-Kit-PE (Biolegend, 105808), and Sca1- PE/Cy7 (Biolegend, 108114). DAPI (BD Biosciences, 564907) was added to exclude dead cells. Cells were sorted on an Aria (Becton Dickinson) or MoFlo (Dako) cell sorter.

### Protein preparation, immunoprecipitation, and immunoblotting analysis

For immunoprecipitation and ubiquitination analysis, the total cellular protein was isolated with a cell lysis buffer (Cell Signaling Technology; #9803). Cell lysates were incubated with the appropriate primary antibody [anti-HA (Invitrogen, 26183), anti-FLAG (Invitrogen; 26183), anti-STAT3(Cell Signaling Technology, 9139), anti-PRTN3 (Abcam, ab103632)] overnight at 4 °C followed by 1.5 h incubation with protein A/G agarose beads. The beads were washed three times with the lysis buffer and were eluted in 5 × SDS/PAGE loading buffer for immunoblotting. For immunoblotting analysis, cells were lysed with cell lysis buffer supplemented with protein inhibitors (Roche, Switzerland). The cell lysates were incubated with the following antibodies: anti-HA (Invitrogen, 26183), anti-FLAG (Invitrogen; 26183), anti-STAT1(Cell Signaling Technology, 9172), anti-STAT2 (Cell Signaling Technology, 4597), anti-STAT3 (Cell Signaling Technology, 9139), anti-STAT5 (Cell Signaling Technology, 94205), anti-PRTN3 (Abcam, ab103632), anti-Phospho-STAT3 (Cell Signaling Technology, 9145), anti-Neutrophil Elastase Antibody (Cell Signaling Technology, 63610), anti-Cathepsin G (Abcam, ab192793) and anti-β-Actin (Cell Signaling Technology, 4967), overnight at 4 °C. After washing with TBST three times, the membranes were incubated with second antibody (Goat Anti-Mouse IgG (H + L)-HRP Conjugate; 1706516; Bio-Rad; Goat Anti-Mouse IgG (H + L)-HRP Conjugate, 1706516; Bio-Rad) (dilution at 1:10,000) at room temperature for 90 min. Images were visualized using an ChemiDoc (Bio-Rad). For quantification of the Immunoblotting assay, densitometric analysis of bands of target proteins and loading controls (β-Actin) was performed with NIH Image J software as we described previously [47]. Western blotting was performed in three independent experiments.

### Recombinant protein preparation

The STAT3 recombinant protein (Ab43618) was ordered from Abcam. The STAT3 recombinant protein (ST3-H5149-100 μg) was ordered from ACRO. The STAT3 recombinant protein (HY-P70574) was ordered from ACE. The PRTN3 recombinant protein (HY-P70509) was ordered from ACE. All proteins were prepared for surface plasmon resonance (SPR) measurement.

### Immunofluorescence (IF) staining and Wright-Giemsa staining

Formaldehyde-fixed, paraffin-embedded liver and bone samples were sectioned into 7 μm slides. immunofluorescence (IF) staining was performed as previously described [48]. Briefly, formaldehyde-fixed, OCT-embedded slides for tissue samples were subjected to antigen retrieval, followed by blocking and antibody (anti-STAT3 (Cell Signaling Technology, 9139), and anti-PRTN3 (Abcam, ab103632)) (1:100) incubation overnight. For in vitro experiments, cells were seeded on coverslips and were fixed with 4% paraformaldehyde, permeated with 0.1% Triton X- 100, and then blocked with 10% BSA. Primary antibodies (anti-STAT3 (Cell Signaling Technology, 9139), and anti-PRTN3 (Abcam, ab103632)) (1:100) were diluted as suggested and incubated at 4 °C overnight in a moist chamber. The slides were then washed with PBS and incubated with 488-/546 -conjugated secondary antibodies. Slides were further incubated with DAPI (Invitrogen) and mounted in IF mounting medium (Service Bio). Quantification of the mean fluorescence intensity was performed with the ImageJ software under at least three randomly selected fields. Confocal microscopy was performed using a Spinning disk confocal microscopy system (Perkinelmer, ultraVIEW VOX).

For Wright-Giemsa staining, Cells (2 × 10^5^ cells/mL) were prepared. Then, cells were harvested and the density was adjusted to 2 × 10^4^ cells/mL. The cells were collected and used Wright–Giemsa (Leagene, Beijing, China) staining according to the manufacturer’s protocol on slides prepared by cytospin to perform a morphological assessment. The morphology of cells was observed under a light microscope.

### Colony-forming cell assays

Colony formation assays were performed as previously reported [9]. LSK cells (2 × 10^4^) from WT and knockout mice were seeded in semisolid Methocult GF M3434 medium containing SCF, IL-3, IL-6, and Epo for detection of colony-forming units-granulocyte, monocyte, and burst-forming units-erythroid (Stem Cell Technologies, 03434). Colony numbers were counted on day 7, and images for colony sizes were obtained on day 8.

### Analysis of the correlation between *Prtn3* and patient survival in the TCGA database

An online tool Kmplot (https://kmplot.com/analysis/index.php?p=service) was used to determine the correlation between *Prtn3* and patient survival, as well as the *Prtn3* level from patients with M0-M7 was collected from the TCGA database (https://portal.gdc.cancer.gov/projects).

### Surface plasmon resonance (SPR) measurement

To measure the binding affinities of PRTN3 with the STAT3, The N-terminal domain of STAT3, and STAT3 without the N-terminal domain, an SPR-based Biacore K8 biosensor (Biacore AB, Uppsala, Sweden) with CM5 was used as the sensor chip as previously reported [49]. The target proteins were diluted to a final concentration of 20 μg/mL in 10 mmol in PBS (pH 7.2) and then immobilized to CM5 by the standard primary amine coupling method at 25 °C. Firstly, The CM5 surface was activated by injecting a 1:1 mixture containing 200 mmol/L 1-ethyl-3-(3-dimethylamino propyl) carbodiimide hydrochloride and 50 mmol/L N-hydroxysuccinimide for a target level of 10,000 RU at a flow rate of 20 μL/min. The target proteins were injected into the CM5 surface. All the screening assays were performed over the unmodified dextran surface and the protein surface. Each sample assay consisted of a 180 s buffer injection and a 300 s dissociation phase and the blank injection was used to check the carryover effects. The signal was adjusted for nonspecific binding of the samples to the dextran matrix by subtracting the signal in the reference channel from the signal in the active channel. The experimental data were fitted and analyzed using the BIA evaluation software (Cytiva, Sweden) by steady-state analysis.

### Electrophoresis

A STAT3 (1 μM final) was incubated with hPR3 (1 μM final) for 30–120 min at 37 °C in PBS solution (pH 7.2). The mixtures were then denatured/reduced by the addition of reducing buffer (19, b-mercaptoethanol) and then boiled for 5 min at 95 °C. The mixtures were separated on a 10% SDS-PAGE gel at room temperature for about 1 h and visualized by silver nitrate (Invitrogen, Carlsbad, CA, USA) staining according to the manufacturer’s protocol. Size markers (25–170 kDa) were used (Thermo Fisher Scientific, Waltham, MA, USA). The bands representing the proteins according to their size appeared dark brown on the gel.

### Molecular docking

As previously reported [50], The in-silico analysis of the protein-ligand binding mode was performed with the crystal structure of hPR3 used as a receptor (1FUJ.pdb) [51]. The models of ligand structure were prepared and optimized using ChemBio3D 12.0. The protonation and atom types of all molecules were set with SPORES.

### Mass spectrometry

Mass spectrometry was performed as previously described [52]. Briefly, harvested cell lysates were incubated with the anti-HA primary antibodies overnight at 4 °C, and conjugated with protein A/G beads (Santa Cruz Biotechnology, CA) for 1.5 h. After washing, immunoprecipitants were boiled in Laemmli sample buffer for 5 min. The immunoprecipitated proteins were detected by reverse phase liquid chromatography/mass spectrometry (RPLC/MS)-ESI-Q-ToFQ analyzer (TripleTOF 6600 MS system, Applied Biosystem, USA).

### MALDI-TOF MS

STAT3 samples were analyzed on an UltraFlex I mass spectrometer (Bruker Daltonics). A cysteine reduction was carried out for 10 min at 37 °C using 5 mM Tris(2-carboxyethyl) phosphine hydrochloride prior to MS analysis of whole STAT3 samples. Samples were diluted 50-fold or 100-fold in a solution of 4HCCA saturated in a solution of 66.6% water, 33.3% acetonitrile, and 0.1% trifluoroacetic acid. Matrix/sample solutions were spotted onto a gold-plated sample probe using the ultrathin layer method [50]. MALDI-TOF- MS spectra were processed and annotated using FLEXANALY- SIS 3.3 (Bruker Daltonics) and PAWS 8.5 (ProteoMetrics) software, respectively.

### Quantitative RT-PCR

Total RNA was prepared from neutrophils, LSK cells, HL60 and NB4 cells using TRIzol (Invitrogen). cDNA was generated using 1 μg total RNA using a High-Capacity cDNA Reverse Transcription Kit from Applied Biosystems. Quantitative polymerase chain reaction (qPCR) was performed on the CFX-96 real-time PCR detection system (Bio-Rad, Hercules, CA, USA) with iQ SYBR Green Supermix (1708880B10, Bio-Rad) under the following conditions: 94 °C for 10 min; 40 cycles of 94 °C for 15 s, 58 °C for 30 s, 72 °C for 30 s; and final elongation at 72 °C for 15 min. Relative mRNA expression to that of the housekeeping gene β-Actin was calculated. Data were normalized and the control group was set at 1.0. Gene expression was then measured with a CFX96 Real-Time PCR System (Bio-Rad) using the following primer pairs: Prtn3(Human): F 5ʹ- CCTGCAGGAGCTCAATGT-3ʹ; R 5ʹ- CTG AGT CTC CGA AGC AGA TG-3ʹ ; Prtn3(Ms): F 5ʹ- CTT GAT CTG CAA TGG CAT TCT T-3ʹ; R 5ʹ- GGC GAA GAA ATC AGG GAA CT-3ʹ ; Stat3 (Human): F 5ʹ- GAG AAG GAC ATC AGC GGT AAG-3ʹ; R 5ʹ- CAG TGG AGA CAC CAG GAT ATT G-3ʹ; C/EBPɑ (Human): F 5ʹ- GAA GTC GGT GGA CAAG AAC A-3ʹ; R 5ʹ- TCA TTG TCA CTG GTC AGC TC -3ʹ; C/EBPɑ(Ms): F 5ʹ- CAA GAA GTC GGT GGA CAA GAA-3ʹ; R 5ʹ- CGT TGC GTT GTT TGG CTT TA-3ʹ ; P27^kpi1^(Human): F 5ʹ- CTA ACTC TGA GGA CAC GCA TTT-3ʹ; R 5ʹ-TGC AGG TCG CTT CCT TAT TC-3ʹ; P27^kpi1^(Ms): F 5ʹ- AGC TTG CCC GAG TTC TAC TA -3ʹ; R 5ʹ- GAG TTT GCC TGA GAC CCA ATT A -3ʹ; BcL-XL(Human): F 5ʹ- GGT GGT TGA CTT TCT CTC CTA C -3ʹ; R 5ʹ- TCT CCG ATT CAG TCC CTT CT-3ʹ; BcL-XL(Ms): F 5ʹ- TGG TCG ACT TTC TCT CCT ACA-3ʹ; R 5ʹ- CCC TCT CTG CTT CAG TTT CTT C-3ʹ; C-myc (Human)F 5ʹ- CTG AGG AGG AAC AAG AAG ATG AG -3ʹ; R 5ʹ- TGT GAG GAG GTT TGC TGT G-3ʹ; C-myc(Ms) F 5ʹ- CTC CGT ACA GCC CTA TTT CAT C -3ʹ; R 5ʹ- TGG GAA GCA GCT CGA ATT T-3ʹ; β-Actin (Ms): F 5ʹ-AGC CAT GTA CGT AGC CAT CCA-3’; R 5ʹ- TCT CCG GAG TCC ATC ACA ATG-3ʹ; Gapdh (Human): F 5ʹ- AGG GCT GCT TTT AAC TCT GGT-3ʹ; R 5ʹ-CCC CAC TTG ATT TTG GAG GGA-3ʹ.

### Statistical analysis

Each experiment was performed at least three times. All experiment data were analyzed using GraphPad Prism 9.0 (GraphPad Software Inc. USA) and were presented as the mean ± SD. Statistical analysis was performed using Student’s *t* test, one-way ANOVA, or two-way ANOVA. A value of *P* < 0.05 was considered statistically significant.

### Supplementary information

Supplementary Figure

Original Data File

## Data Availability

Detailed information on antibodies and reagents used in the manuscript is provided in the Materials and Methods. Raw western blots are available in the Supplemental file.
