# Supplementary material for: Proteinase 3 depletion attenuates leukemia by promoting myeloid differentiation
Source: Cell Death Differ. 2024 Apr 8;31(6):697–710. doi: 10.1038/s41418-024-01288-4 (PMC11165011; doi:10.1038/s41418-024-01288-4)
Supplement: Supplementary file 1 — Supplementary Figure [file 41418_2024_1288_MOESM1_ESM.docx]

**Proteinase 3 depletion attenuates leukemia by promoting myeloid differentiation**

Huan Liu^1,2,a^, Lu Sun^1,2,a^, Hongfei Zhao^1,2^, Zihan Zhao^1,2^, Shiyue Zhang^1,2^, Shan Jiang^1,2,3^, Tianran Cheng^1,2^, Xiaohan Wang^4^, Tong Wang^1,2^, Ya Shao^1,2^, Haiyan Zhu^5^, Huijuan Han^6^, Yigeng Cao^1,2,7^, Erlie Jiang^1,2,7^, Yihai Cao^8,#^, Yuanfu Xu^1,2,#^

^1^State Key Laboratory of Experimental Hematology, National Clinical Research Center for Blood Diseases, Haihe Laboratory of Cell Ecosystem, Institute of Hematology & Blood Diseases Hospital, Chinese Academy of Medical Sciences & Peking Union Medical College, Tianjin, China, 300020.

^2^Tianjin Institutes of Health Science, Tianjin, China, 301600.

^3^Clinical Laboratory, The First Affiliated Hospital of Xi'an Jiaotong University, Xi'an, China, 710061.

^4^The Second School of Clinical Medicine, Guangdong Medical University, Dongguan, China, 523808.

^5^Department of Clinical Lab, Weihai Municipal Hospital, Weihai, China, 264200.

^6^Department of Medical Laboratory, School of Clinical Medicine, Ningxia Medical University; Ningxia Key Laboratory of Clinical and Pathogenic Microbiology, General Hospital of Ningxia Medical University, Yinchuan, China, 750001.

^7^Hematopoietic Stem Cell Transplantation Center, Institute of Hematology and Blood Diseases Hospital, Chinese Academy of Medical Sciences and Peking Union Medical College, Tianjin, China, 300020.

^8^Department of Microbiology, Tumor and Cell Biology, Karolinska Institute, Solna, Sweden, 17165.

^a^Huan Liu and Lu Sun contributed equally to this study.

**Short Title:** PRTN3 inhibits myeloid differentiation

^#^ **To whom correspondence should be addressed:**

**Dr. Yihai Cao,**

Department of Microbiology, Tumor and Cell Biology, Karolinska Institute, Solna, Sweden, 17165. E-mails: yihai.cao@ki.se

**Dr. Yuanfu Xu**,

State Key Laboratory of Experimental Hematology, Institute of Hematology and Blood Diseases Hospital, Chinese Academy of Medical Sciences and Peking Union Medical College, 288 Nanjing Road, Tianjin, China, 300020, E-mails: [xuyf@ihcams.ac.cn](mailto:xuyf@ihcams.ac.cn)

**Supplementary Figure**


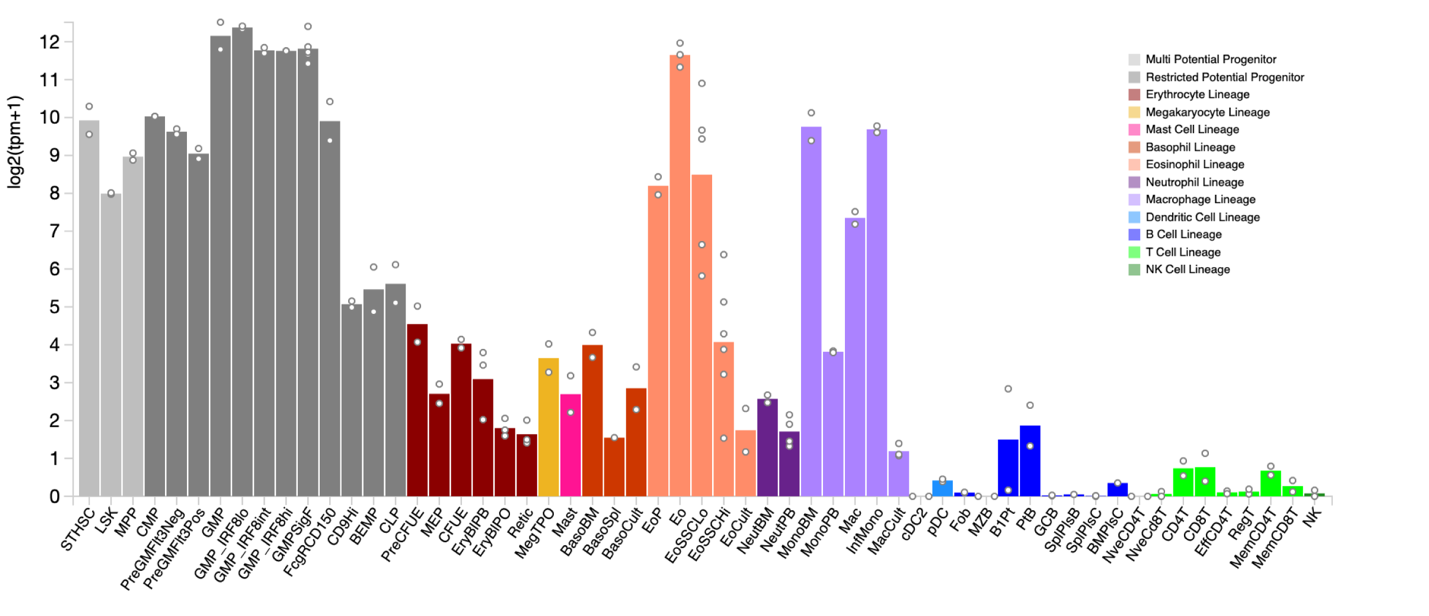


**Figure S1 The PRTN3 expression in hematopoiesis.** Data mining in the Haemopedia RNA-seq data sets (https://www.haemosphere.org)


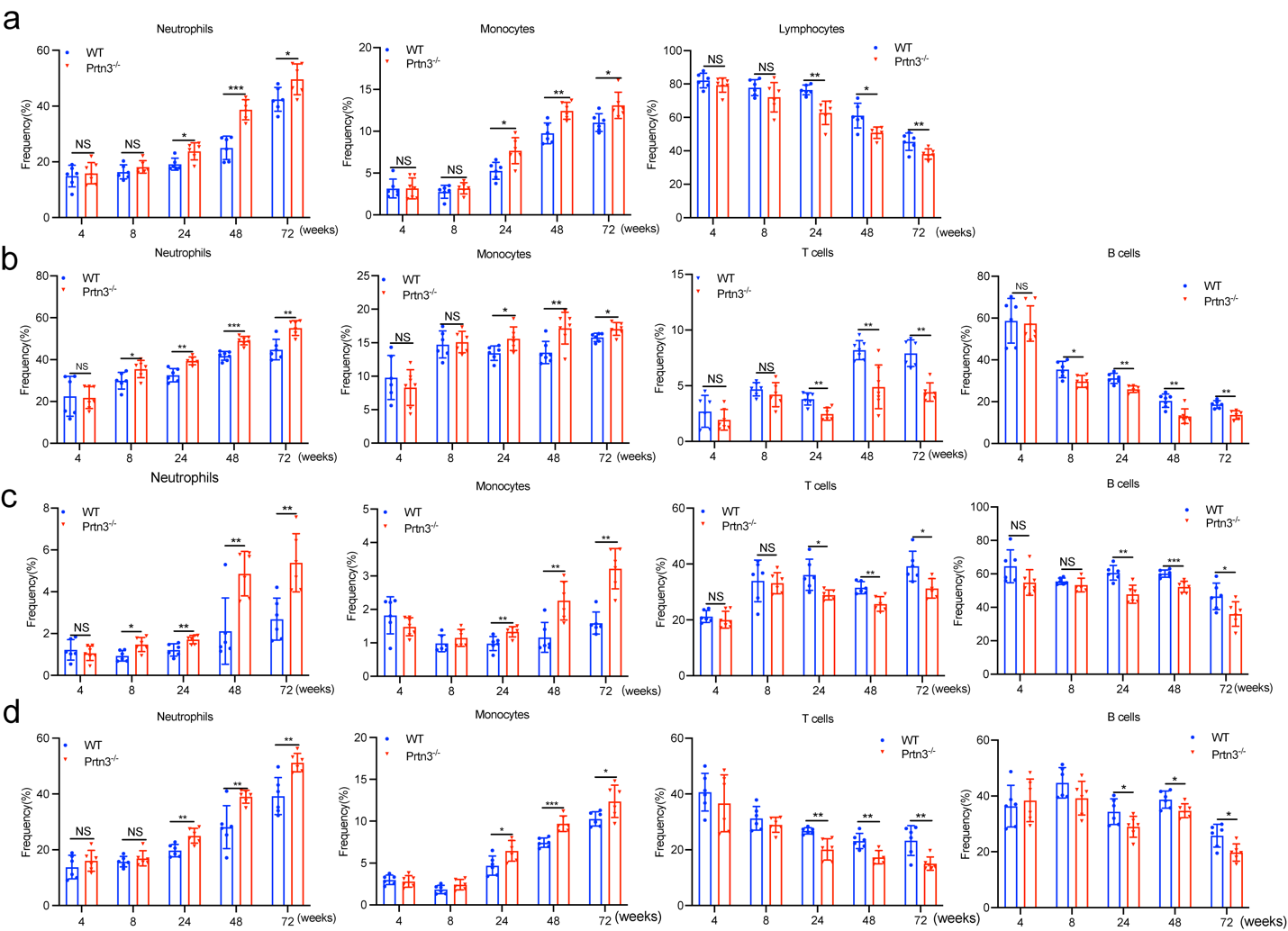


**Figure S2 Age-dependent aggravation of spontaneous increases myeloid cell populations.** Quantification of the frequency and number of the neutrophils and monocytes, T cells, and B cells populations in peripheral blood, revealed by routine analysis of blood (a), bone marrow (b), spleen (c), and peripheral blood (d), from 4 weeks to 72 weeks-old mice WT and *Prtn3^-/-^* mice (n=6). ^*^*p*<0.05, ^**^*p*<0.01, ^***^*p*<0.001. Data are the mean ± s.d.; n: biologically independent experiments. Statistical analysis was performed using an unpaired two-tailed Student‘s t-test.


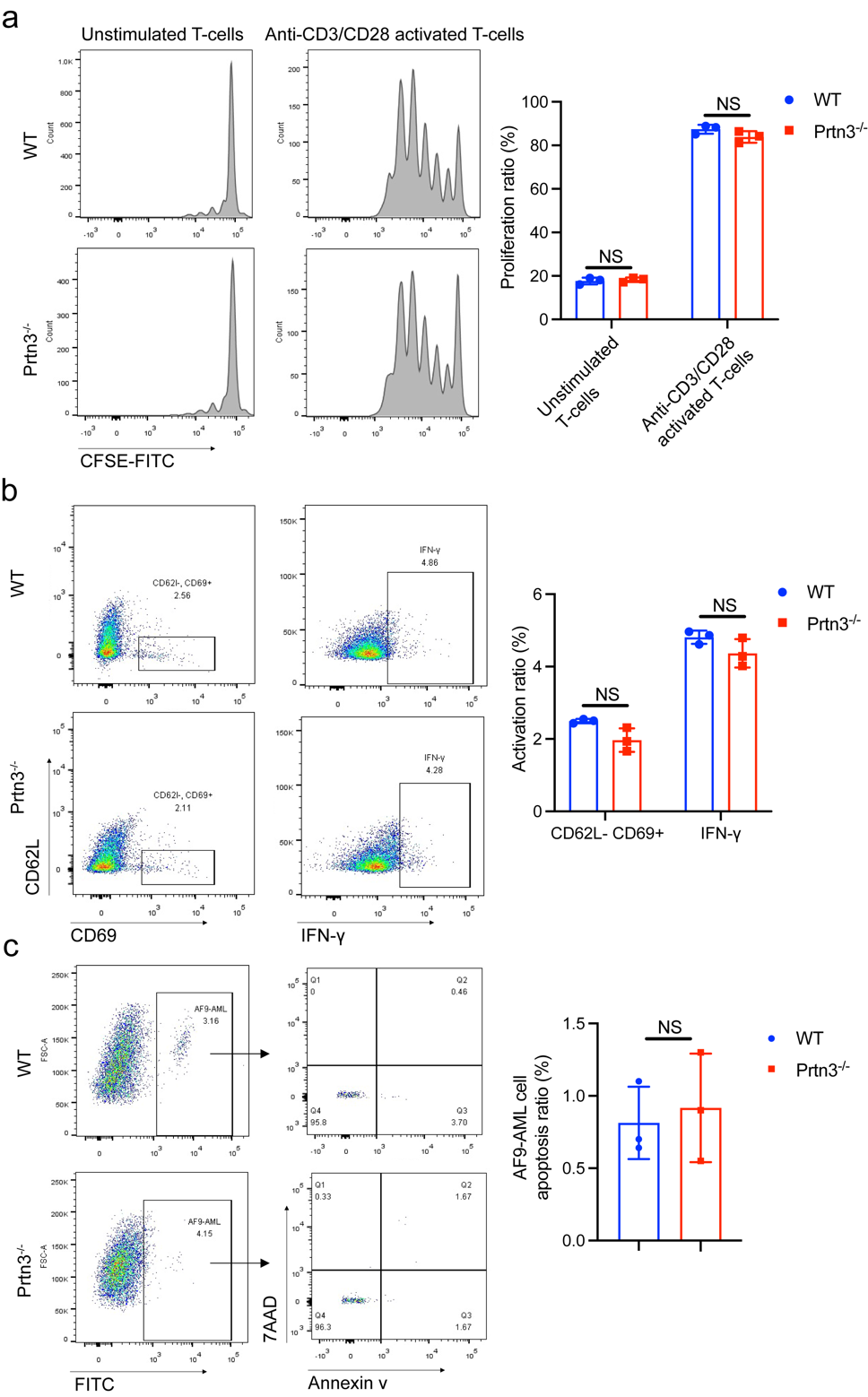


**Figure S3 Loss of PRTN3 does not affect the function of T cells.** (a) Representative flow cytometry plots and statistical quantitation for the proliferation T in WT and *Prtn3^-/-^* mice. Numbers denote the frequency of each population among live singlets (*n*=3). (b) Flow cytometry analysis and statistical quantitation of the percentage of activated T cells labeled with CD62L, CD69, IFN-γ, and TNF-α in the spleen of WT and *Prtn3*^-/-^ mice (n=3). (c) Representative images and statistical quantitation of FACS analysis of T cell-mediated elimination of AF9-AML cells, as determined by annexin V and 7AAD double labeling(*n*=3). NS: *p*>0.05, ^*^*p*<0.05, ^**^*p*<0.01, ^***^*p*<0.001. Data are the mean ± s.d.; n: biologically independent experiments. Statistical analysis was performed using an unpaired two-tailed Student‘s t-test.


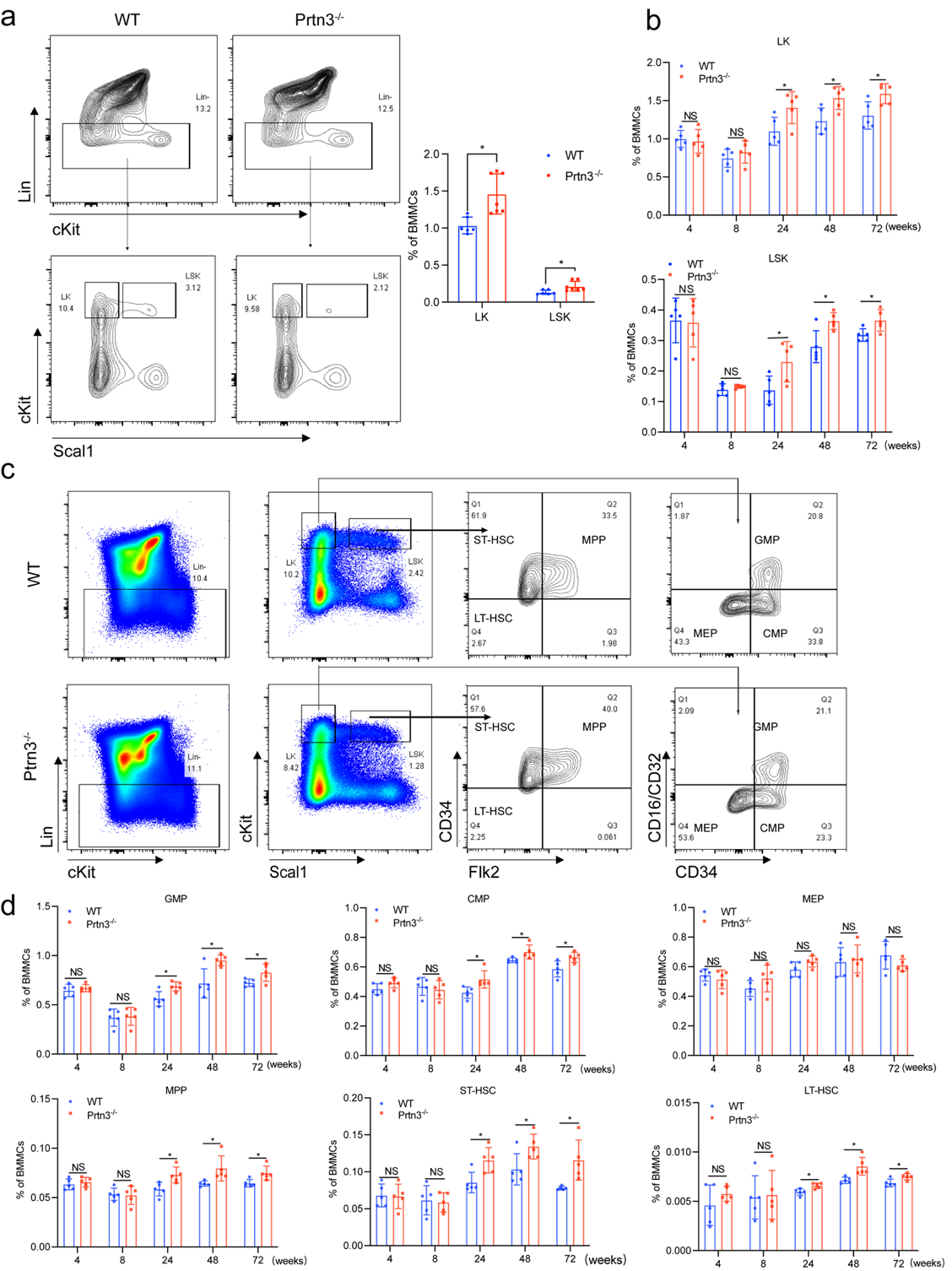


**Figure S4 Loss of PRTN3 affects hematopoietic stem/progenitor cell numbers.** (a) Representative flow cytometry plots for LK cells and LSK cells in WT and *Prtn3^-/-^* mice. Numbers denote the frequency of each population among live singlets (*n*=6). (b) quantification of the frequency and number (per one femur and one tibia) of LK cells and LSK cells in WT and *Prtn3^-/-^* mice at different ages (*n* = 6 per group). (c) Representative flow cytometry plots for GMP, CMP, MEP, MPP, ST-HSC, and LT-HSC cells in WT and *Prtn3^-/-^* mice. Numbers denote the frequency of each population among live singlets (*n*=6). (d) quantification of the frequency and number of GMP, CMP, MEP, MPP, ST-HSC, and LT-HSC in WT and *Prtn3^-/-^* mice at different ages (*n* = 6 per group), NS: *p*>0.05. Data are the mean ± s.d.; n: biologically independent experiments. Statistical analysis was performed using an unpaired two-tailed Student‘s t-test.


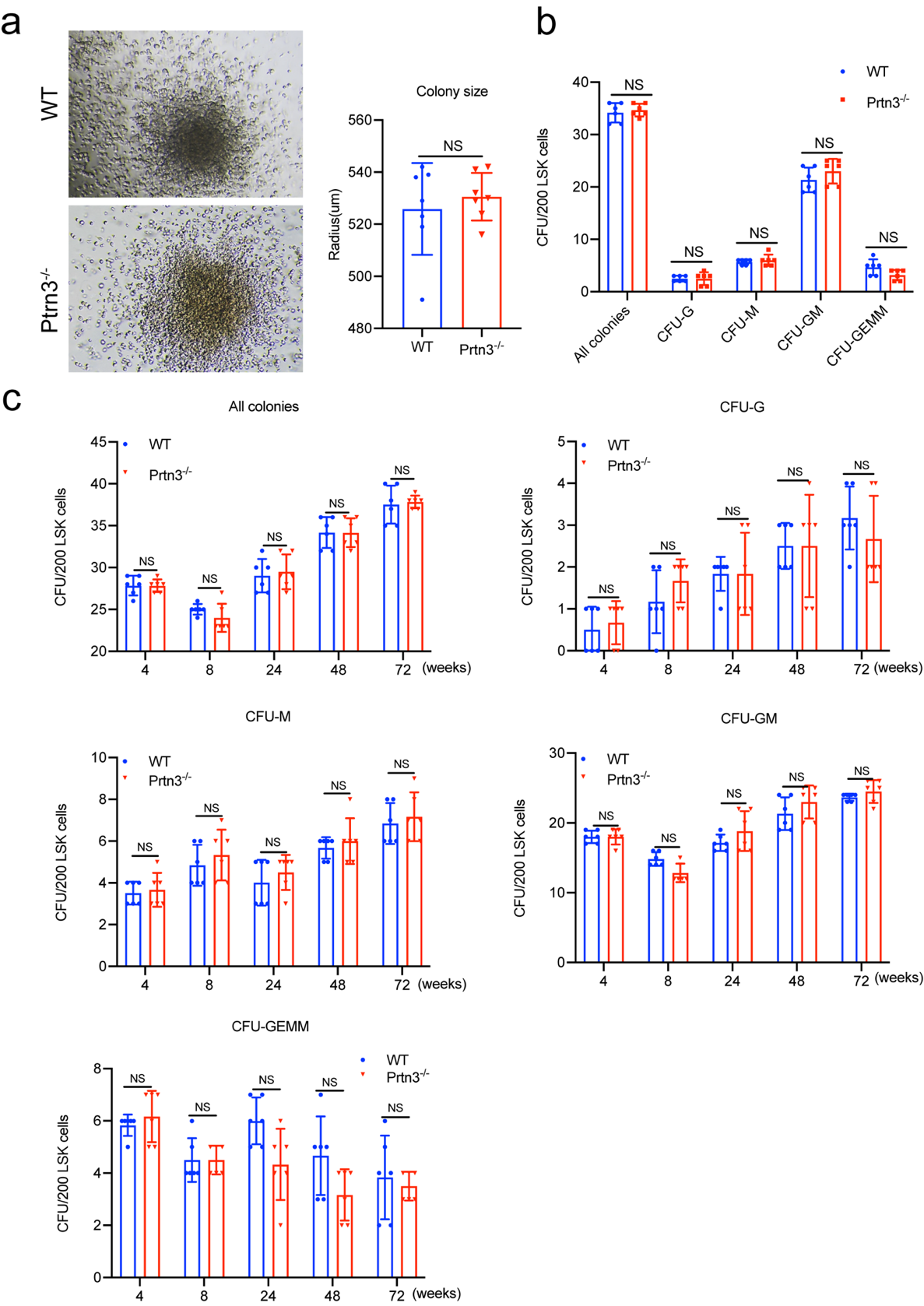


**Figure S5 Cultured the LSK cells in vitro from different ages.** (a) Representative images and Quantitative analysis of WT and *Prtn3^-/-^* colonies from colony-forming cell assays. Scale bar, 100 mm. (b) Quantification of in vitro progenitor cell activity as demonstrated by colony-forming cell assays using LSK cells (*n* = 6 per group). (c) Quantification of in vitro progenitor cell activity as demonstrated by colony-forming cell assays using LSK cells from different ages (*n* = 6 per group), NS: *p*>0.05. Data are the mean ± s.d.; n: biologically independent experiments. Statistical analysis was performed using an unpaired two-tailed Student‘s t-test.


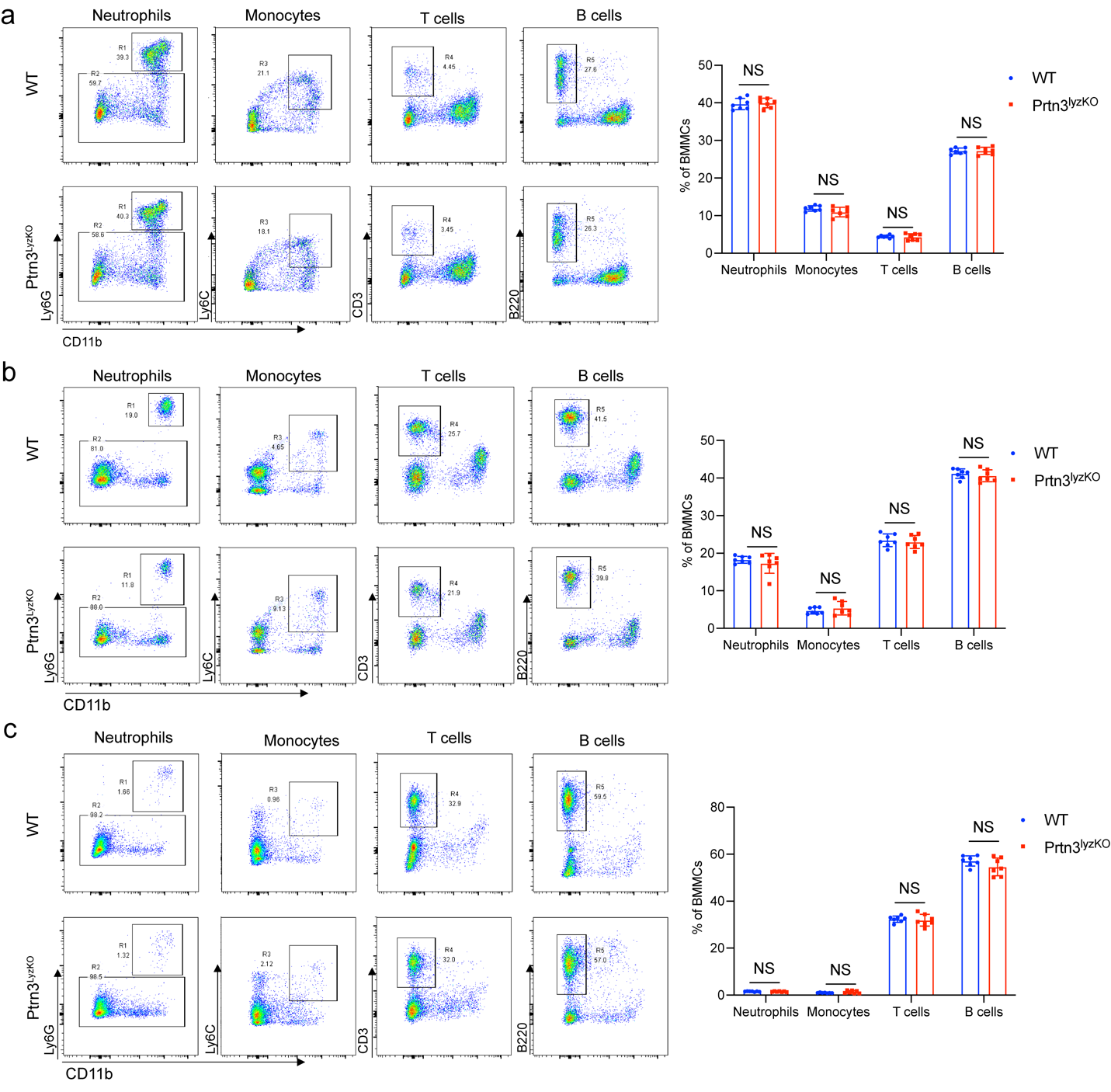


**Figure S6 Condition knockout PRTN3 in myeloid cells does not affect myeloid differentiation.** Flow cytometry analyzed the percentage of neutrophils, monocytes, T cells, and B cells in bone marrow (a), spleen (b), and peripheral blood (c) between WT and *Prtn3^LyzKO^* mice, NS: *p*>0.05. Data are the mean ± s.d.; n: biologically independent experiments. Statistical analysis was performed using an unpaired two-tailed Student‘s t-test.


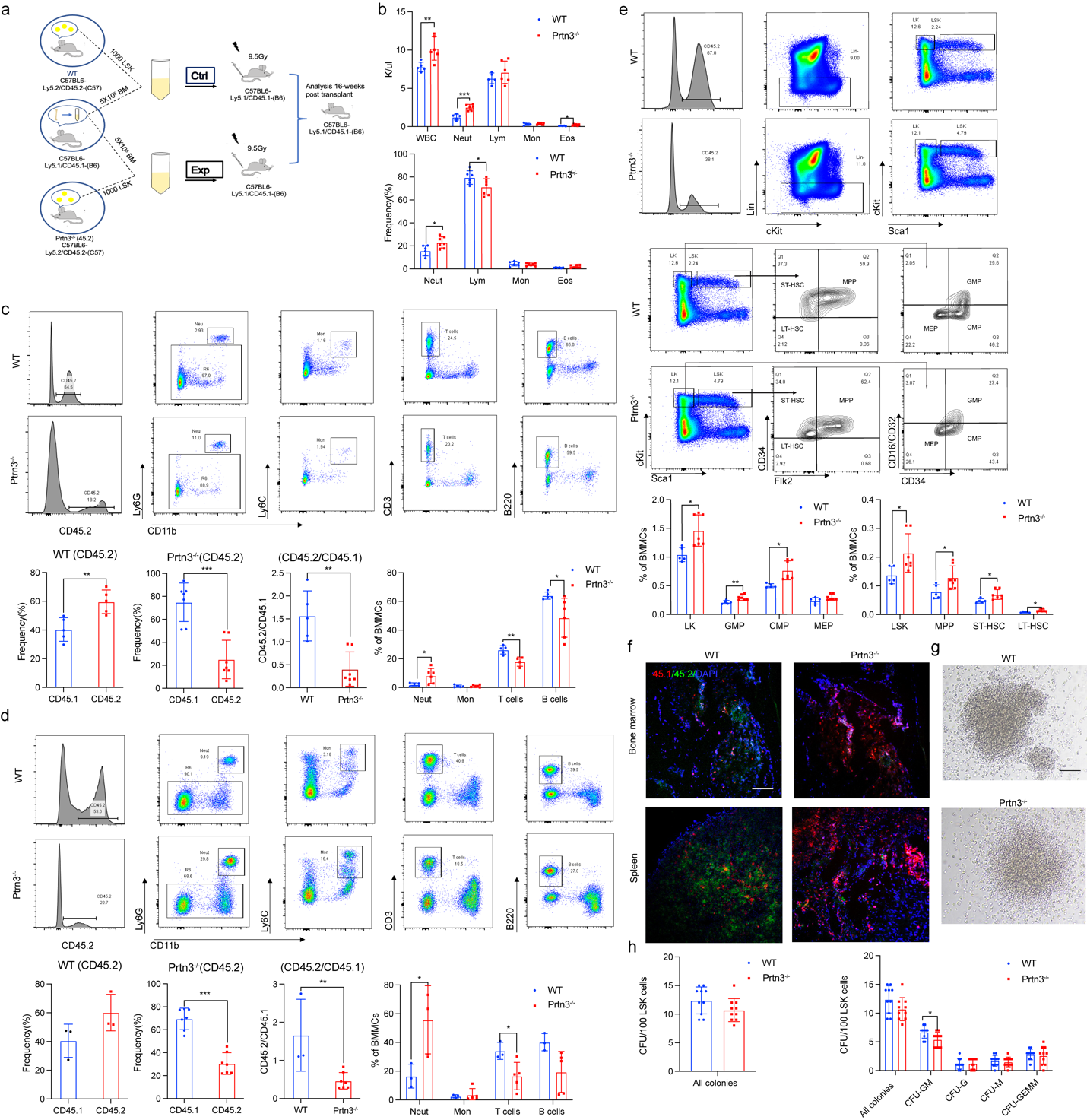


**Figure S7 Progenitor cells in 24-weeks-old *Prtn3^-/-^* mice outcompete WT cells in vivo.** (a) Scheme of the experimental setup for competitive bone marrow transplantation. (b) Quantification of the number and frequency of neutrophils, monocytes, T cells, and B cells in PB from the CD45.2 recipient mice. (c, d) Representative flow cytometry plots (up) for neutrophils, monocytes, T cells, and B cells in PB and spleen from the CD45.2 recipient mice (*n*=7). Numbers denote the frequency of each population among live singlets. Quantification of the frequency and number (down) of CD45.1, CD45.2, the ratio of CD45.2/CD45.1, neutrophils, monocytes, T cells, and B cells in PB and spleen from CD45.2 recipient mice (*n* = 7). (e) Representative flow cytometry plots and quantification of the frequency and number for LK cells, LSK cells, GMP, CMP, MEP, MPP, ST-HSC, and LT-HSC cells in WT and *Prtn3^-/-^* mice. Numbers denote the frequency of each population among live singlets (*n*=6). (f) immunofluorescence staining showed the CD45.1 and CD45.2 population in the bone marrow and spleen from CD45.2 recipient mice (*n* = 7). (G) Representative images and Quantitative analysis of WT and *Prtn3^-/-^* colonies from colony-forming cell assays. Scale bar, 100 mm. (B) Quantification of in vitro progenitor cell activity as demonstrated by colony-forming cell assays using LSK cells (*n* = 6 per group). scale bars, 50 μm; ^*^*p*<0.05, ^**^*p*<0.01, ^***^*p*<0.001. Data are the mean ± s.d.; n: biologically independent experiments. Statistical analysis was performed using an unpaired two-tailed Student‘s t-test.


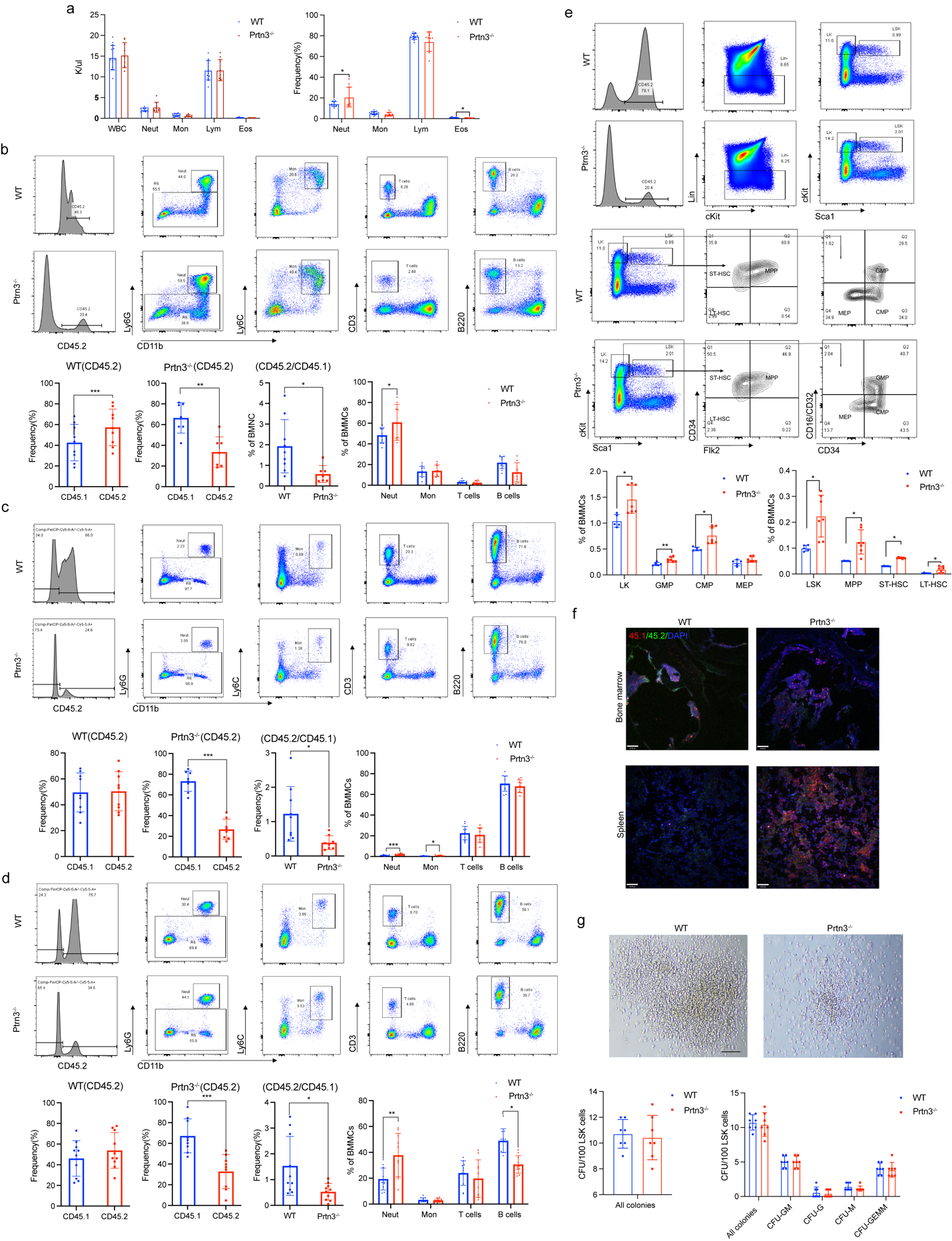


**Figure S8 Progenitor cells in 8-week-old Prtn3^-/-^ mice outcompete WT cells in vivo.** (a) Quantification of the number and frequency of neutrophils, monocytes, T cells, and B cells in PB from the CD45.2 recipient mice. (b, c, d) Representative flow cytometry plots (up) for neutrophils, monocytes, T cells, and B cells in bone marrow, PB, and spleen from the CD45.2 recipient mice (*n*=7). Numbers denote the frequency of each population among live singlets. Quantification of the frequency and number (down) of CD45.1, CD45.2, the ratio of CD45.2/CD45.1, neutrophils, monocytes, T cells, and B cells in PB and spleen from CD45.2 recipient mice (*n* = 7). (e) Representative flow cytometry plots and quantification of the frequency and number for LK cells, LSK cells, GMP, CMP, MEP, MPP, ST-HSC, and LT-HSC cells in WT and *Prtn3^-/-^* mice. Numbers denote the frequency of each population among live singlets (*n*=6). (f) immunofluorescence staining showed the CD45.1 and CD45.2 population in the bone marrow and spleen from CD45.2 recipient mice (*n* = 7). (g) (up) Representative images and Quantitative analysis of WT and *Prtn3^-/-^* colonies from colony-forming cell assays. Scale bar, 100 mm. (down) Quantification of in vitro progenitor cell activity as demonstrated by colony-forming cell assays using LSK cells (*n* = 6 per group). scale bars, 50 μm; ^*^*p*<0.05, ^**^*p*<0.01, ^***^*p*<0.001. Data are the mean ± s.d.; n: biologically independent experiments. Statistical analysis was performed using an unpaired two-tailed Student‘s t-test.


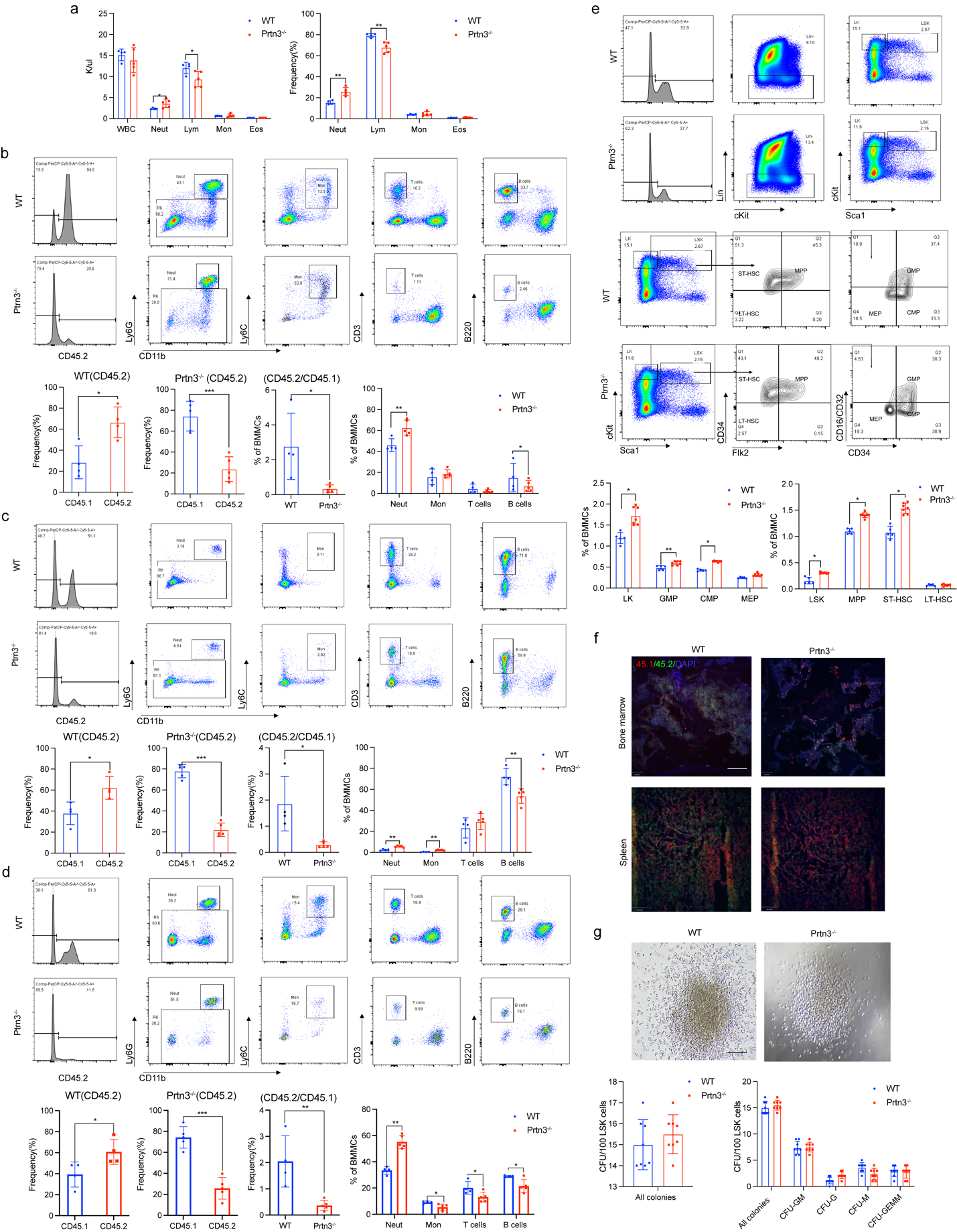


**Figure S9 Progenitor cells in 48-week-old *Prtn3^-/-^* mice outcompete WT cells in vivo.** (a) Quantification of the number and frequency of neutrophils, monocytes, T cells, and B cells in PB from the CD45.2 recipient mice. (b, c, d) Representative flow cytometry plots (up) for neutrophils, monocytes, T cells, and B cells in bone marrow, PB, and spleen from the CD45.2 recipient mice (*n*=7). Numbers denote the frequency of each population among live singlets. Quantification of the frequency and number (down) of CD45.1, CD45.2, the ratio of CD45.2/CD45.1, neutrophils, monocytes, T cells, and B cells in PB and spleen from CD45.2 recipient mice (*n* = 7). (e) Representative flow cytometry plots and quantification of the frequency and number for LK cells, LSK cells, GMP, CMP, MEP, MPP, ST-HSC, and LT-HSC cells in WT and *Prtn3^-/-^* mice. Numbers denote the frequency of each population among live singlets (*n*=6). (f) immunofluorescence staining showed the CD45.1 and CD45.2 population in the bone marrow and spleen from CD45.2 recipient mice (*n* = 7). (g) (up) Representative images and Quantitative analysis of WT and *Prtn3^-/-^* colonies from colony-forming cell assays. Scale bar, 100 mm. (down) Quantification of in vitro progenitor cell activity as demonstrated by colony-forming cell assays using LSK cells (*n* = 6 per group). scale bars, 50 μm; ^*^*p*<0.05, ^**^*p*<0.01, ^***^*p*<0.001. Data are the mean ± s.d.; n: biologically independent experiments. Statistical analysis was performed using an unpaired two-tailed Student‘s t-test.


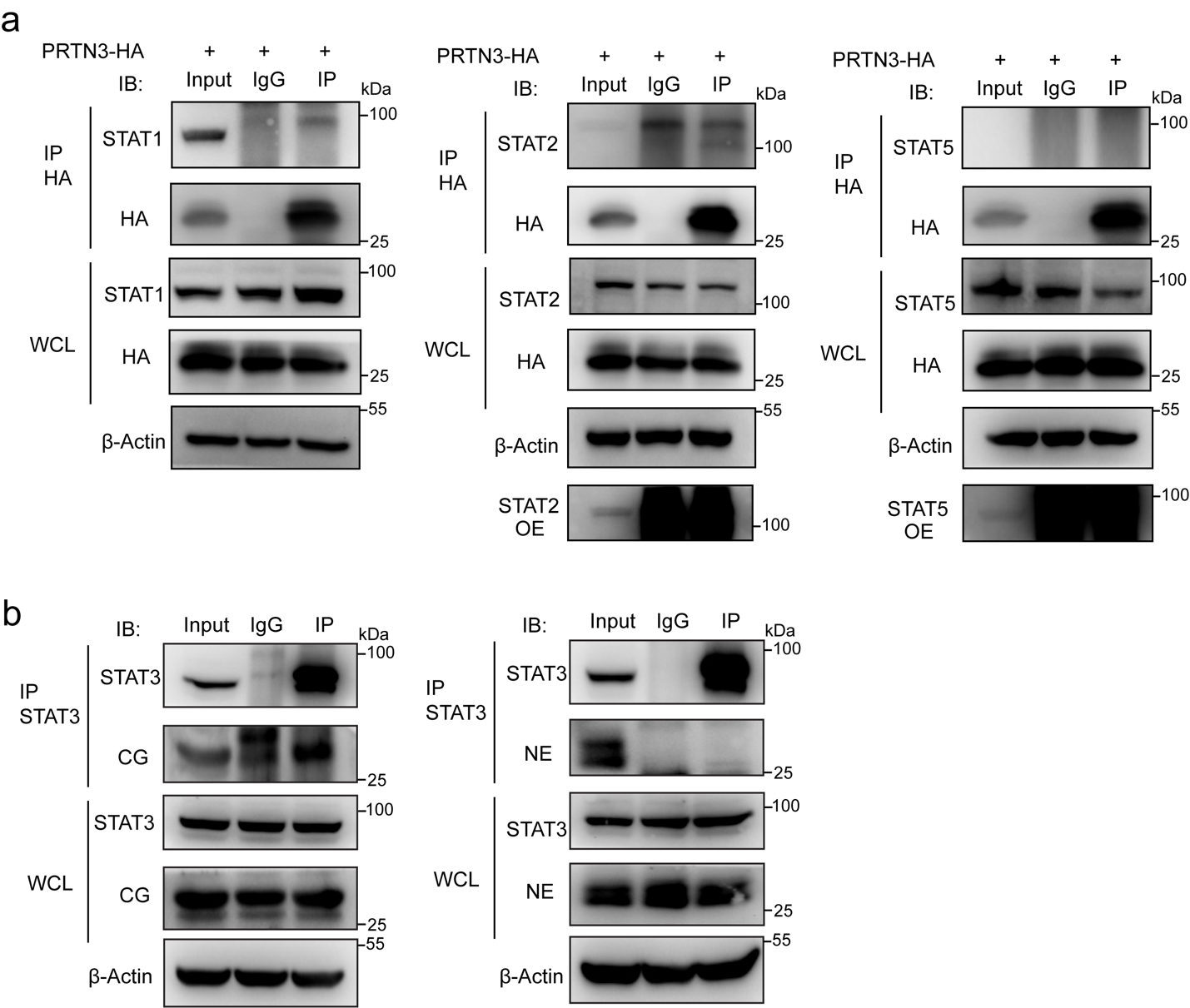


**Figure S10 PRTN3 does not interact with STAT1, STAT2, and STAT5; STAT3 did not interact with CG and NE.** (a) Western blot (WB) analysis of the Co-IP complex confirmed that PRTN3-HA did not bind to STAT1, STAT2, and STAT5 in HEK 293T cells (*n*=3); (b) western blot (WB) analysis of the Co-IP complex confirmed that STAT3 did not bind to NE and CG in HL-60 cells (*n*=3). Data are the mean ± s.d.; n: biologically independent experiments. Statistical analysis was performed using an unpaired two-tailed Student‘s t-test.


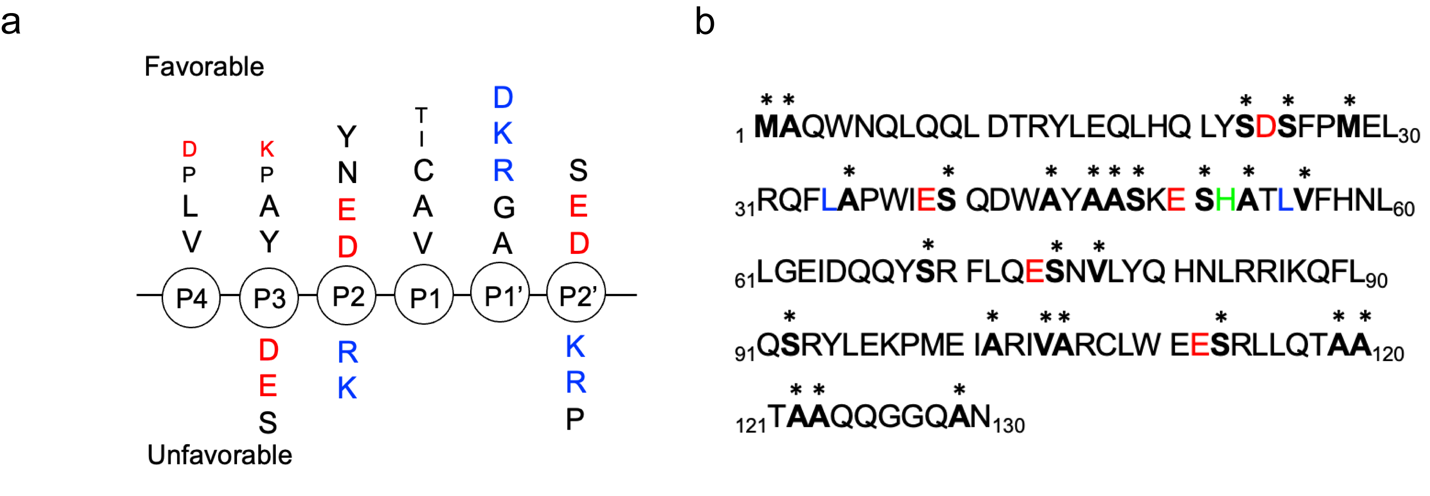


**Figure S11 Predicted cleavage site of PRTN3 in the N-terminal domain of STAT3 according to its proteolytic specificity.** (a) Subsite preferences of PRTN3 were obtained by kinetic analysis of the cleavage of synthetic substrates. (b) P1 residues of potential PRTN3 cleavage sites are shown by asterisk. P2 residues (arginine, aspartate, and histidine) that could alter PRTN3 processing are colored.


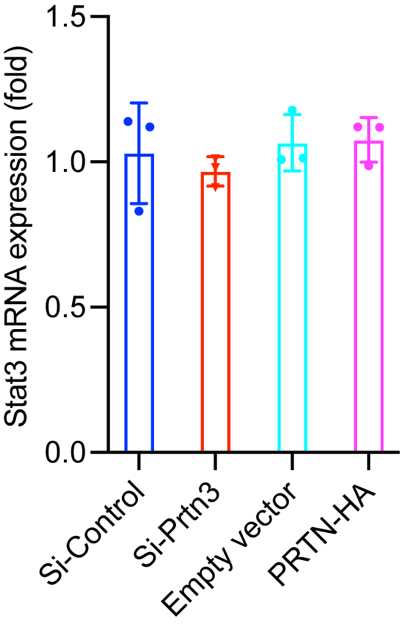


**Figure S12 mRNA expression of STAT3 in gain-, loss-PRTN3.** messenger RNA (mRNA) of Stat3 was determined in CD34+ cells with Si-Control or Si-Prtn3 treatment or HEK293T transfected with empty vector or PRTN3-HA (*n*=3). Data are the mean ± s.d.; n: biologically independent experiments. Statistical analysis was performed using an unpaired two-tailed Student‘s t-test.


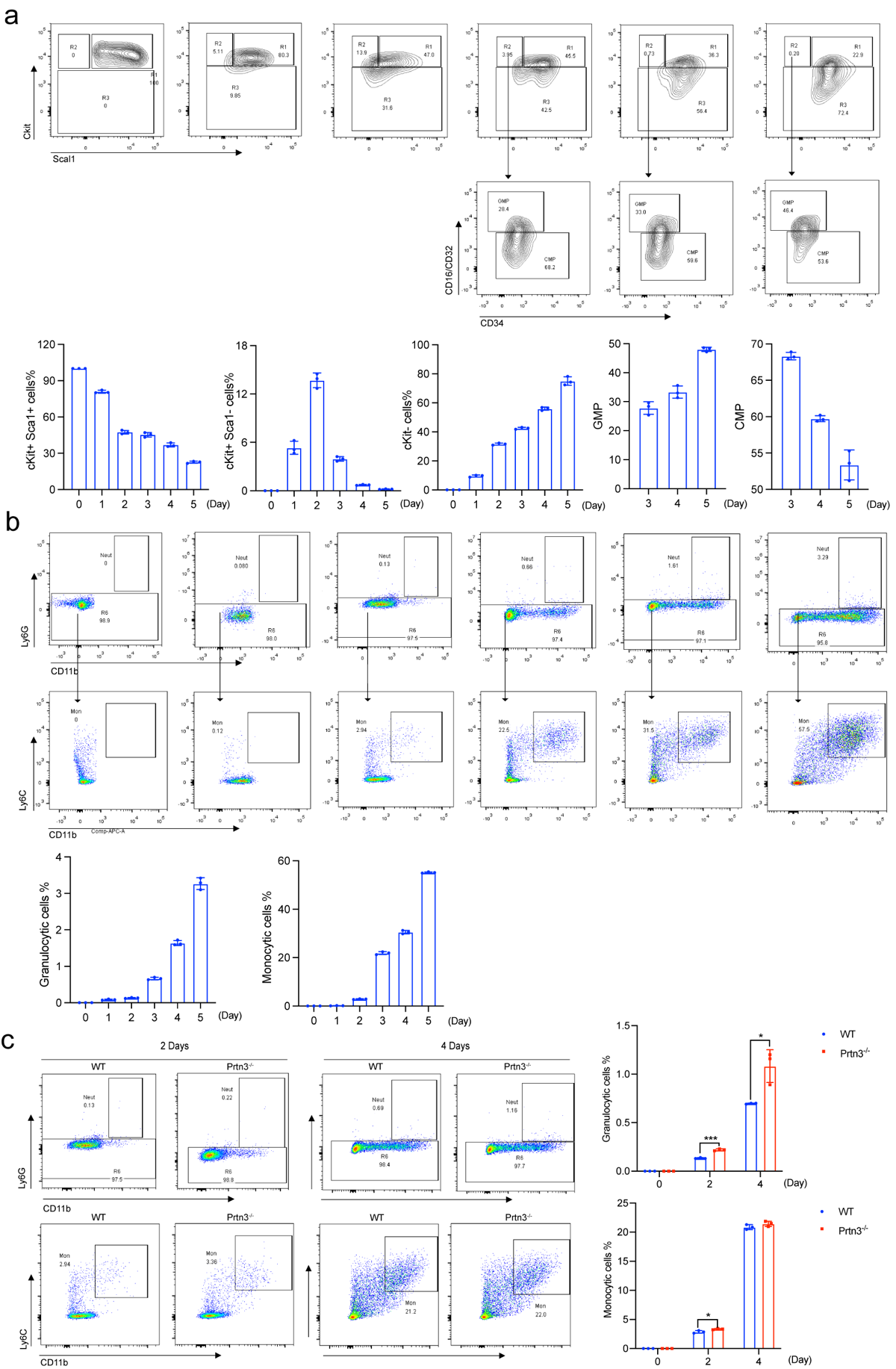


**Figure S13 Knockdown of PRTN3 promotes the percentage of neutrophils and monocytic cells in ex vivo myeloid cultures.** (a, b) Schematic of the ex vivo myeloid culture using LSK cells in myeloid differentiation media containing stem cell factor and G-CSF. Cells in the culture were analyzed each day starting from days 1 to 5 with multiple antibodies to delineate the appearance of common myeloid progenitor (CMP; Sca-1−cKit+CD34+ CD16/32−), granulocyte-monocyte progenitor (GMP; Sca-1−cKit+CD34+ CD16/32+), monocytic (CD11b+MCSFR1+Gr1+), and granulocytic (CD11b+MCSFR1−Gr1+) cells in the culture. (c) The percentage of neutrophils and monocytes original from Lineage− Sca-1+c-Kit+ (LSK) cells from WT and *Prtn3^-/-^* mice were used for ex vivo myeloid culture (*n*=3). ^*^*p*<0.05, ^**^*p*<0.01, ^***^*p*<0.001. Data are the mean ± s.d.; n: biologically independent experiments. Statistical analysis was performed using an unpaired two-tailed Student‘s t-test.


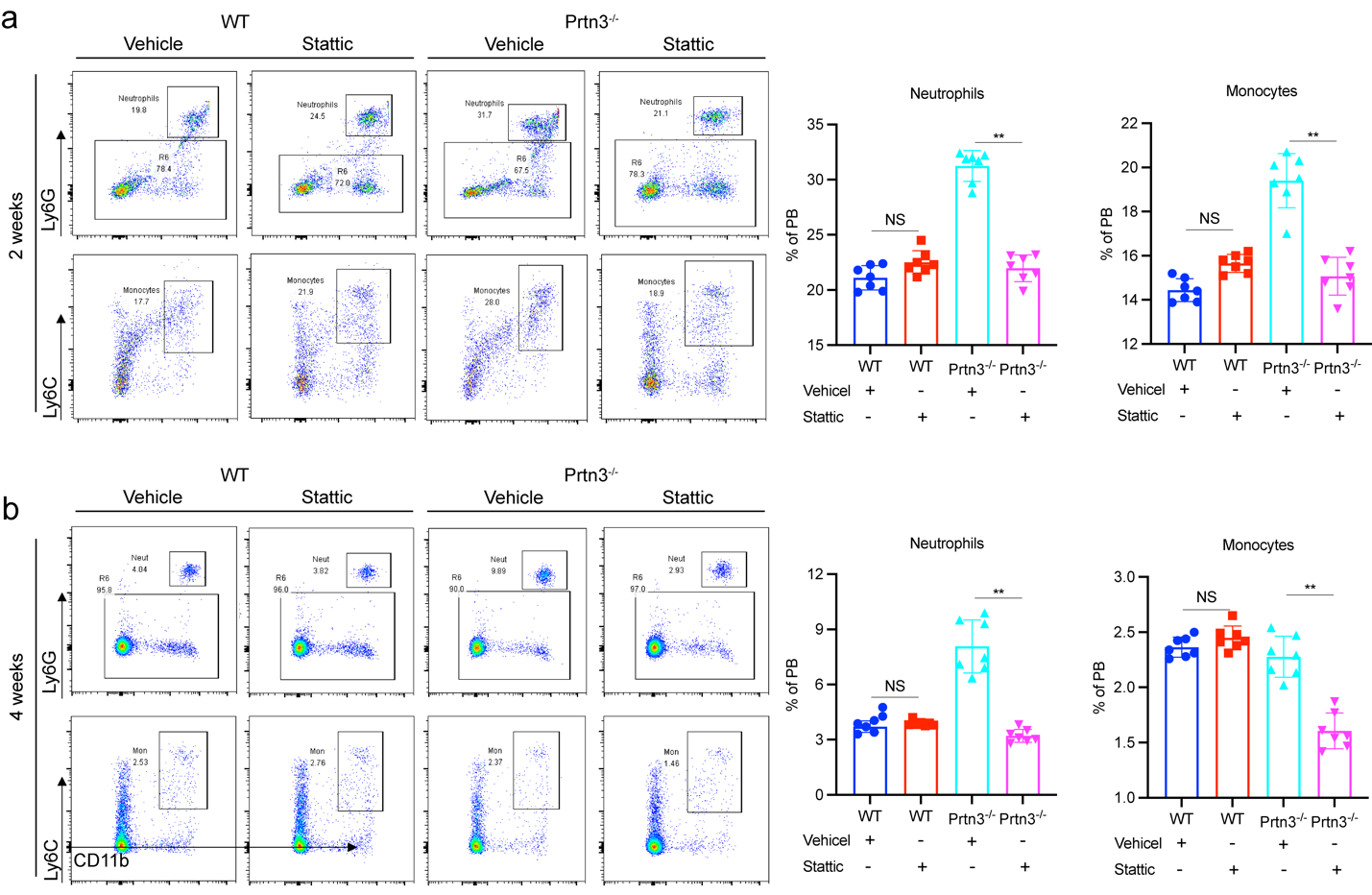


**Figure S14 Flow cytometry analysis of the percentage of neutrophils and monocytes in PB from mice with STAT3 inhibitor treatment.** Myeloid cells in the peripheral blood of *Prtn3^-/-^* and WT mice every 2 weeks (a) and 4 weeks (b). ^*^*p*<0.05, ^**^*p*<0.01, ^***^*p*<0.001. Data are the mean ± s.d.; n: biologically independent experiments. Statistical analysis was performed using an unpaired two-tailed Student‘s t-test.


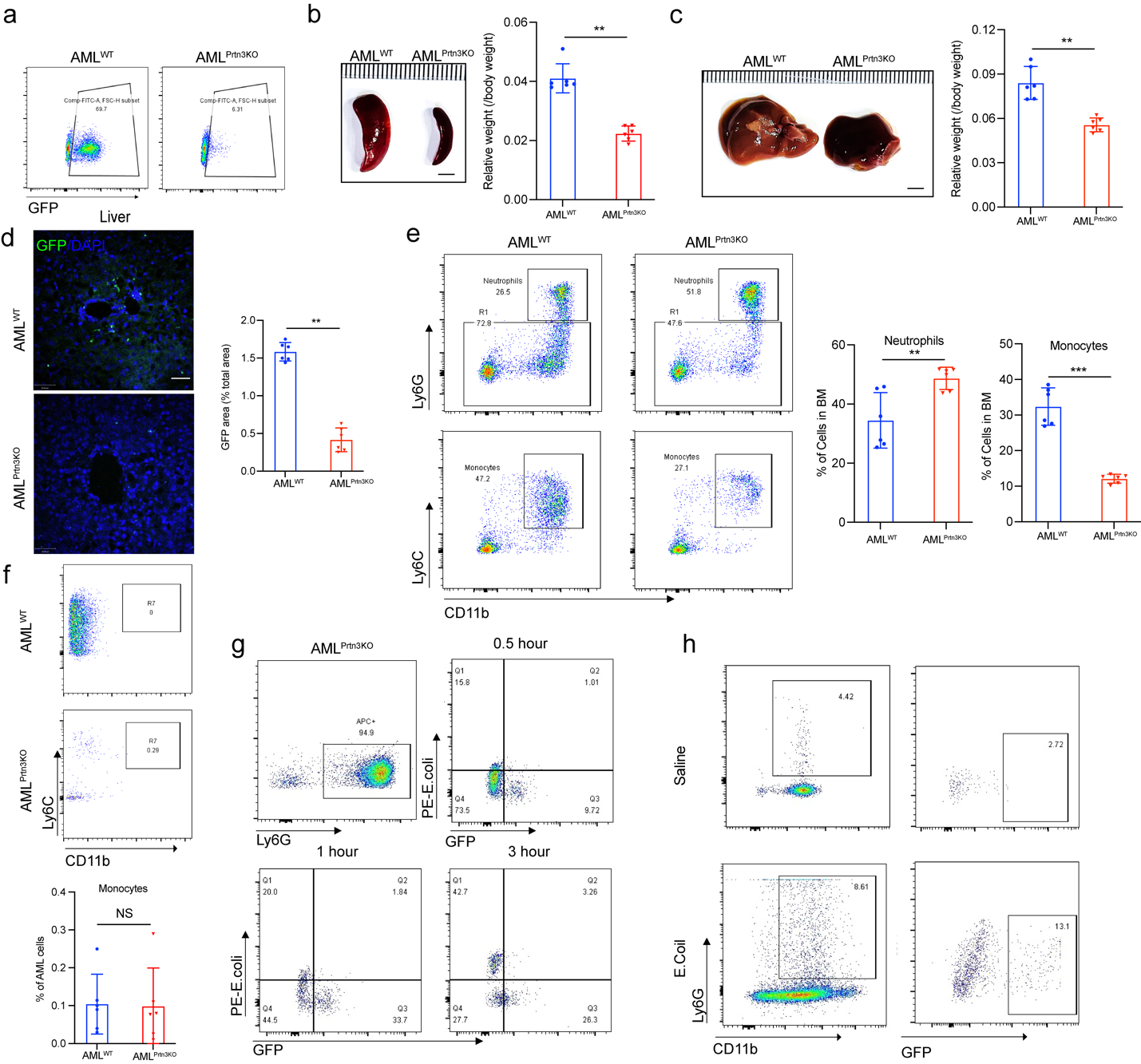


**Figure S15 The phenotype of mice with WT and *Prtn3^-/-^* AML treatment.** (a) Flow cytometry analysis of the percentage of GFP+ leukemia cells in the peripheral blood (PB) of recipients 30 days after transplantation (n = 6). Recipient spleen(b), liver size(c), immunofluorescence staining of GFP+ leukemia cells in the liver(d) 30 days after transplantation (*n* = 6). (e) Flow cytometry (left) and histogram (right) analysis shows the percentages of total neutrophils and monocytes from bone marrow in the BM of recipients (*n* =6). (f) Flow cytometry (up) and histogram (down) analysis shows the percentages of monocytes from AML cells (GFP+) in the BM of recipients (n =6). (g) Flow cytometry analysis shows the percentages of GFP+ neutrophils with PE-E.coli uptake from BM of AML^Prtn3KO^ recipients at different time points (*n* =3). (h) Flow cytometry analysis shows the percentages of GFP+ neutrophils capable of chemotaxis migration in the abdominal cavity of AML^Prtn3KO^ recipients with E. coli injection for 6 hours (*n* =3). ^*^*p*<0.05, ^**^*p*<0.01, ^***^*p*<0.001. Data are the mean ± s.d.; n: biologically independent experiments. Statistical analysis was performed using an unpaired two-tailed Student‘s t-test.


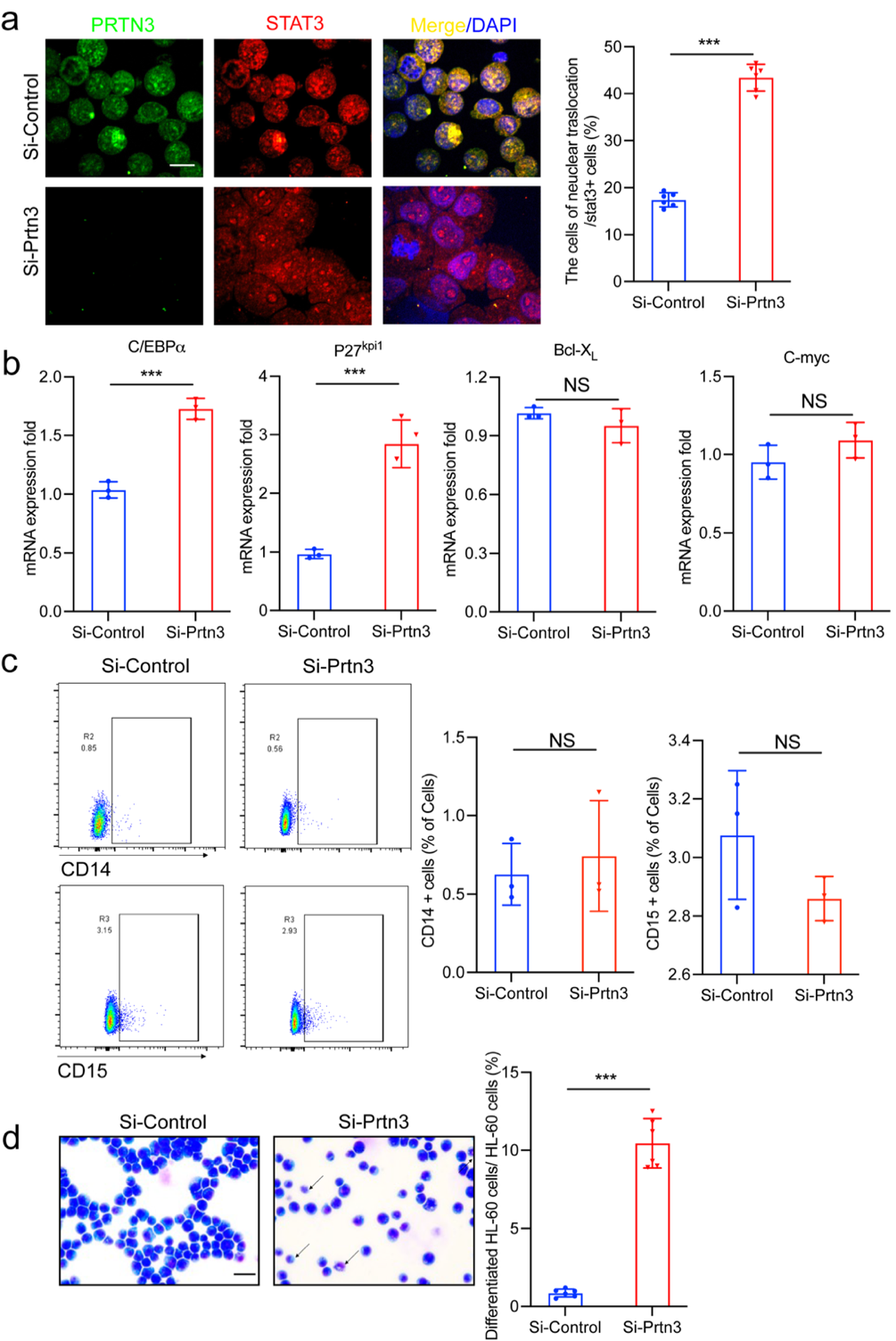


**Figure S16 The phenotype of HL60 cells with Prtn3-deficiency.** (a) immunofluorescence staining and quantification of the number of cells with nuclear translocation of STAT3 were analyzed in HL60 cells with Si-control or Si-*Prtn3* treatment for 24 hours (*n*=3). (b) messenger RNA (mRNA) of *C/EBPɑ*, *p27^kip1^*, *Bcl-X_L_*, and *C-myc* were determined in HL60 cells with Si-control or Si-*Prtn3* treatment for 24 hours (*n*=3); (c)Flow cytometry (left) and histogram (right) analysis of the percentage of CD14 and CD15 leukemia cells in the HL60 cells with Si-control or Si-*Prtn3* treatment for 7 days (*n* = 3). (d) The neutrophil-like cells in the HL60 cells with Si-control or Si-*Prtn3* treatment for 7 days (*n* = 3). ^*^*p*<0.05, ^**^*p*<0.01, ^***^*p*<0.001. Data are the mean ± s.d.; n: biologically independent experiments. Statistical analysis was performed using an unpaired two-tailed Student‘s t-test.


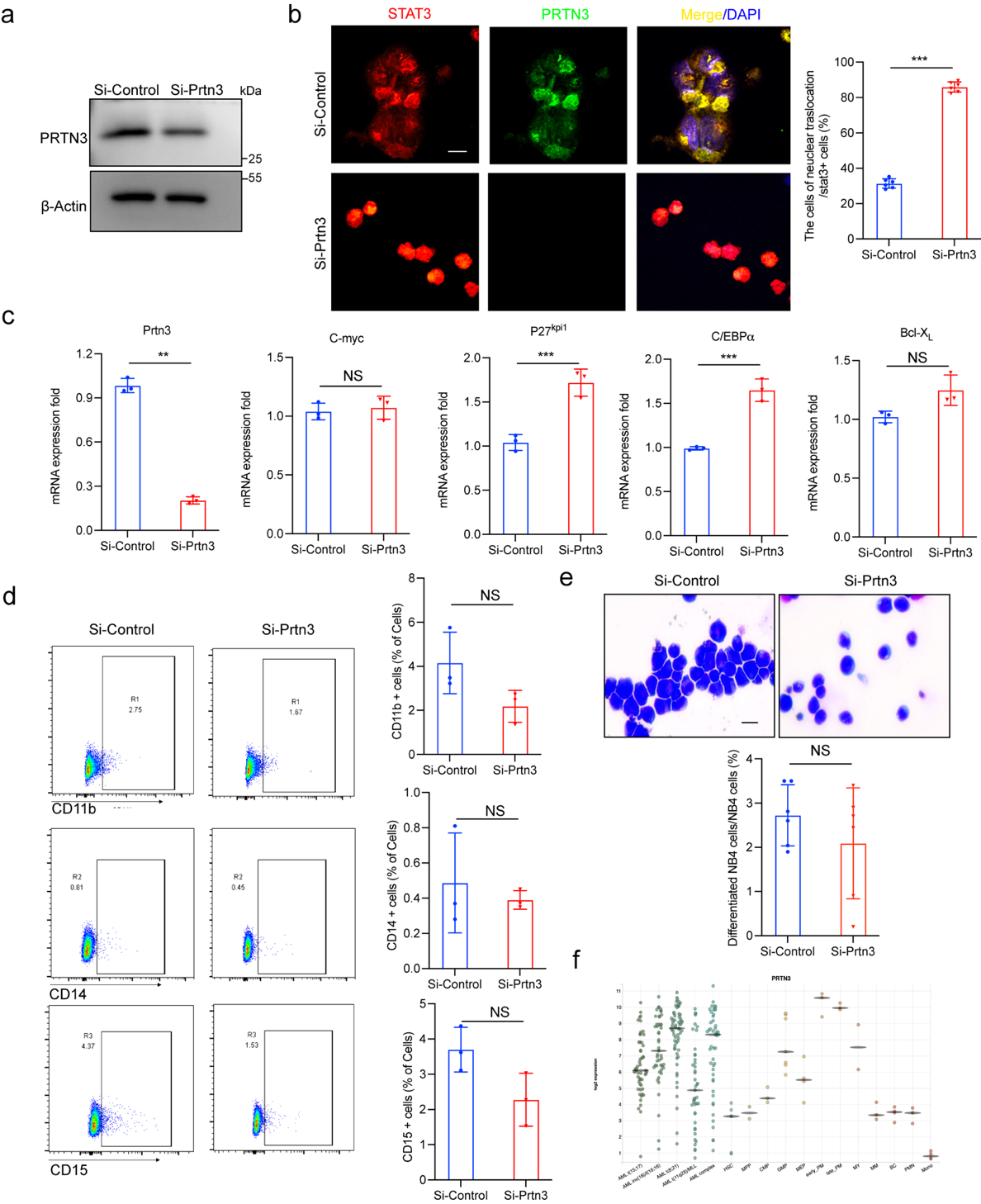


**Figure S17 The phenotype of NB4 cells with Prtn3-deficiency.** (a, b) protein expression, immunofluorescence staining, and quantification of the number of cells with nuclear translocation of STAT3 were analyzed in NB4 cells with Si-control or Si-*Prtn3* treatment for 24 hours (*n*=3). (c) messenger RNA of PRTN3, *C/EBPɑ*, *p27^kip1^*, *Bcl-X_L_*, and *C-myc* were determined in HL60 cells with Si-control or Si-*Prtn3* treatment for 24 hours (*n*=3); (D)Flow cytometry (left) and histogram (right) analysis of the percentage of CD11b, CD14 and CD15 leukemia cells in the NB4 cells with Si-control or Si-*Prtn3* treatment for 7 days (*n* = 3). (e) The neutrophil-like cells in the NB4 cells with Si-control or Si-*Prtn3* treatment for 7 days (*n* = 3). (f) Expression of PRTN3 in AML cells and normal hematopoietic 421 cells. Data is obtained from Bloodspot. ^*^*p*<0.05, ^**^*p*<0.01, ^***^*p*<0.001. Data are the mean ± s.d.; n: biologically independent experiments. Statistical analysis was performed using an unpaired two-tailed Student‘s t-test.


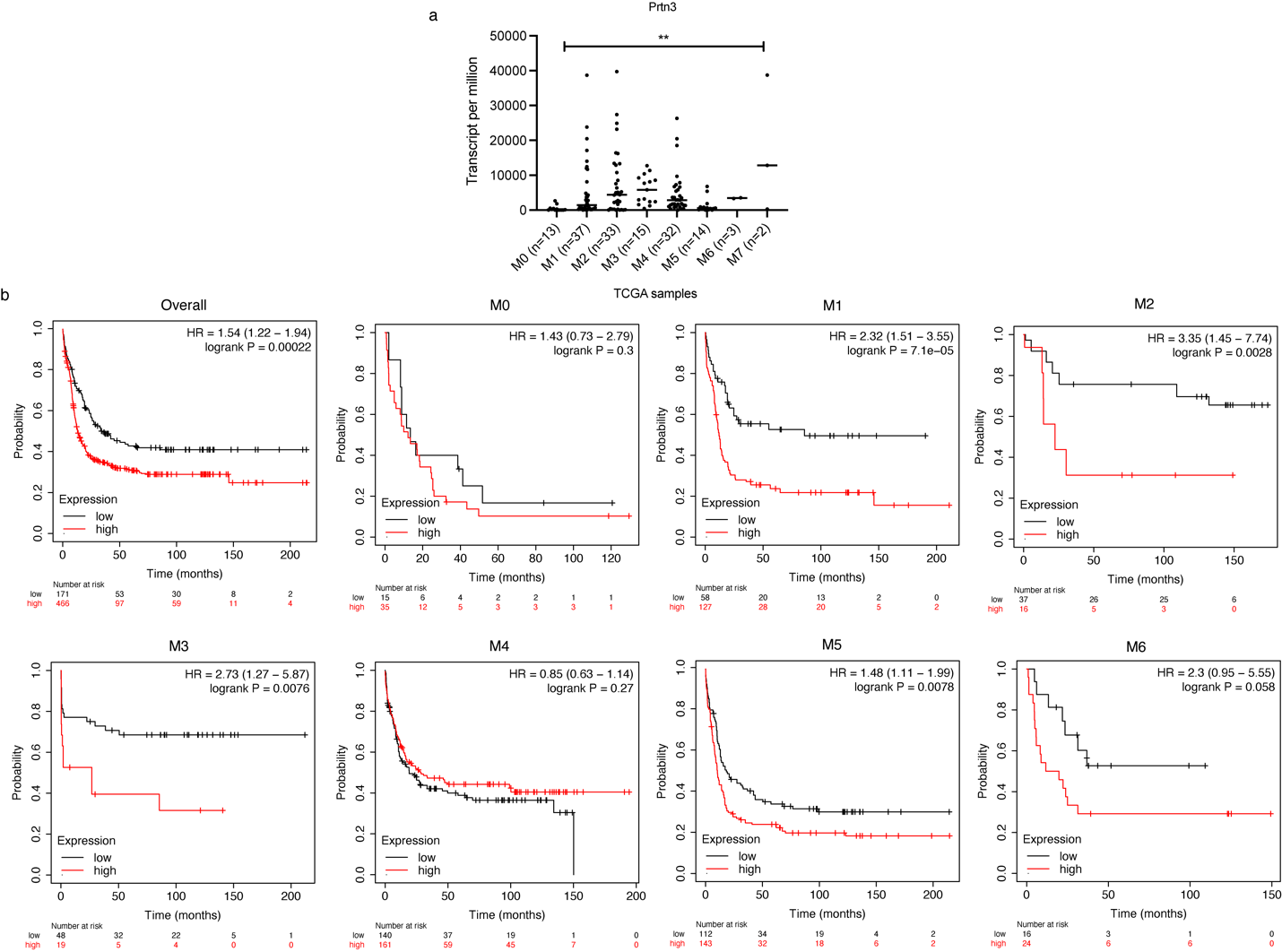


**Figure S18 The AML patient with PRTN3 expression survival probability on intermediate karyotype.**

(a) Expression of *Prtn3* in AML cells from M0 to M7 on FAB classification. (b) The *Prtn3* expression AML patient survival probability in overall and different AML subtypes on intermediate karyotype (https://kmplot.com/analysis/index.php?p=service). Data is obtained from the TCGA dataset and Kaplan-Meier Plotter. ^**^*p*<0.01.


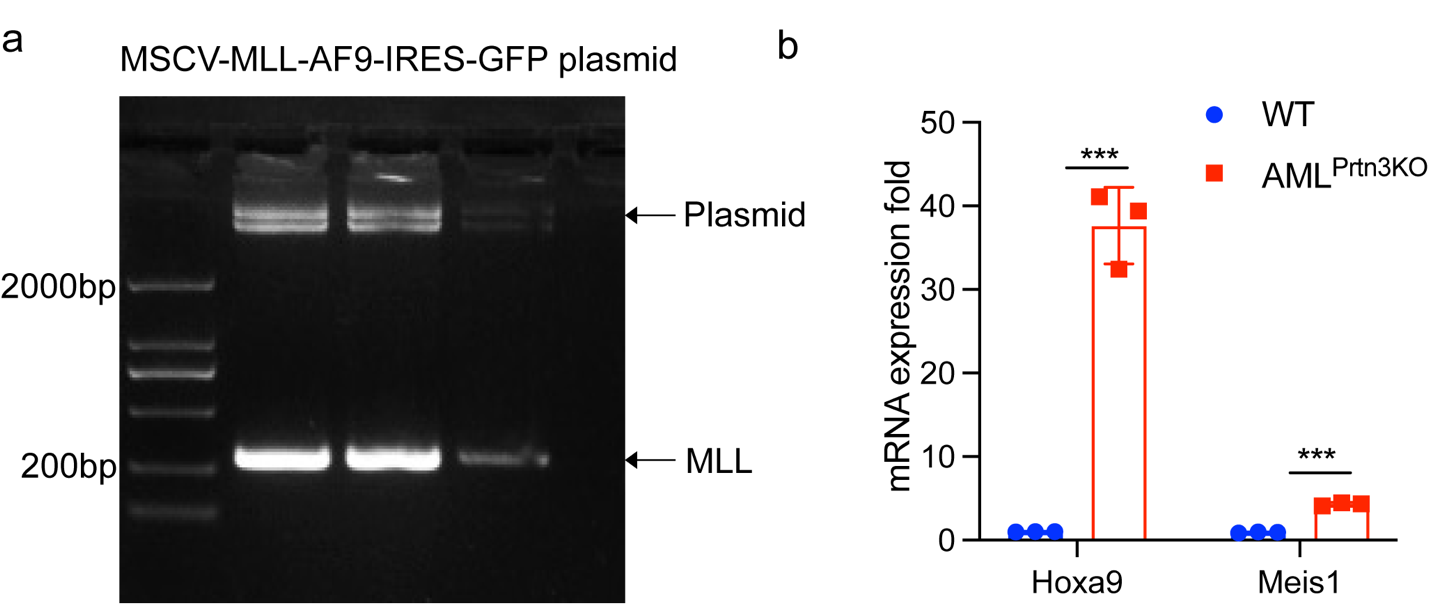


**Figure S19 MSCV-MLL-AF9-IRES-GFP plasmid examination by PCR and qPCR.** (a) the MLL-AF9 fragment was re-cloned from plasmid by MLL-AF9 primer and detect by PCR (n=3). (a) mRNA extracted from bone marrow of AML-Prtn3KO mice for confirming the expression of two key genes, Hoxa9 and Meis1, revealing by qPCR (n=3). ^***^*p*<0.001. Data are the mean ± s.d.; n: biologically independent experiments. Statistical analysis was performed using an unpaired two-tailed Student‘s t-test.
