# Supplementary material for: Proteinase 3 depletion attenuates leukemia by promoting myeloid differentiation
Source: Cell Death Differ. 2024 Apr 8;31(6):697–710. doi: 10.1038/s41418-024-01288-4 (PMC11165011; doi:10.1038/s41418-024-01288-4)

Figure 1

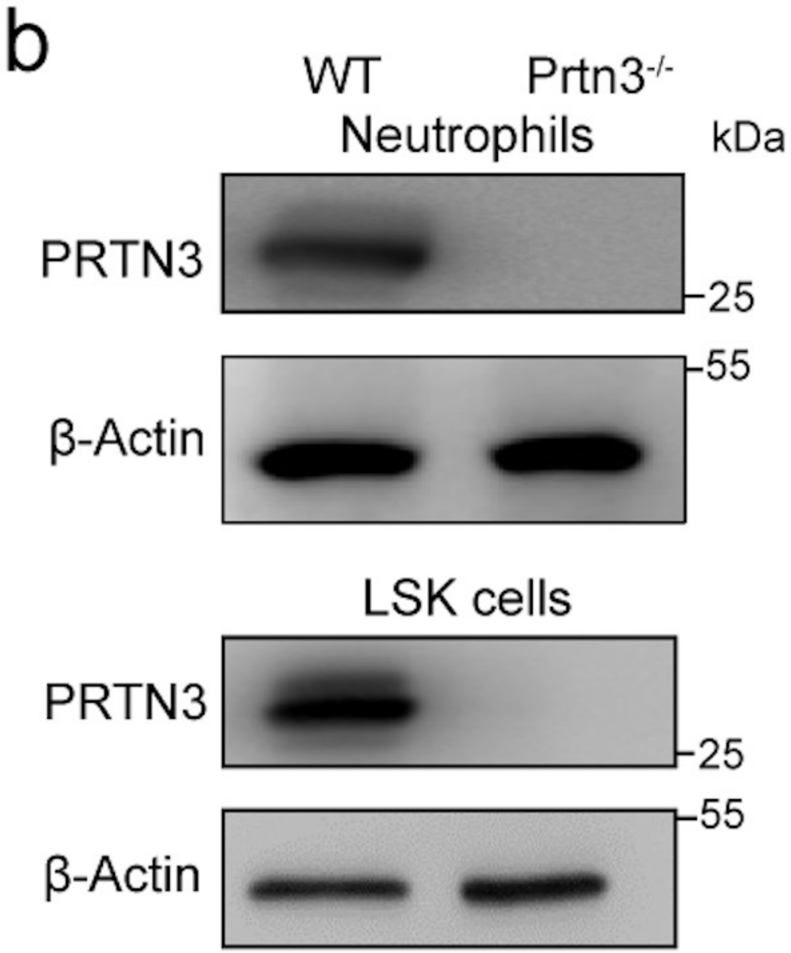
b
WT
Prtn3-/-
Neutrophils
kDa
PRTN3
25
β-Actin
55
LSK cells
PRTN3
25
β-Actin
55

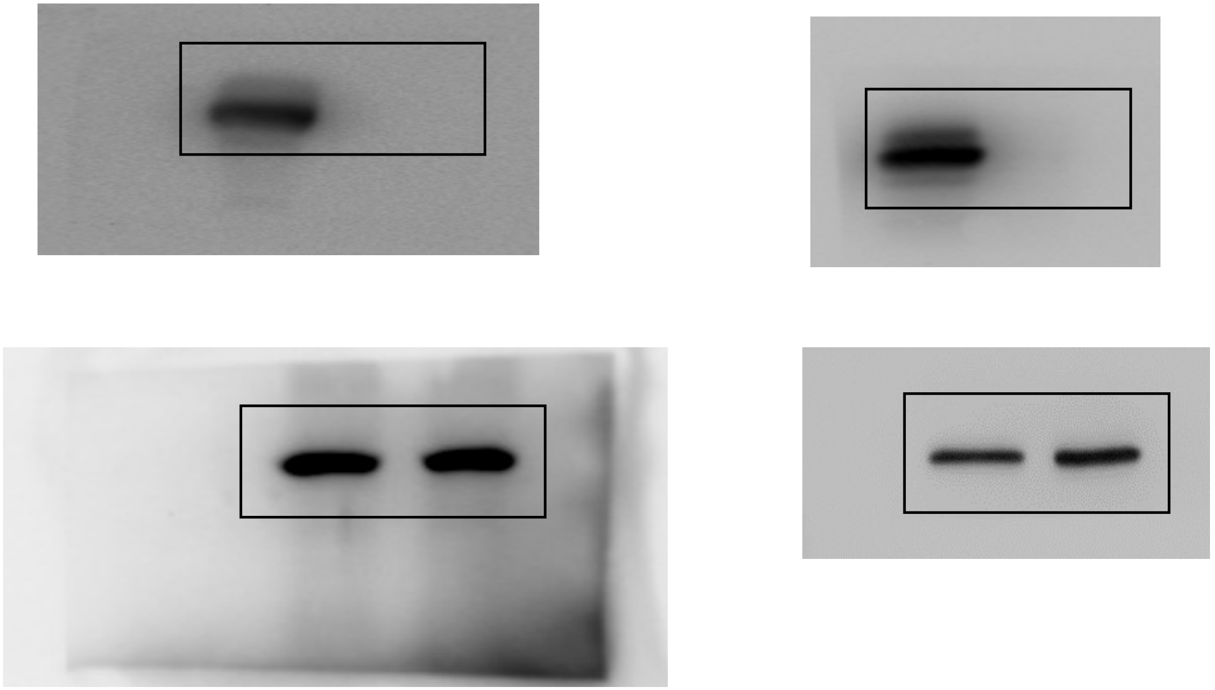

Figure 2

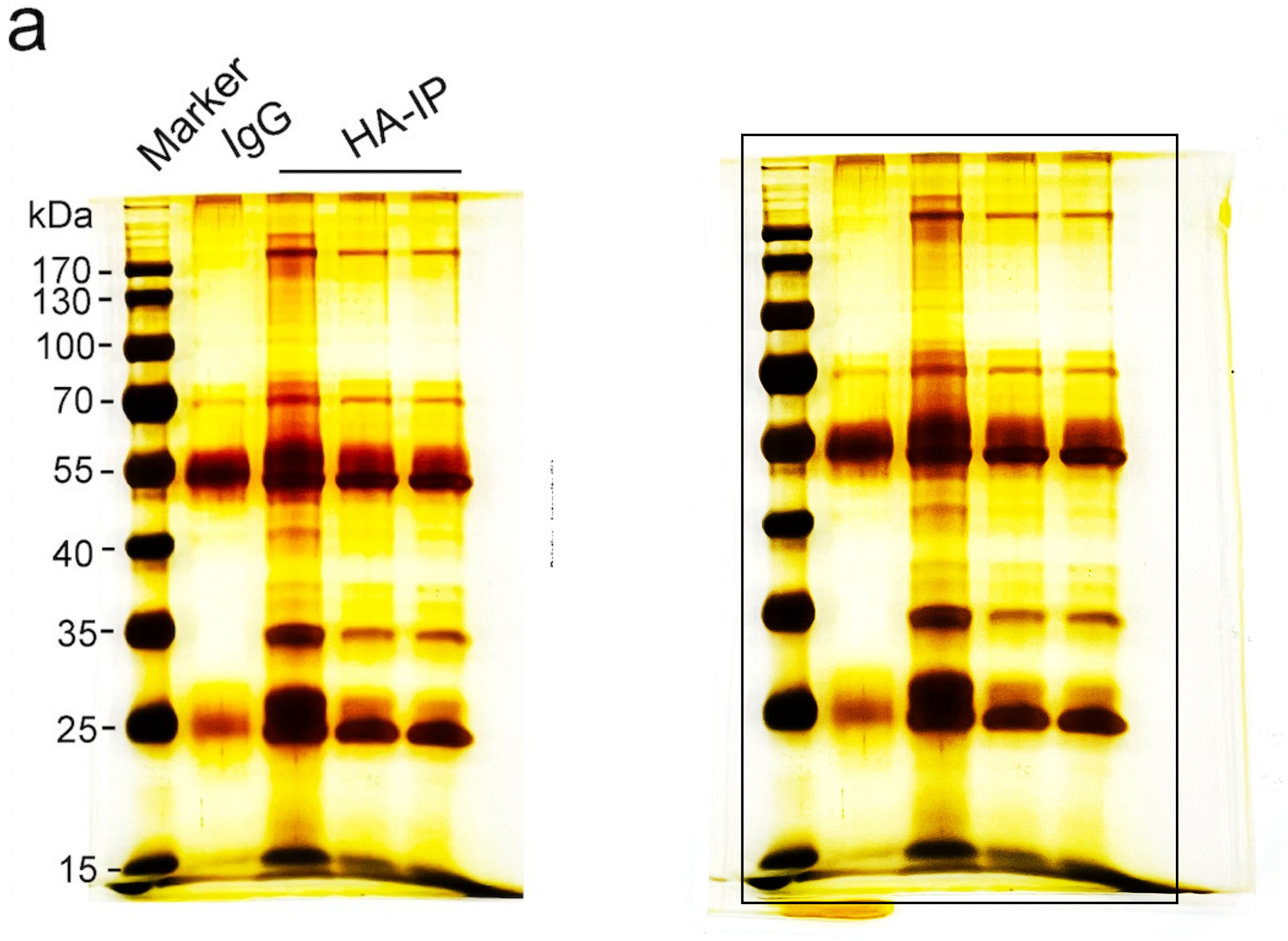

Figure 2

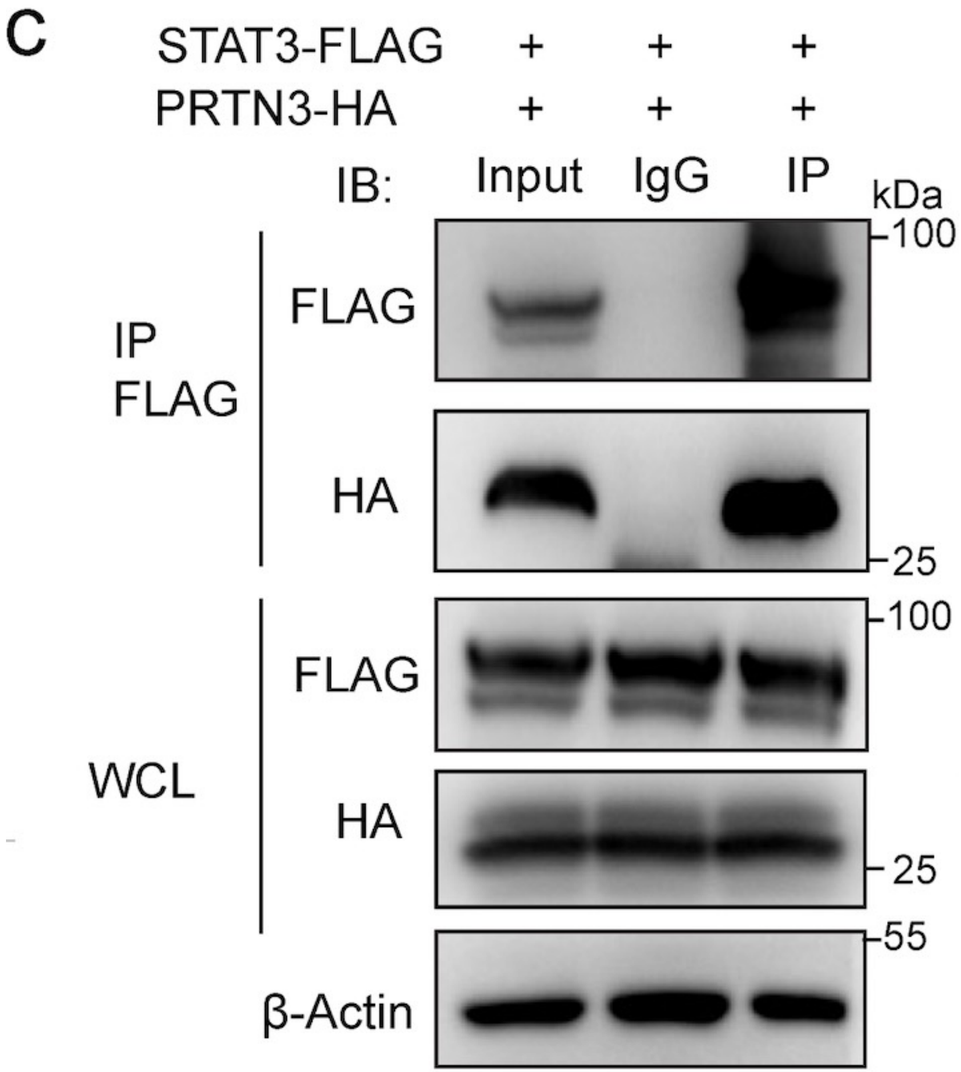
C
STAT3-FLAG + + +
PRTN3-HA + + +
IB: Input IgG IP
kDa
100
IP FLAG
FLAG
HA
25
100
WCL
FLAG
HA
25
55
β-Actin

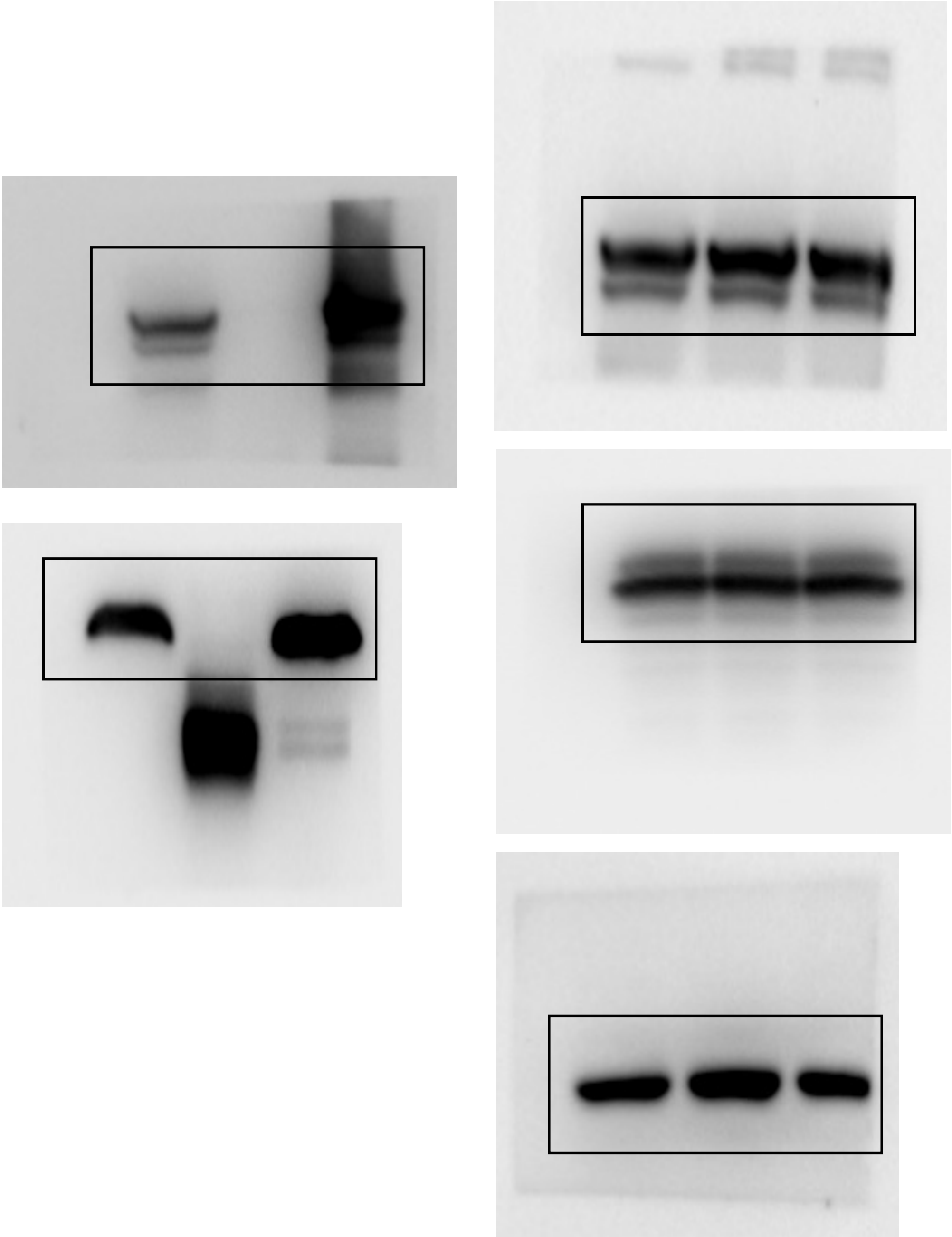

Figure 2

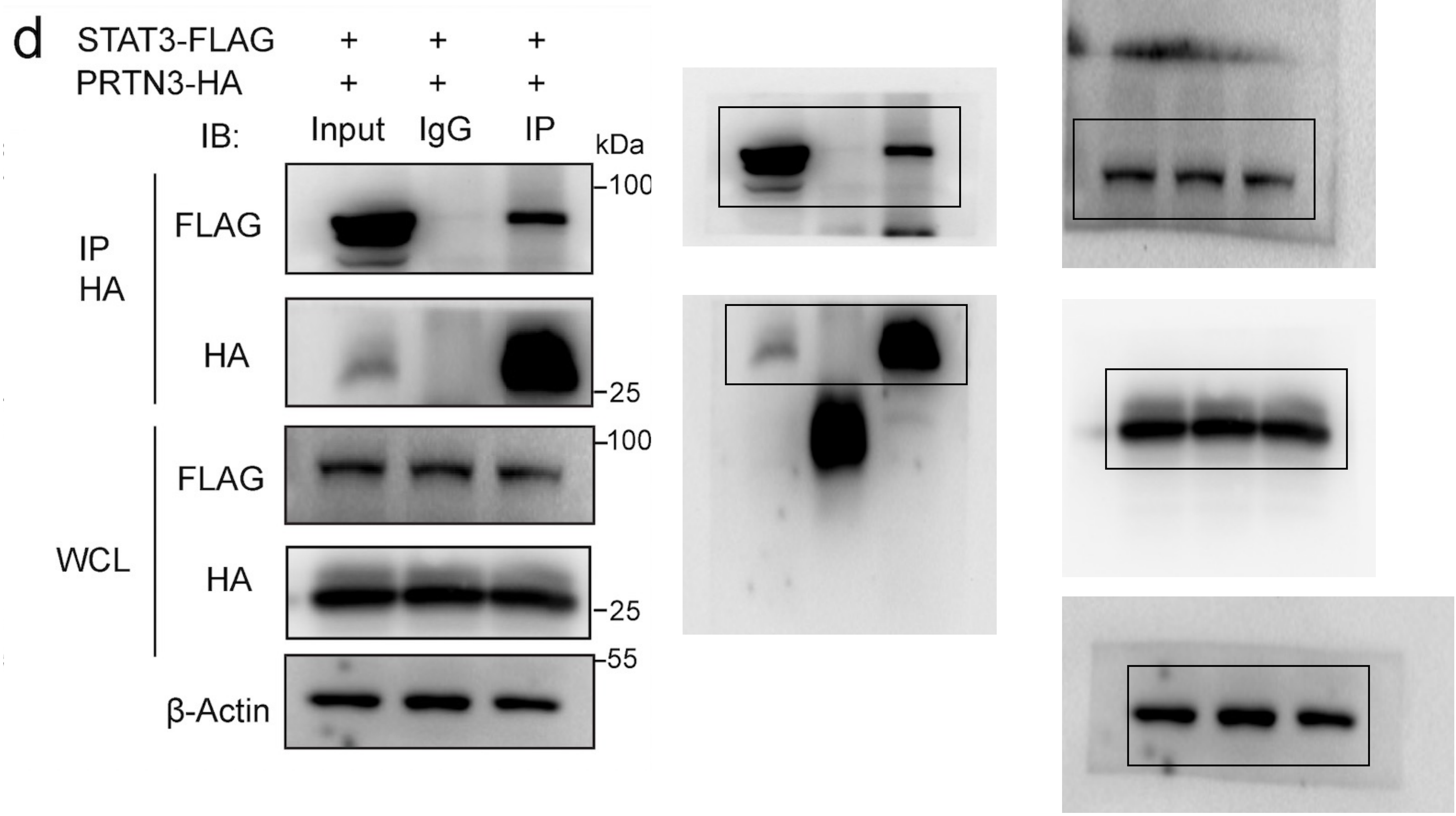
d
STAT3-FLAG + + +
PRTN3-HA + + +
IB:
Input
IgG
IP
kDa
IP
HA
FLAG
HA
WCL
FLAG
HA
β-Actin
100
25
100
25
55

Figure 2

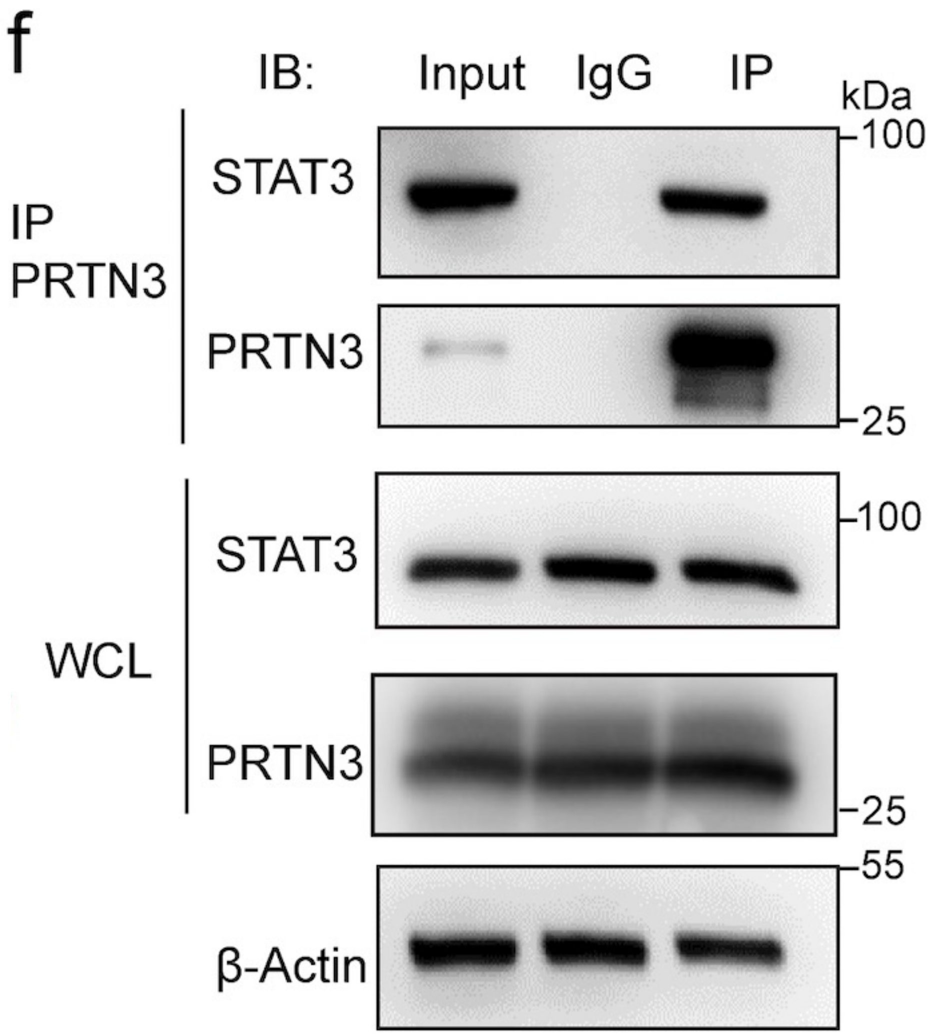

f
IB:
Input
IgG
IP
kDa
100
IP
PRTN3
STAT3
PRTN3
25
100
STAT3
WCL
PRTN3
25
55
β-Actin

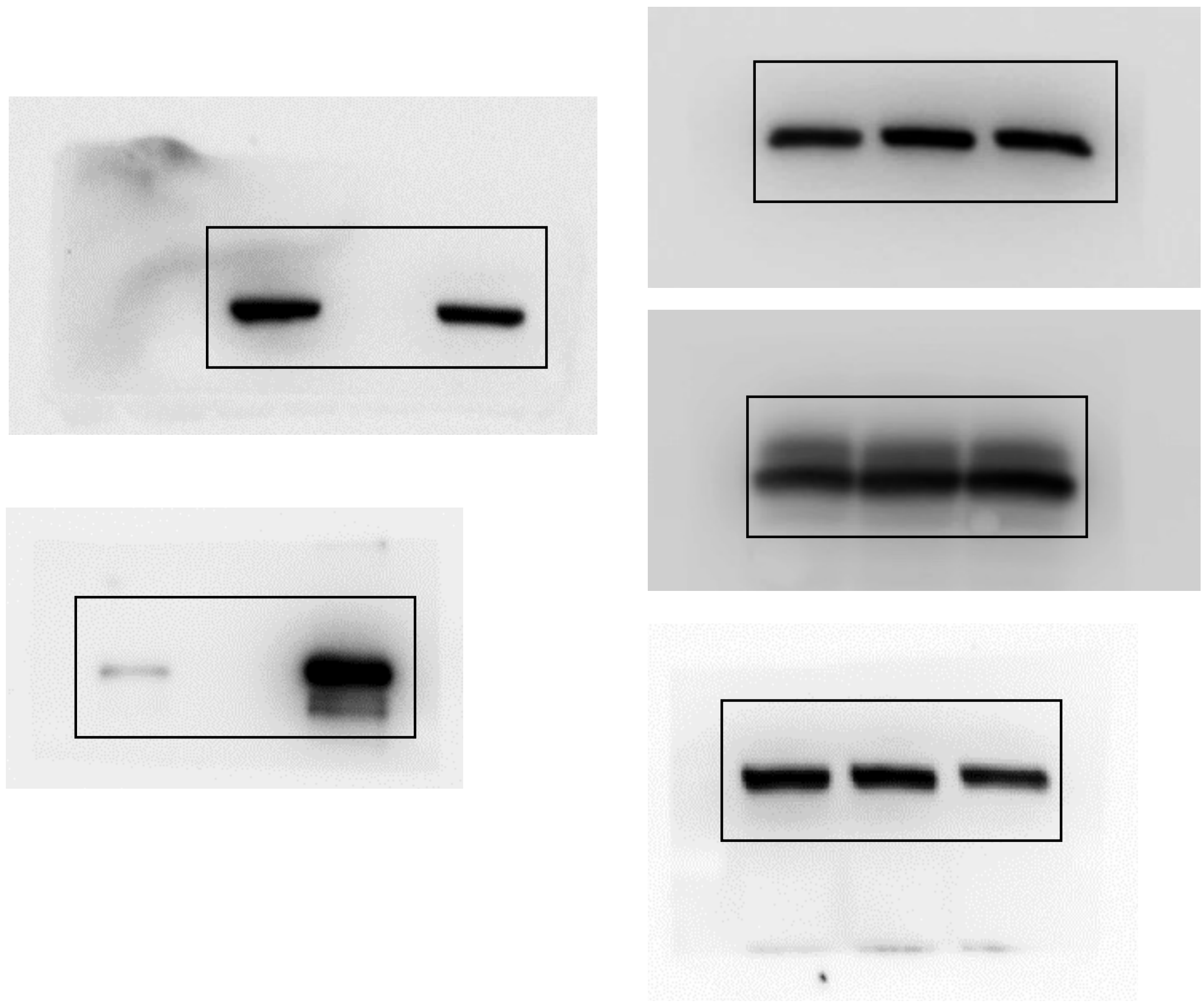

Figure 2

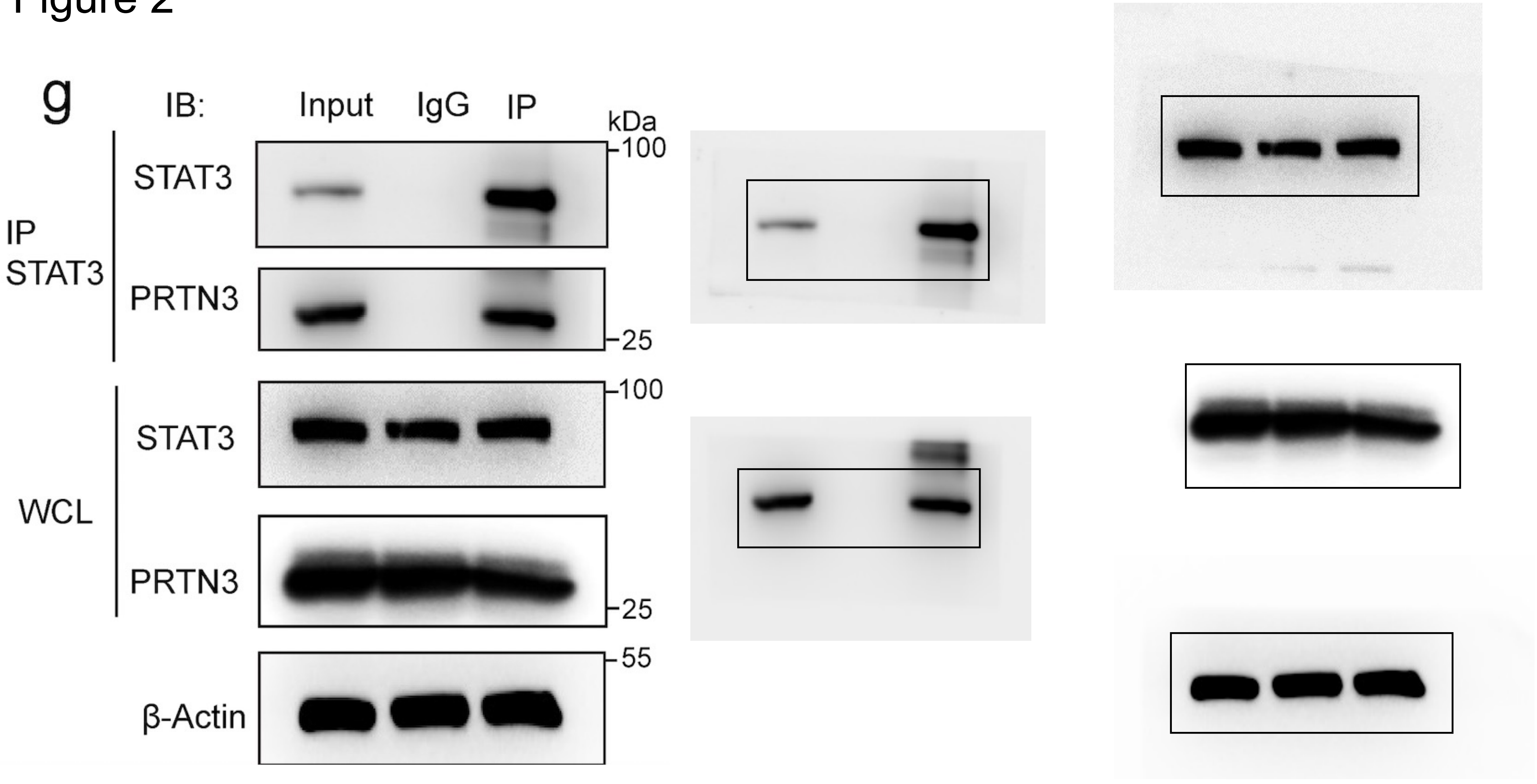
g
IB:
Input
IgG
IP
kDa
100
STAT3
IP
STAT3
PRTN3
25
100
STAT3
WCL
PRTN3
25
55
β-Actin

Figure 3

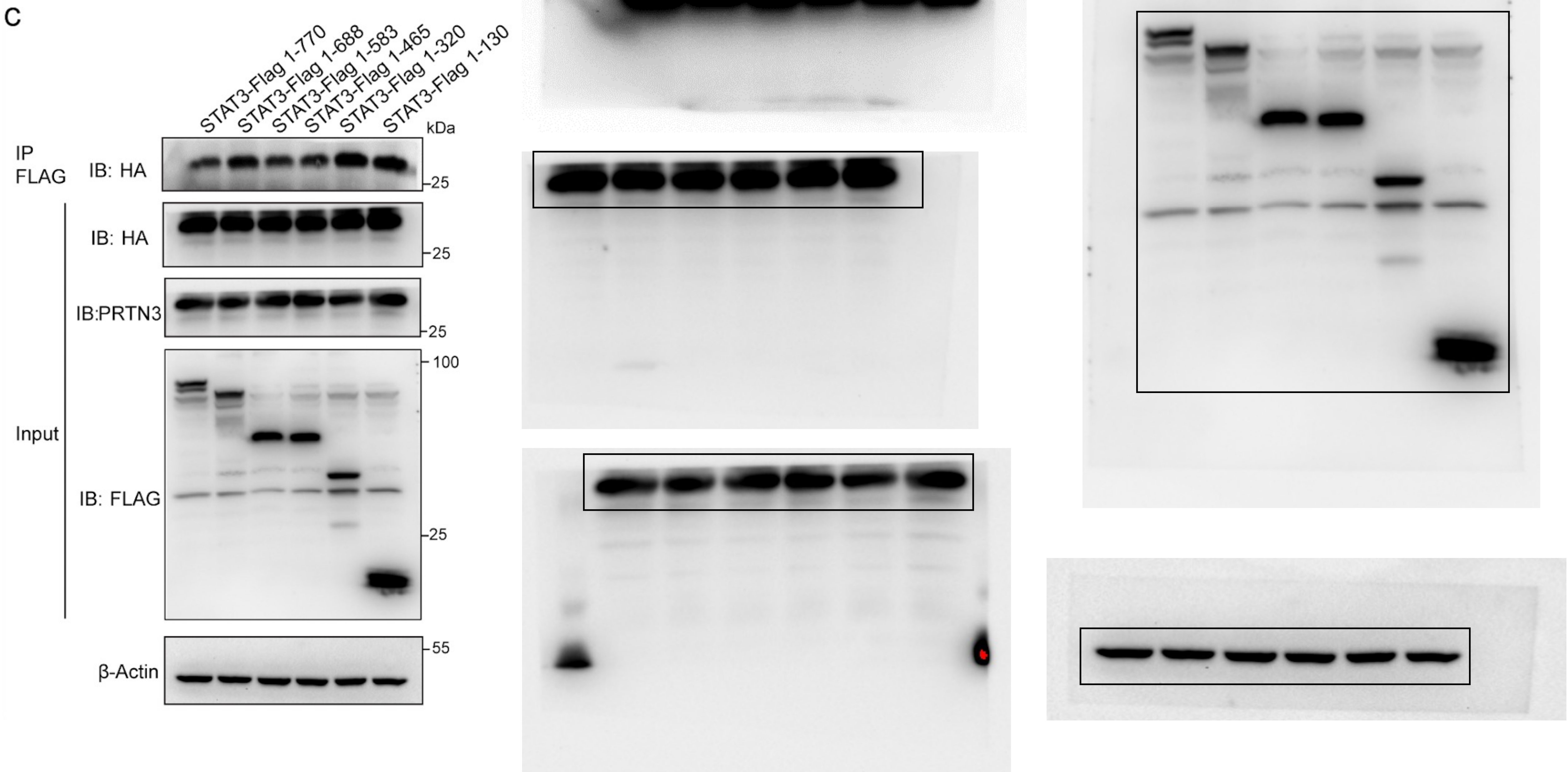
C
STAT3-Flag 1-770
STAT3-Flag 1-688
STAT3-Flag 1-583
STAT3-Flag 1-465
STAT3-Flag 1-320
STAT3-Flag 1-130
kDa
IP
FLAG
IB: HA
25
IB: HA
25
IB:PRTN3
25
Input
100
IB: FLAG
25
β-Actin
55

Figure 3

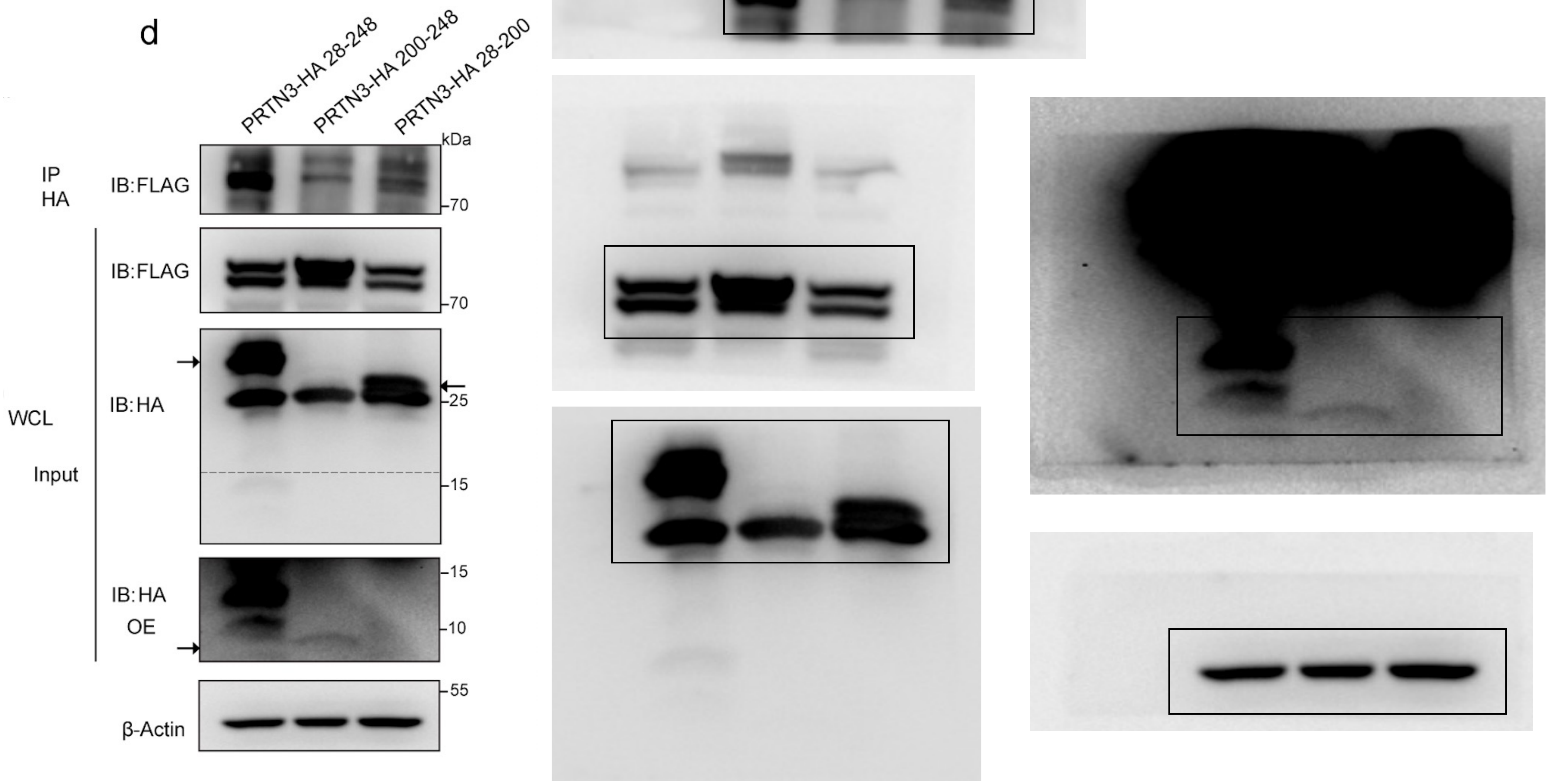

d
PRTN3-HA 28-248
PRTN3-HA 200-248
PRTN3-HA 28-200
kDa
IP
HA
IB:FLAG
70
IB:FLAG
70
WCL
Input
IB:HA
25
15
IB:HA
OE
15
10
β-Actin
55

Figure 3

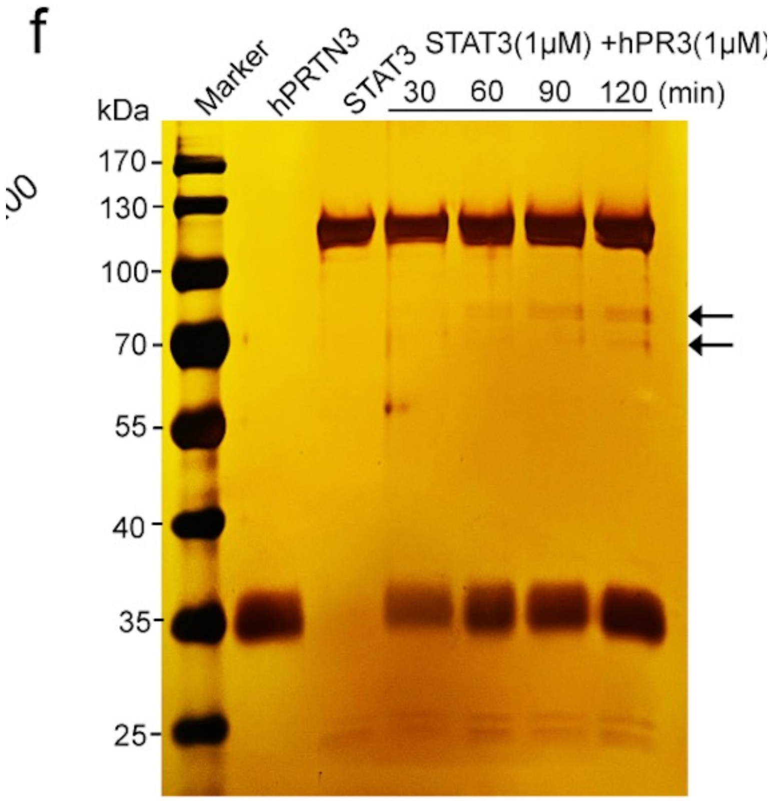

f
kDa
Marker
hPRTN3
STAT3
STAT3(1μM) +hPR3(1μM)
30 60 90 120 (min)
170
130
100
70
55
40
35
25

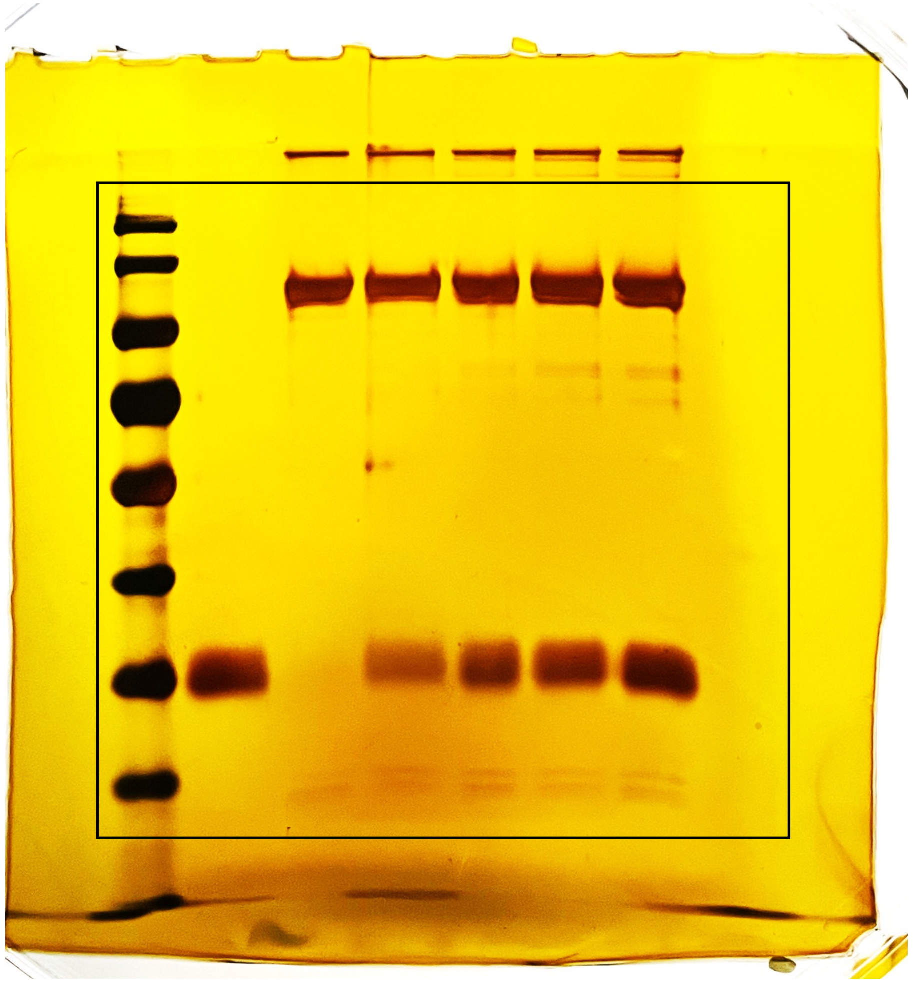

Figure 4

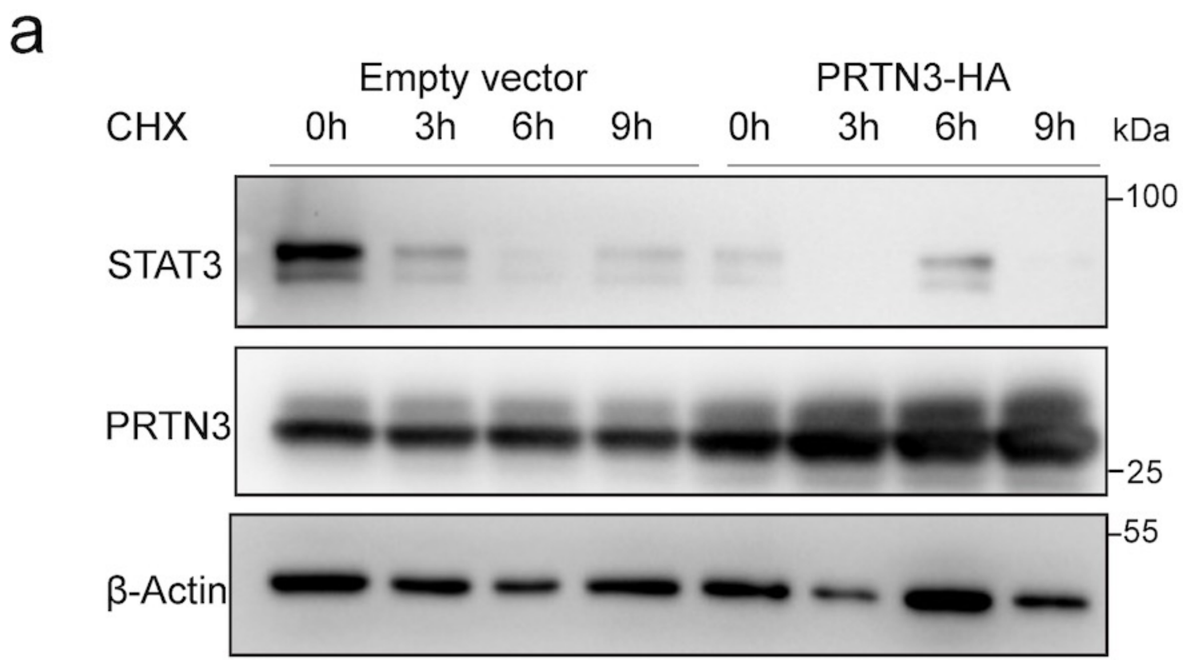
a
Empty vector
PRTN3-HA
CHX
0h
3h
6h
9h
0h
3h
6h
9h
kDa
STAT3
PRTN3
β-Actin
100
25
55

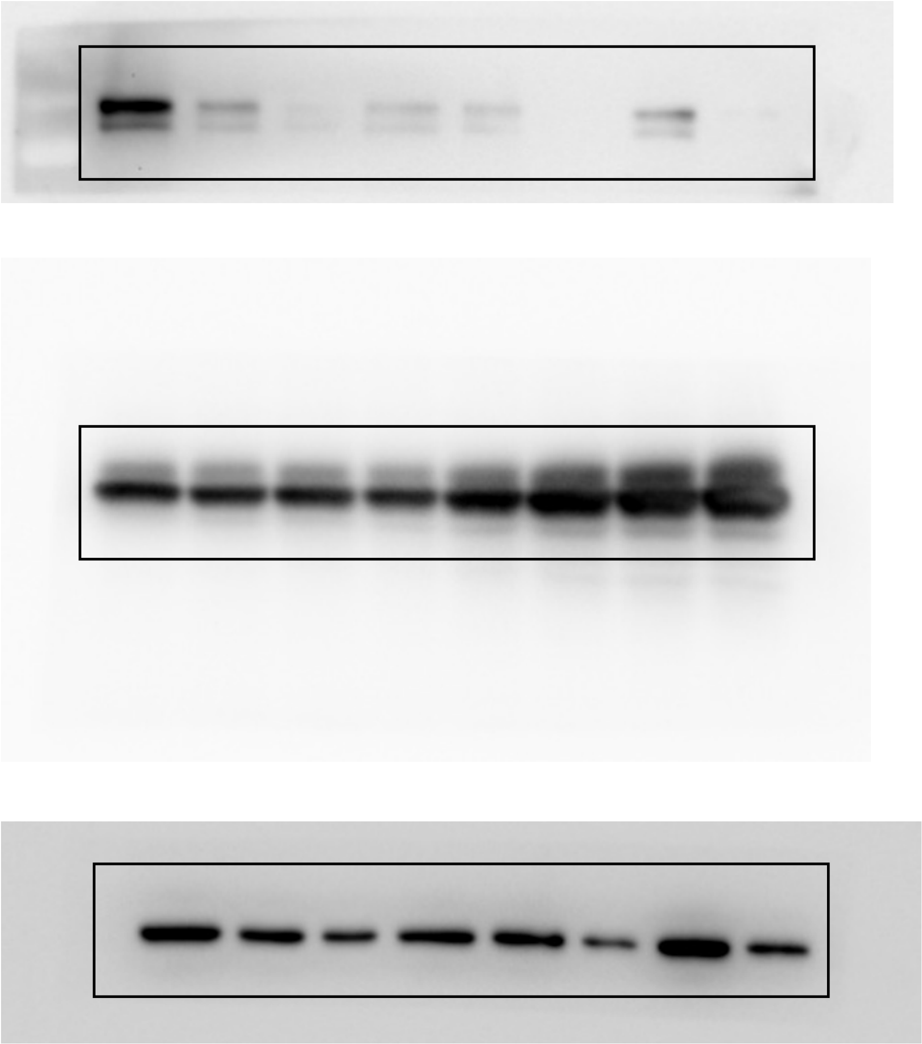

Figure 4

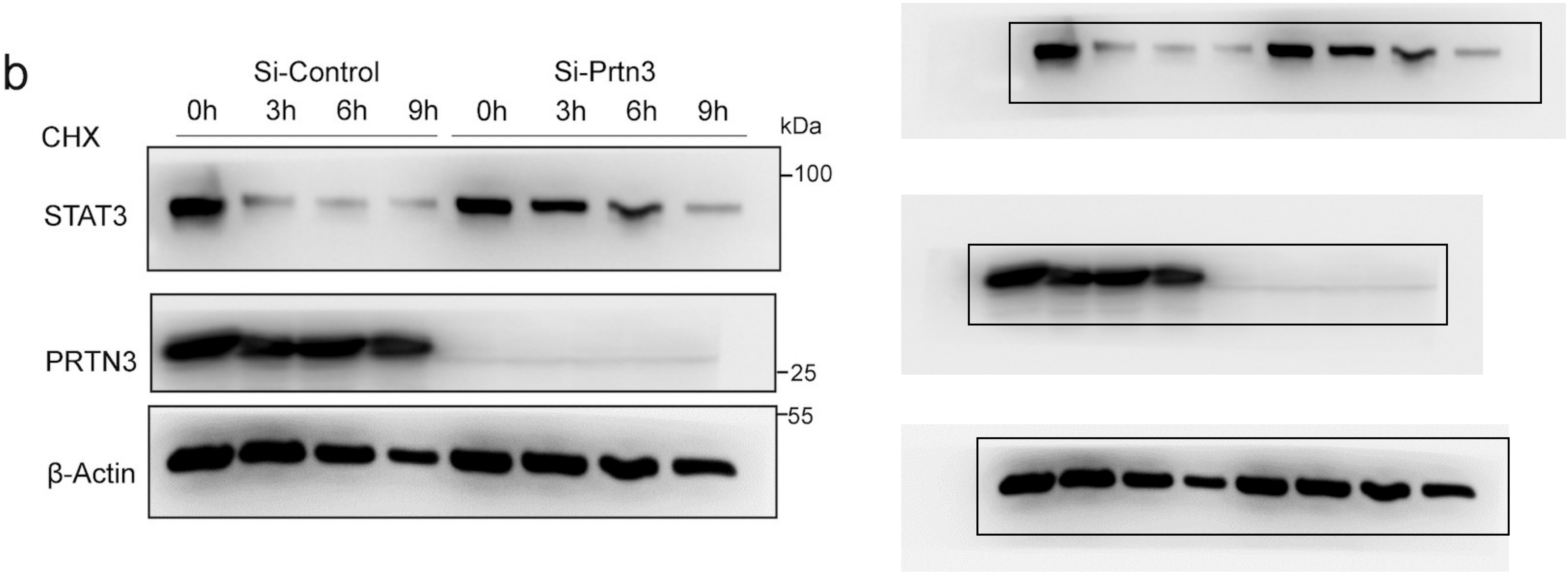
b
Si-Control
Si-Prtn3
0h
3h
6h
9h
0h
3h
6h
9h
CHX
kDa
STAT3
100
PRTN3
25
55
β-Actin

Figure 4

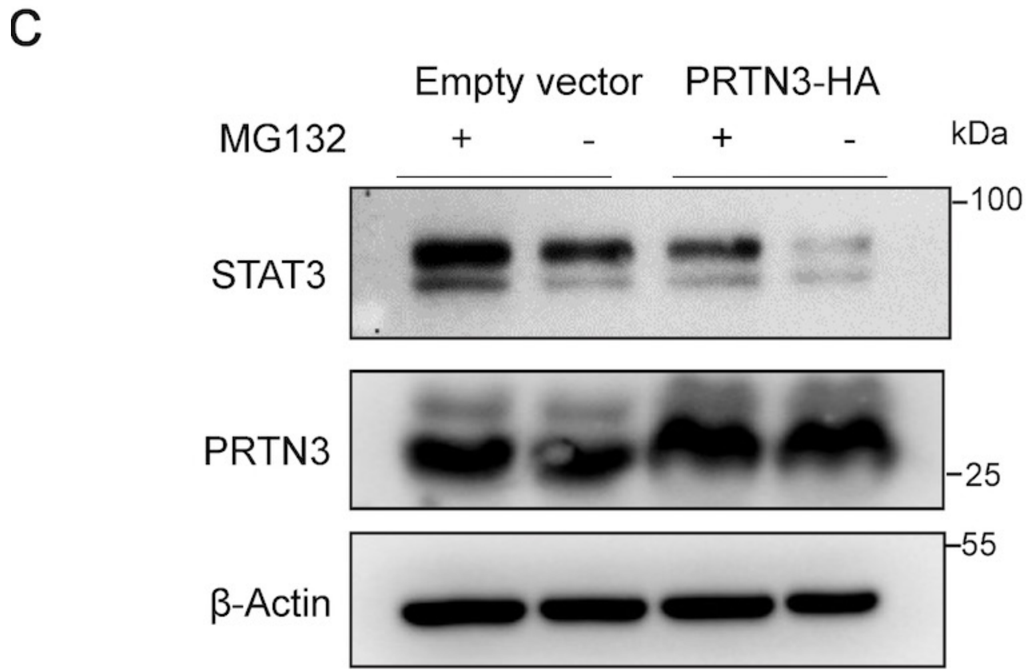
C
Empty vector
PRTN3-HA
MG132
+
-
+
-
kDa
STAT3
PRTN3
β-Actin
100
25
55

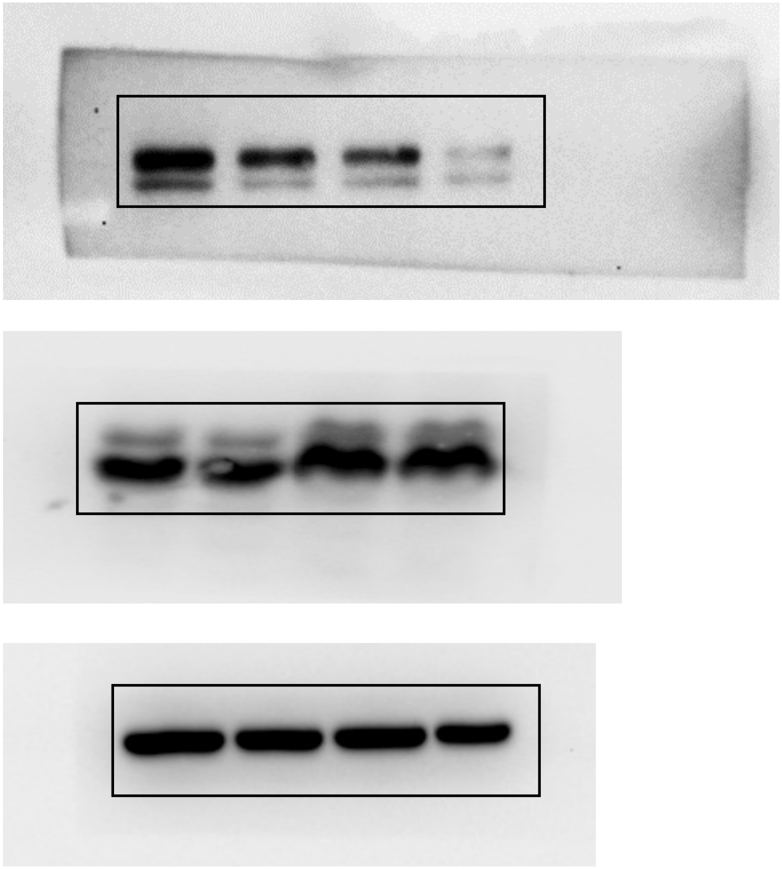

Figure 4

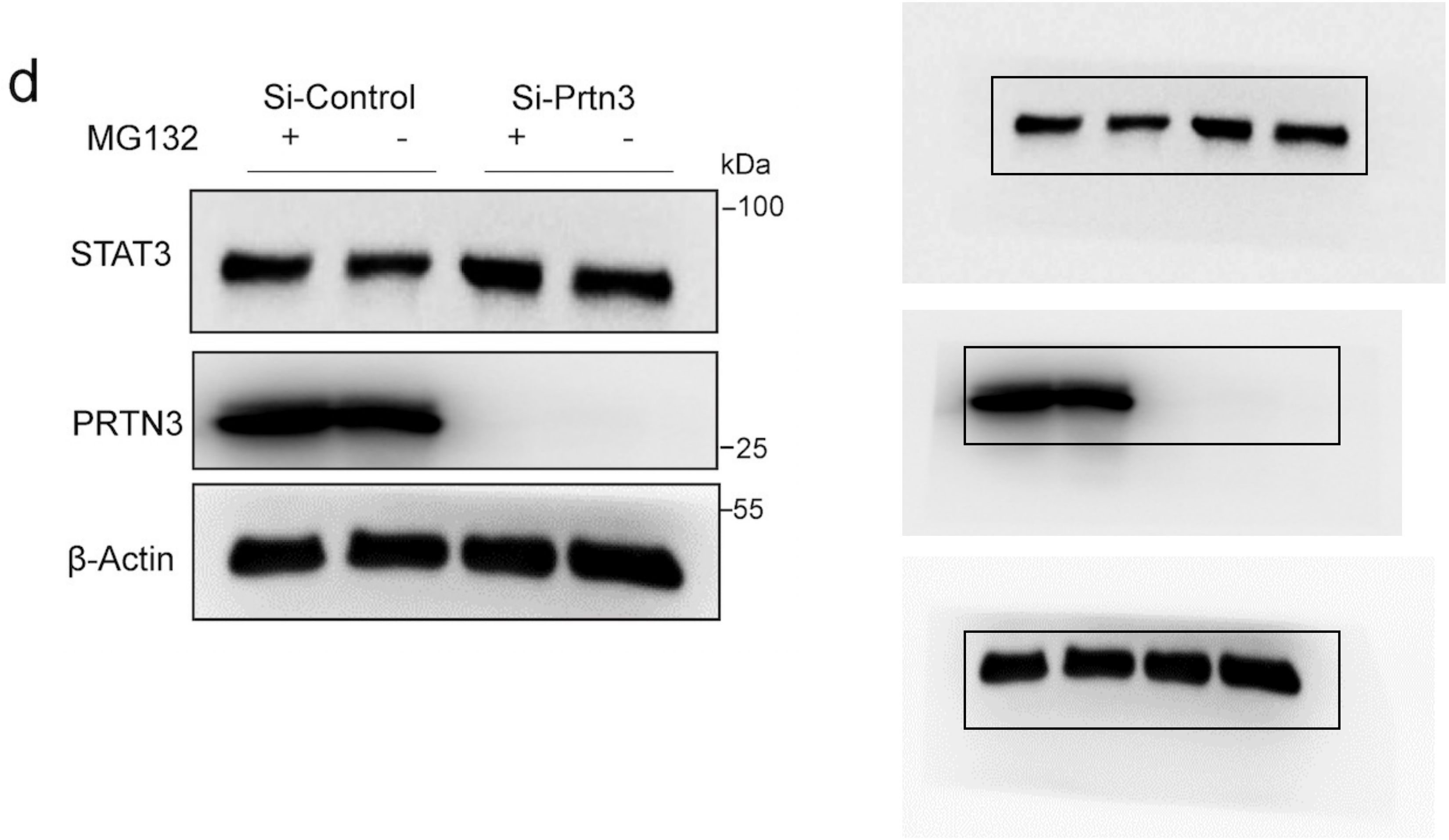

Figure 4

e

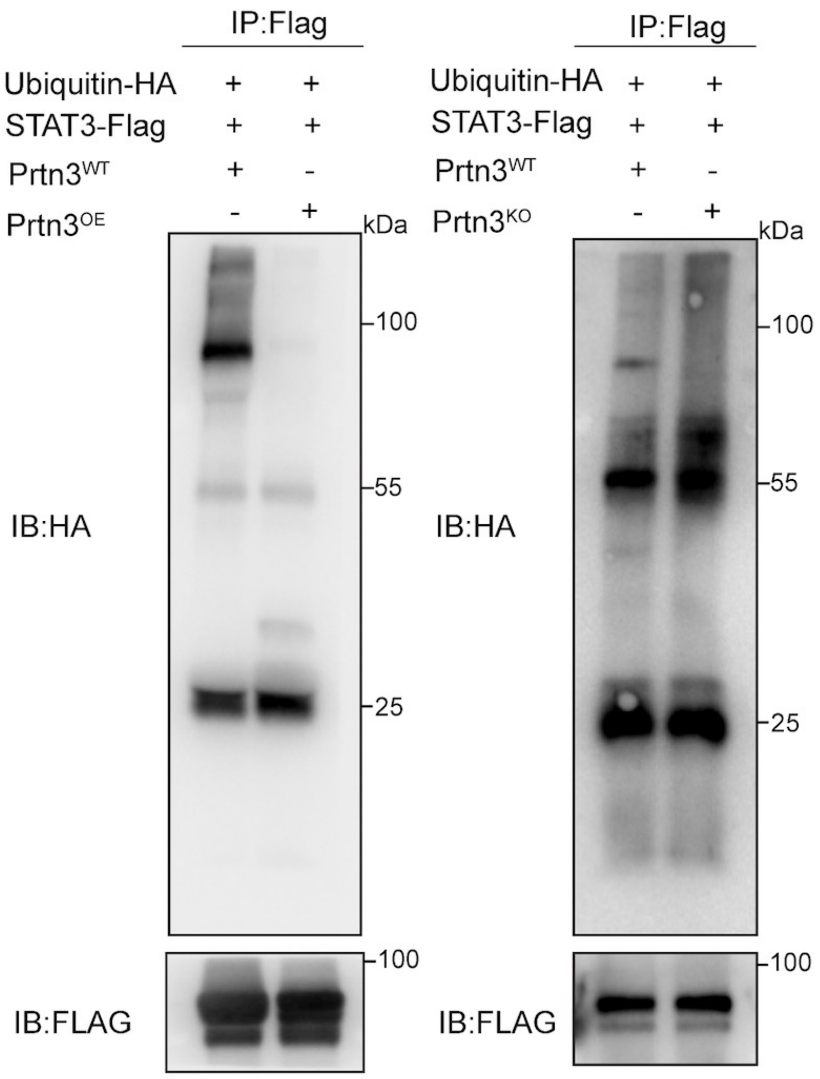

IP:Flag
Ubiquitin-HA + +
STAT3-Flag + +
Prtn3WT + -
Prtn3OE - +
kDa
100
55
25
IB:HA
100
IB:FLAG
IP:Flag
Ubiquitin-HA + +
STAT3-Flag + +
Prtn3WT + -
Prtn3KO - +
kDa
100
55
25
IB:HA
100
IB:FLAG

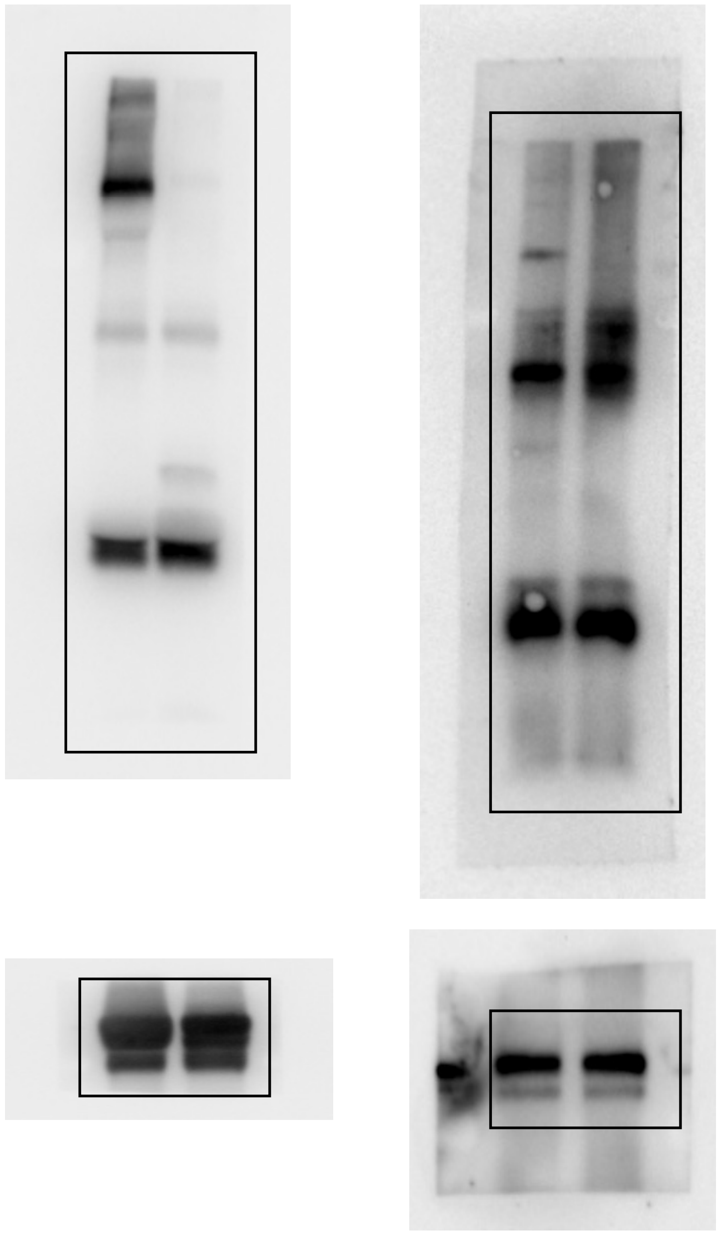

Figure 5

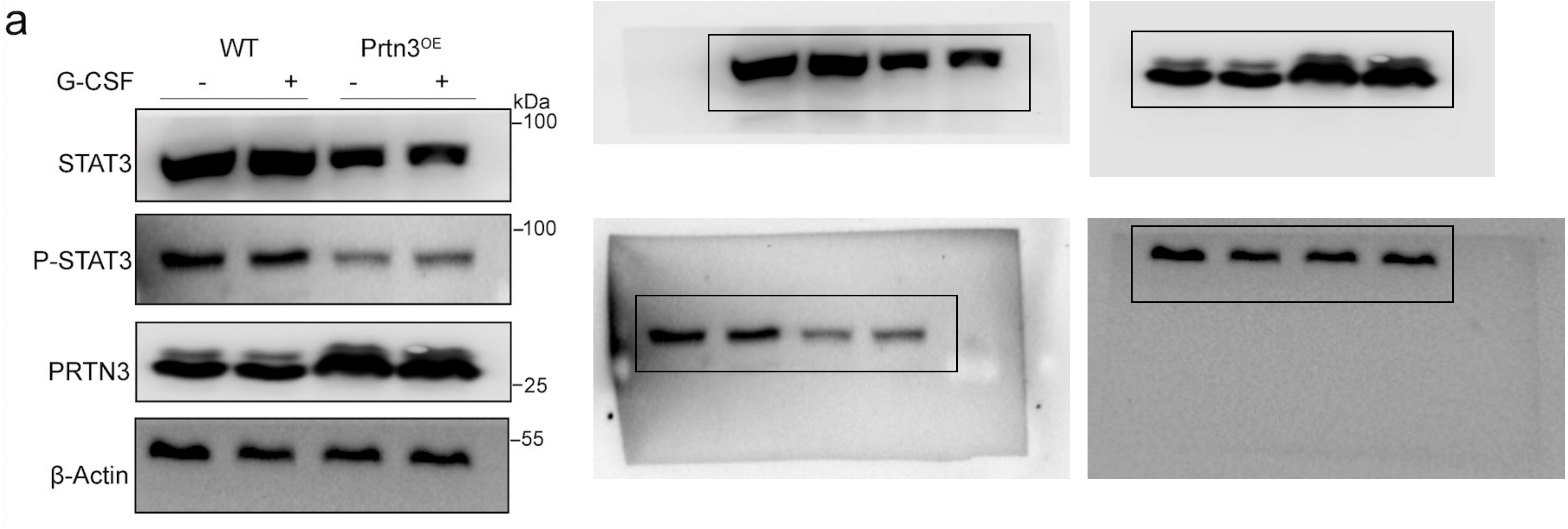

Figure 5

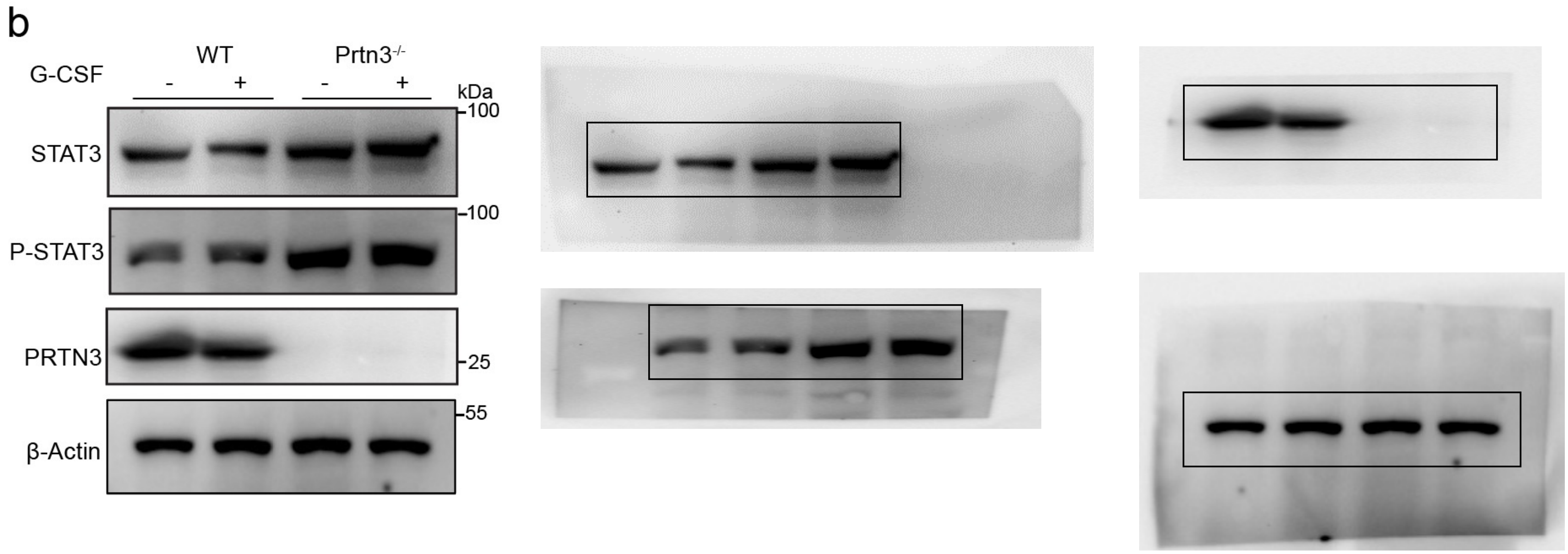

Figure 6

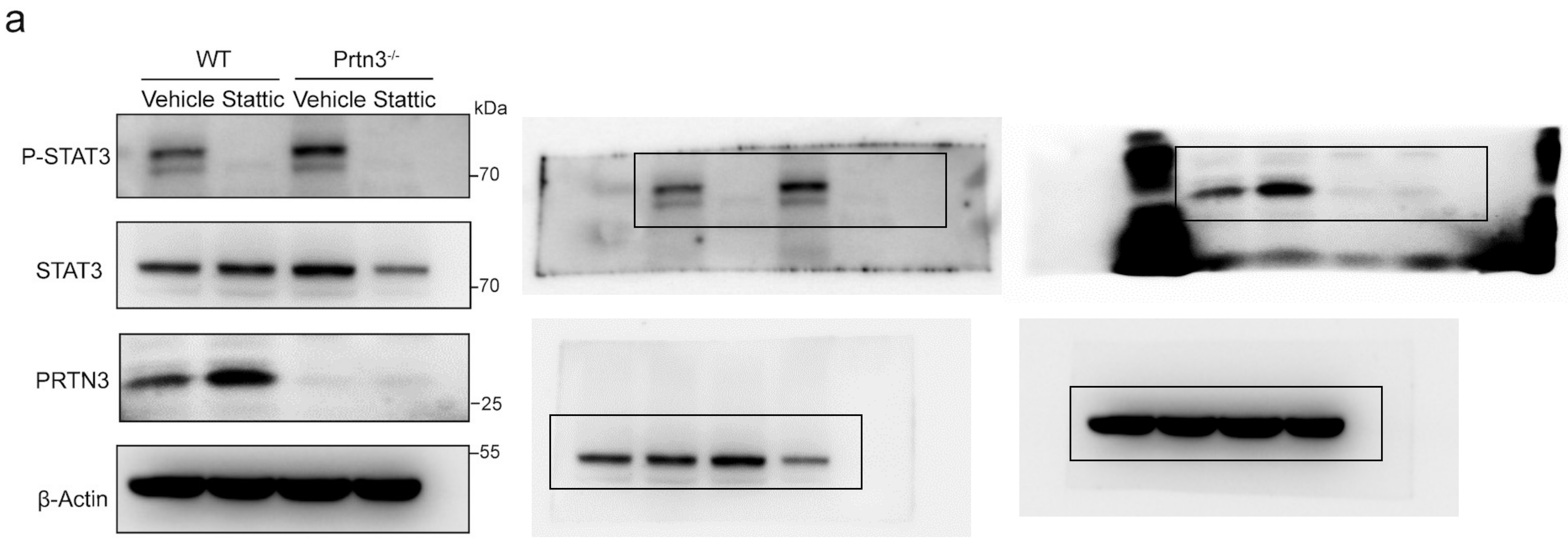
a
WT
Prtn3-/-
Vehicle Stattic Vehicle Stattic
kDa
P-STAT3
70
STAT3
70
PRTN3
25
55
β-Actin

Figure 7

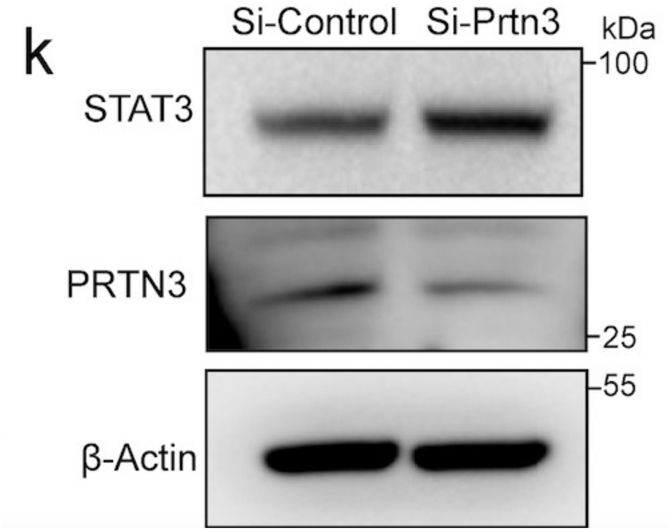

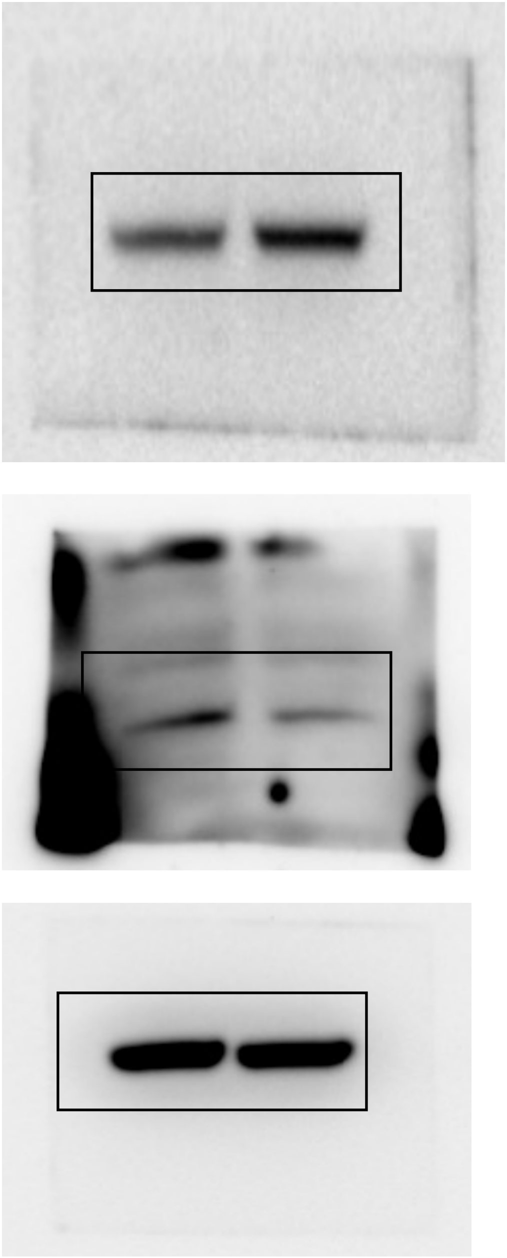

Figure S10

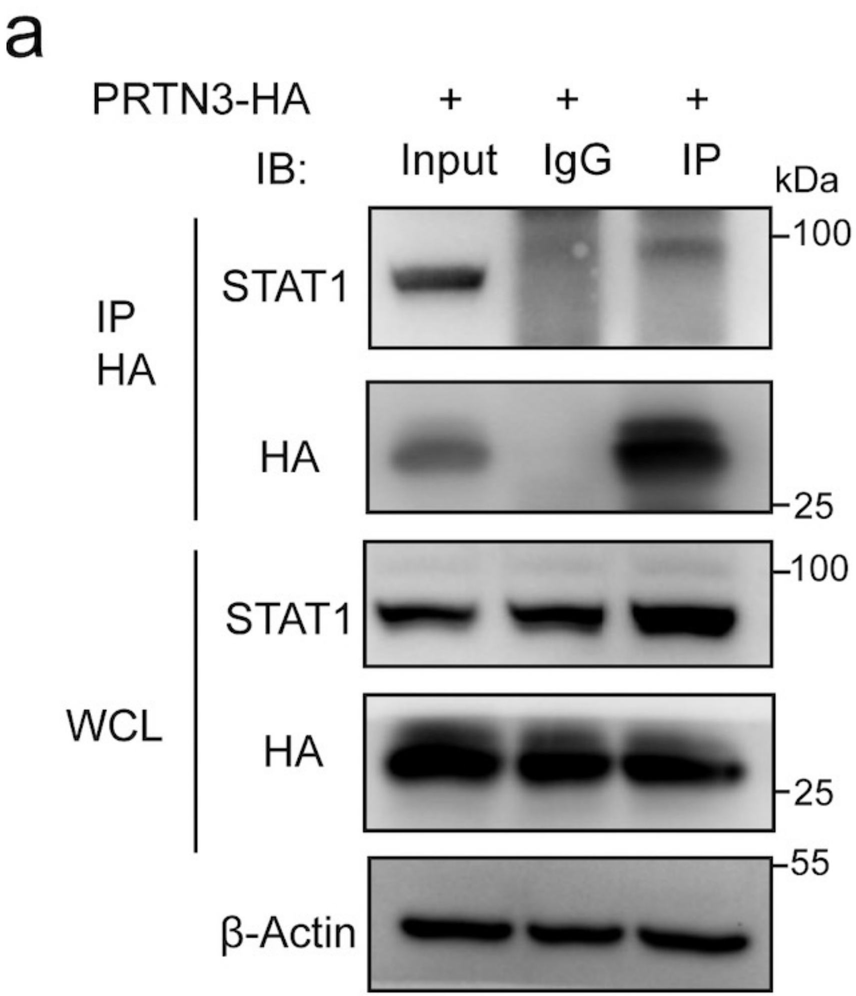

Left

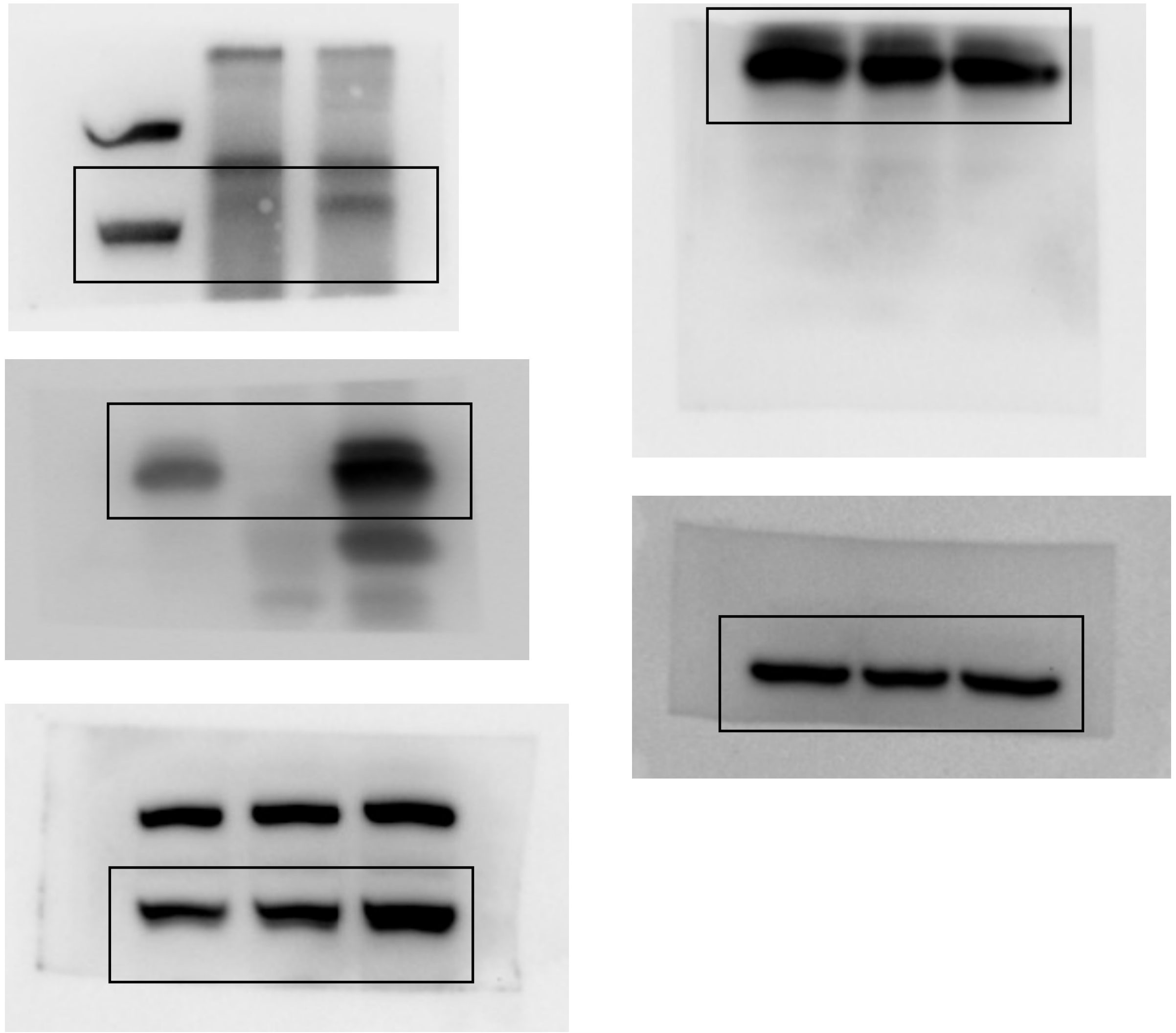

Figure S10 a

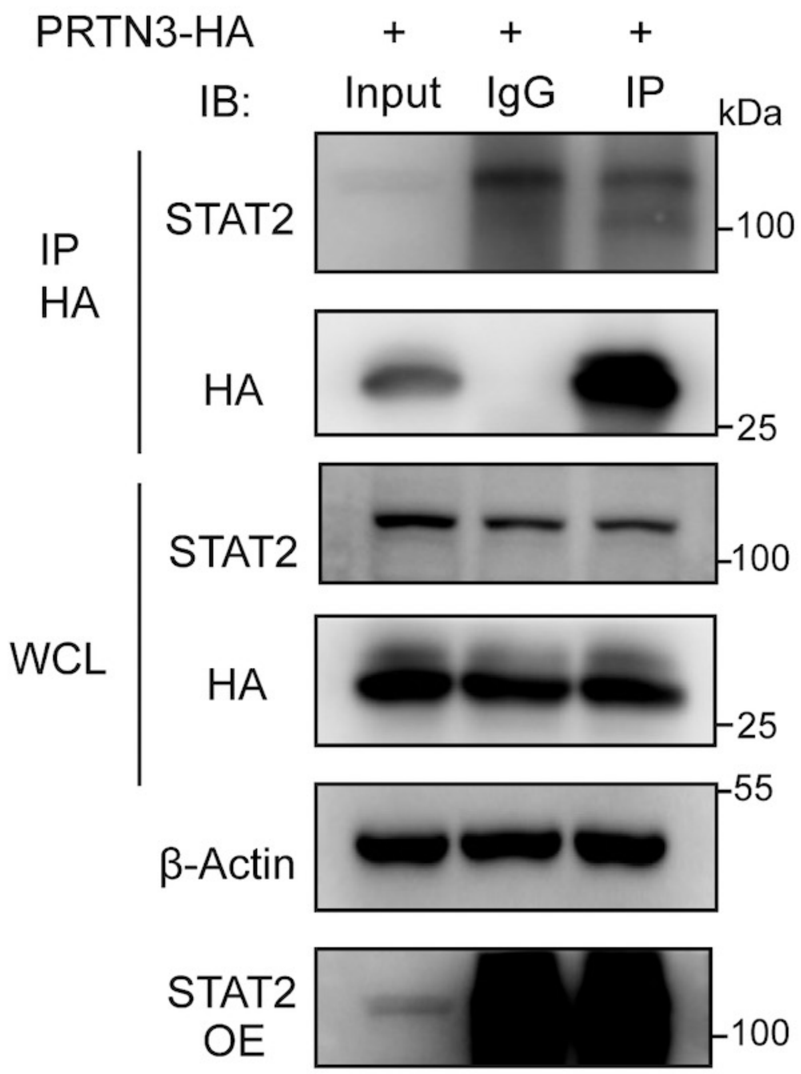

Middle

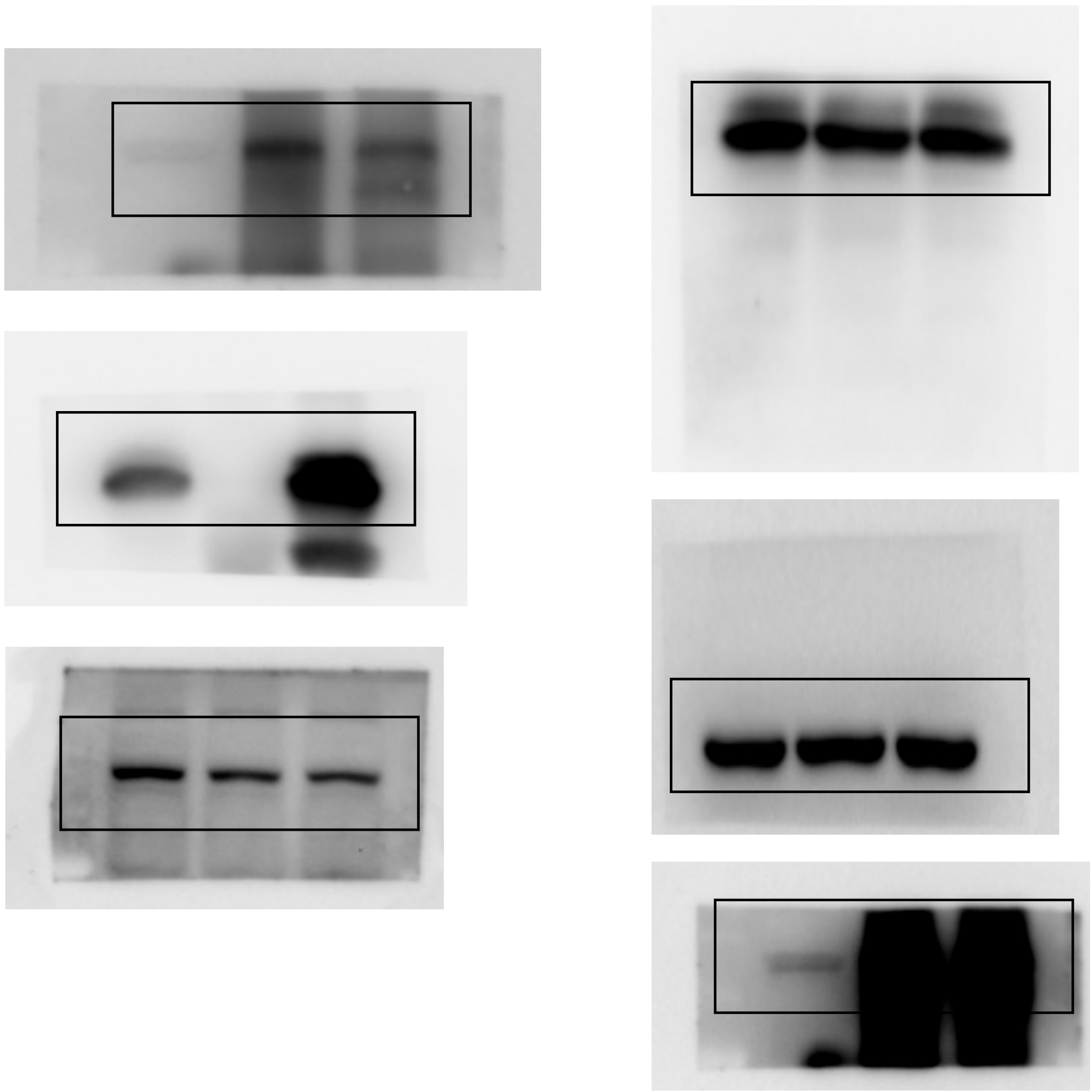

Figure S10 a

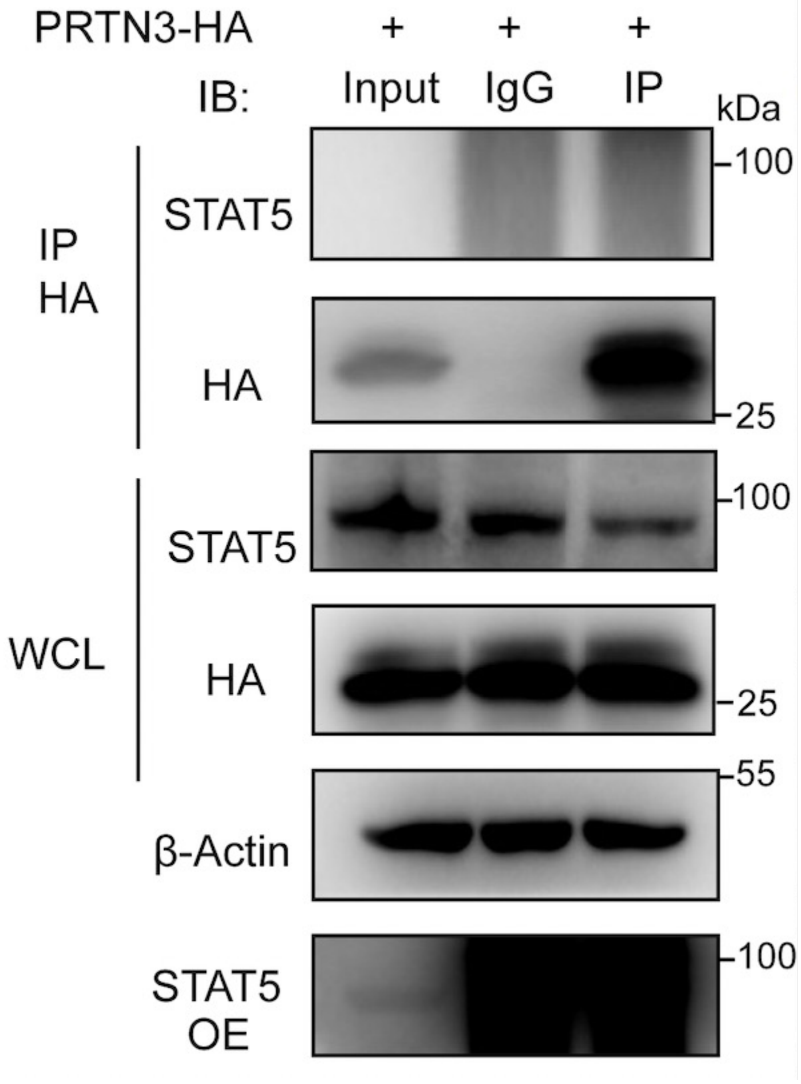

Right

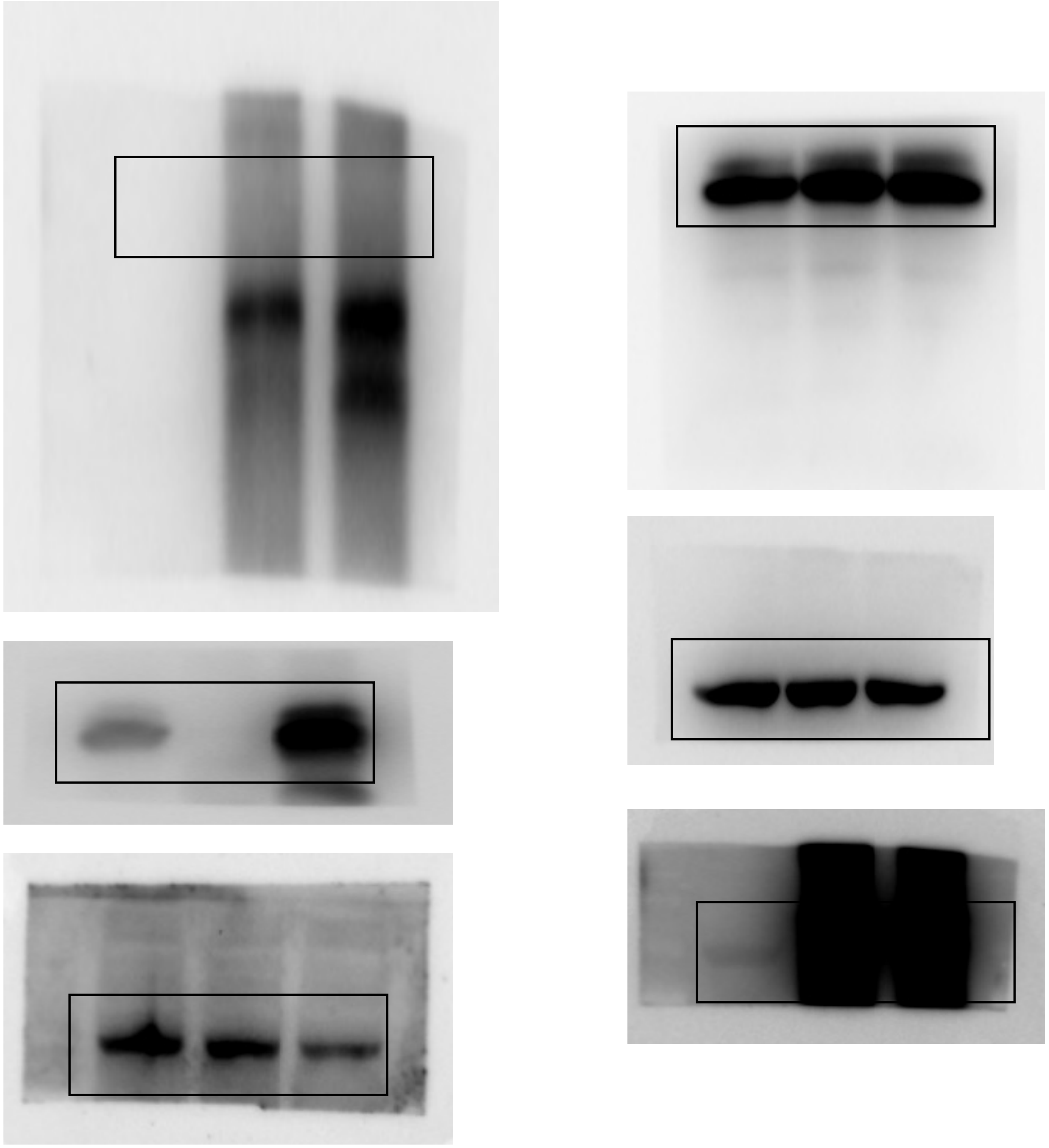

Figure S10

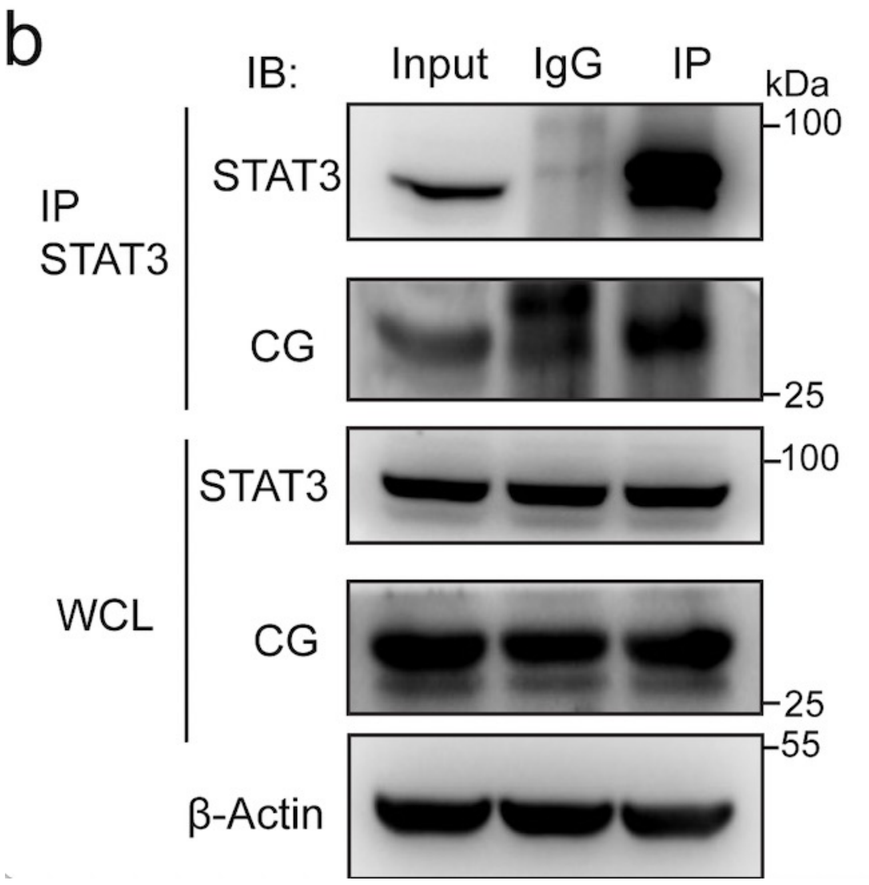

Left

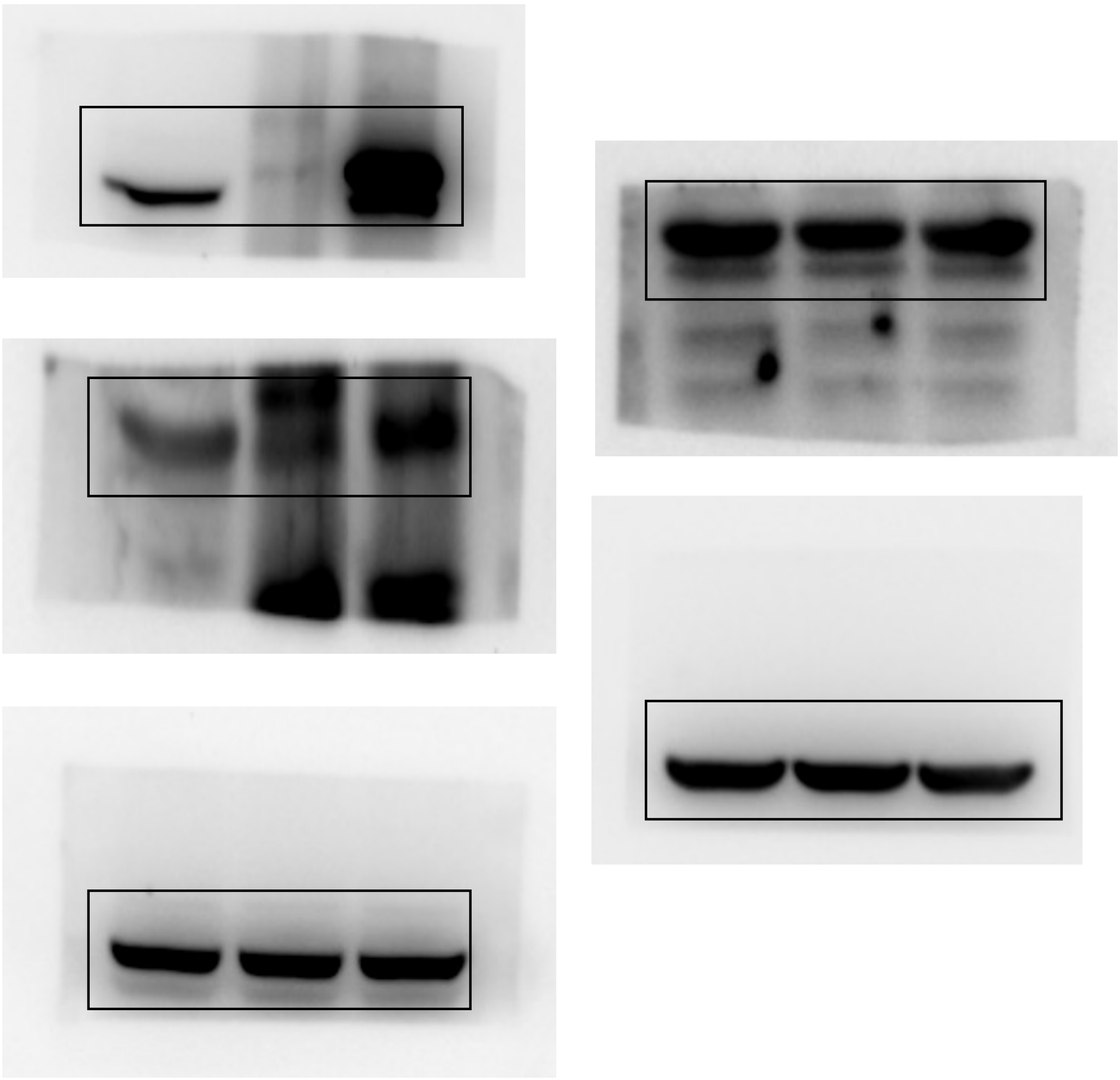

Figure S10 b

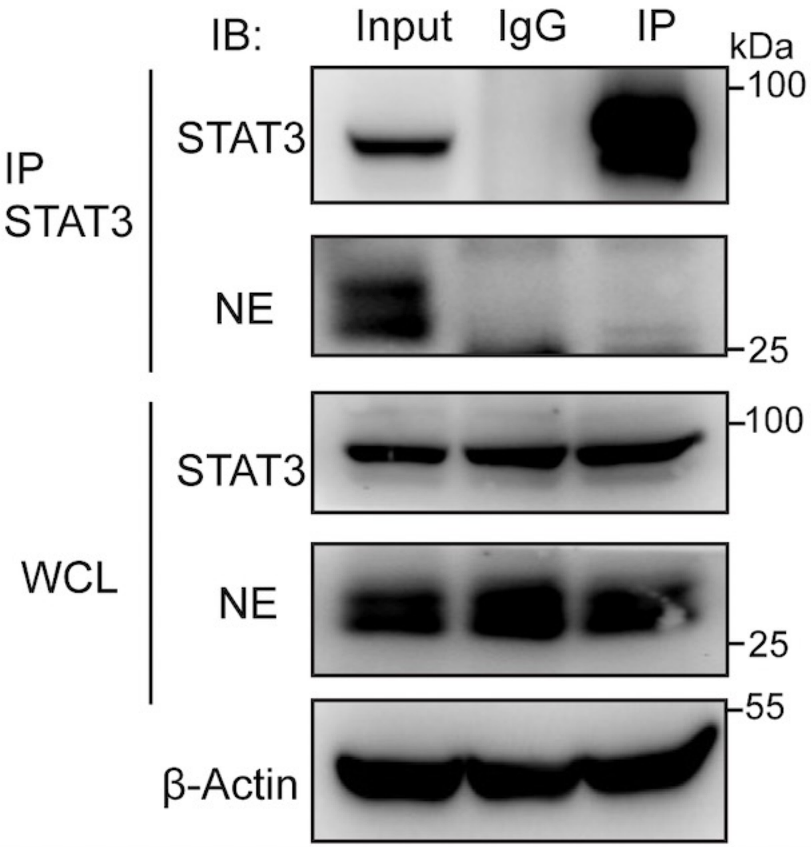

Right

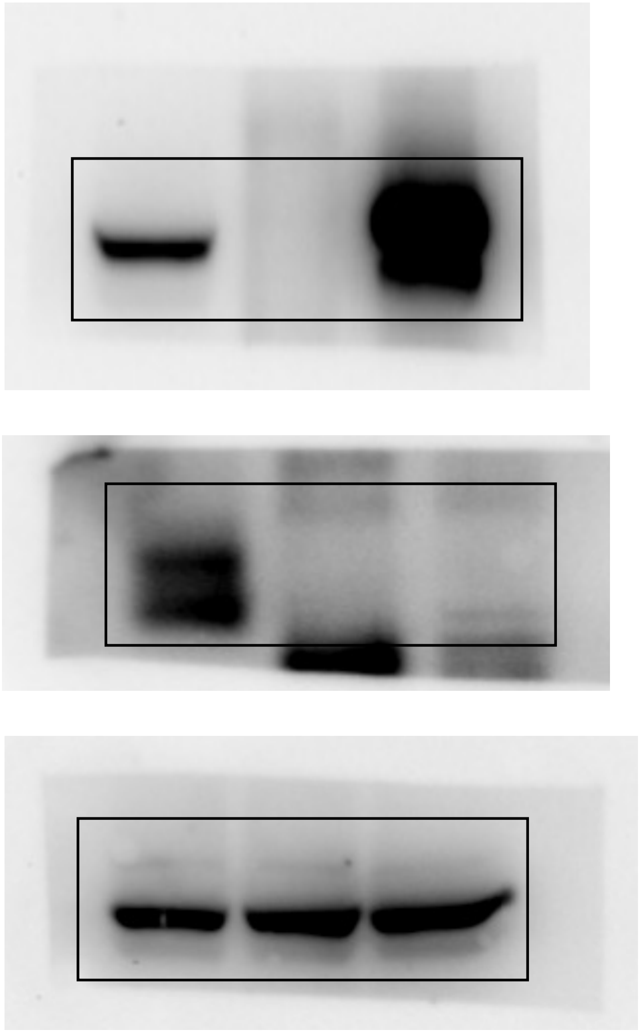
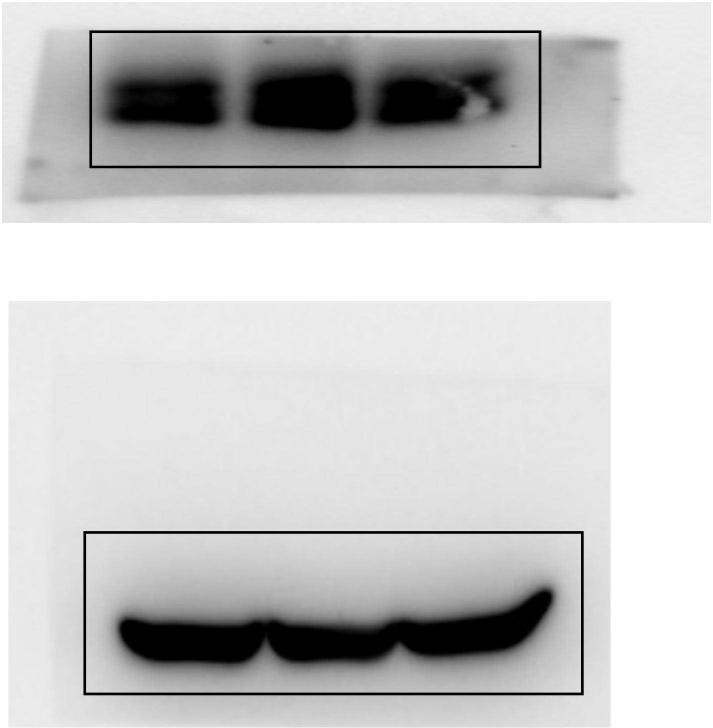

Figure S17

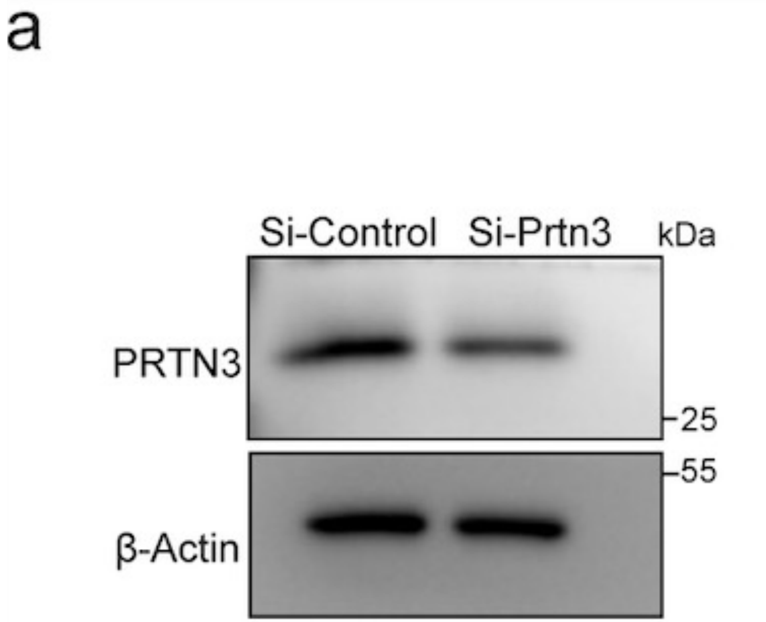

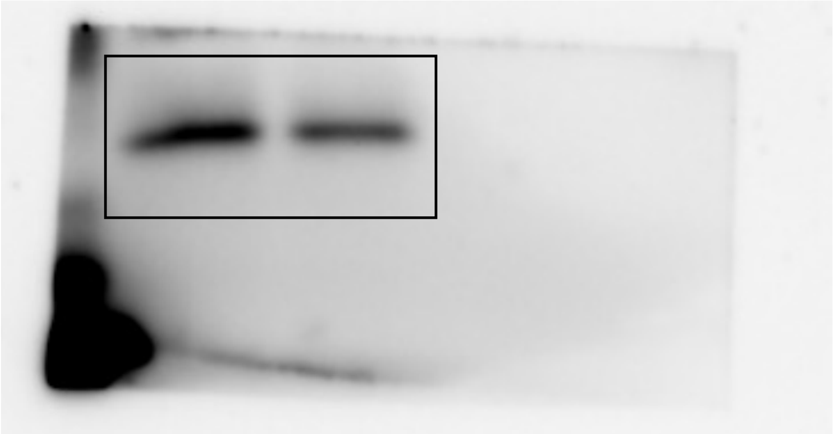

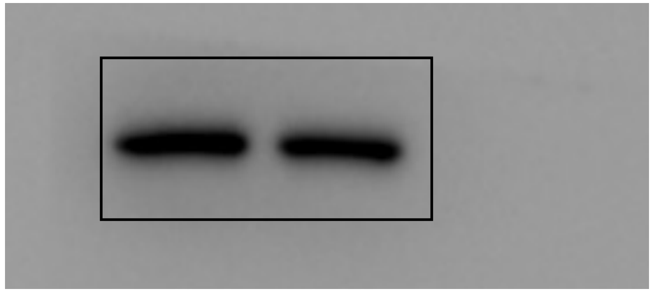

Figure S19

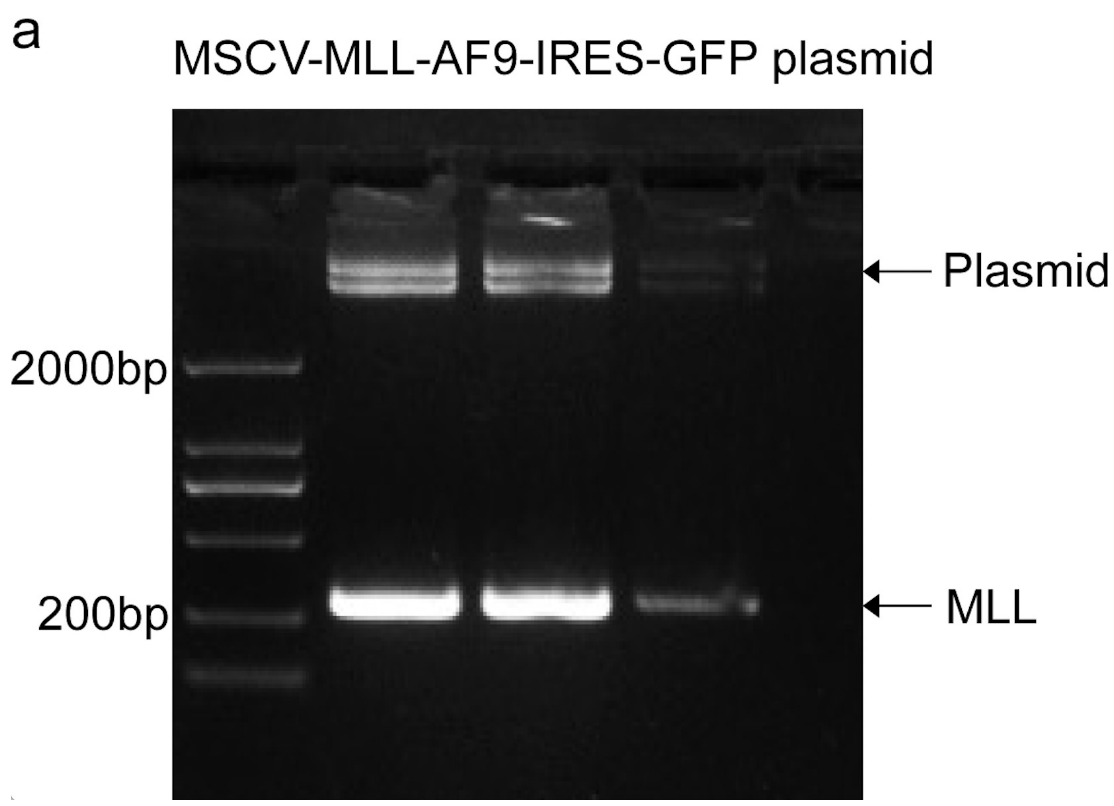

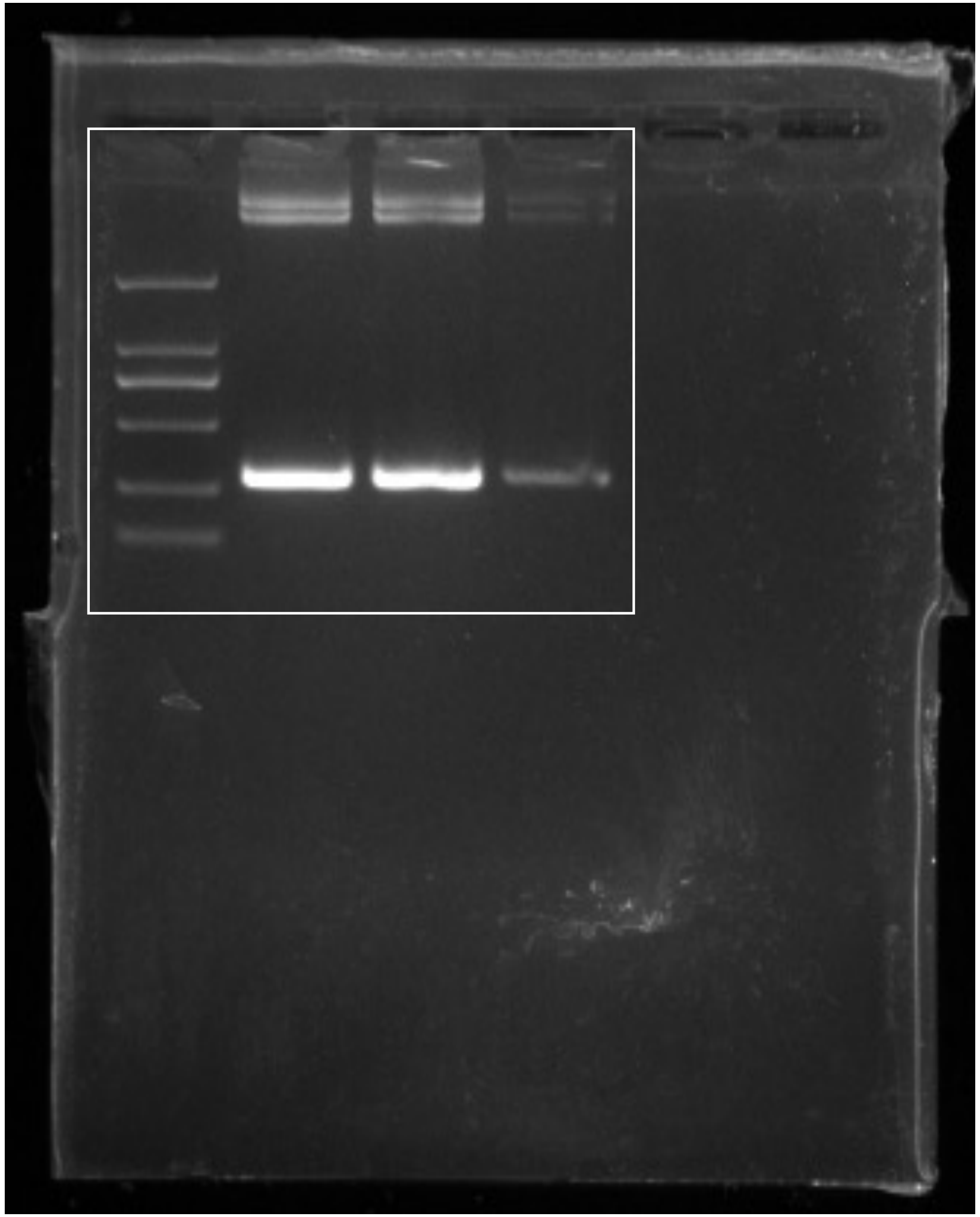

Supplement: Supplementary file 2 — Original Data File [file 41418_2024_1288_MOESM2_ESM.pdf]
